# Identification of N6-Methyladenosine-Related LncRNAs for Predicting Overall Survival and Clustering of a Potentially Novel Molecular Subtype of Breast Cancer

**DOI:** 10.3389/fonc.2021.742944

**Published:** 2021-10-15

**Authors:** Xiaoxiao Zhong, Jun Li, Xin Wu, Xianrui Wu, Lin Hu, Boni Ding, Liyuan Qian

**Affiliations:** ^1^ Department of Breast and Thyroid Surgery, Third Xiangya Hospital, Central South University, Changsha, China; ^2^ Department of Spine Surgery, Third Xiangya Hospital, Central South University, Changsha, China; ^3^ Department of Plastic and Cosmetic Surgery, Third Xiangya Hospital, Central South University, Changsha, China; ^4^ Department of Anesthesiology, Third Xiangya Hospital, Central South University, Changsha, China

**Keywords:** breast cancer, m6A modification, lncRNA, prognostic model, molecular subtype, tumor immune microenvironment, ceRNA network

## Abstract

We aimed to identify a signature comprising N6-methyladenosine (m6A)-related long non-coding RNAs (lncRNAs) and molecular subtypes associated with breast cancer (BRCA). We obtained data of BRCA samples from The Cancer Genome Atlas database. The m6A-related lncRNA prognostic signature (m6A-LPS) included 10 lncRNAs previously identified as prognostic m6A-related lncRNAs and was constructed using integrated bioinformatics analysis and validated. Accordingly, a risk score based on the m6A-LPS signature was established and shown to confirm differences in survival between high-risk and low-risk groups. Three distinct genotypes were identified, whose characteristics included features of the tumor immune microenvironment in each subtype. Our results indicated that patients in Cluster 2 might have a worse prognostic outcome than those in other clusters. The three genotypes and risk subgroups were enriched in different biological processes and pathways, respectively. We then constructed a competing endogenous RNA network based on the prognostic m6A-related lncRNAs. Finally, we validated the expression levels of target lncRNAs in 72 clinical samples. In summary, the m6A-LPS and the potentially novel genotype may provide a theoretical basis for further study of the molecular mechanism of BRCA and may provide novel insights into precision medicine.

## Introduction

Breast cancer (BRCA) is the most common cancer worldwide and a major contributor to cancer-related death in women. The increasing incidence and therapeutic challenges due to its heterogeneity have made BRCA a global burden (1, 2), especially when triple-negative BRCA (TNBRCA) lacks corresponding molecular targets for therapy (3). Patients with poor prognosis require urgent new therapies; molecular subtype and tumor burden need to be considered in subsequent precision therapy concepts (1).

Among multiple modifications of RNA, N6-methyladenosine (m6A) is the most widespread modification of mRNA and involves all steps of RNA metabolism. It can alter the expression of target genes, making them important regulators of tumor processes (4). Regulators of m6A are divided into three types: writers, readers, and erasers. m6A is a reversible and dynamic RNA modification; crosslinking among the three types of m6A regulators is involved in cancer pathogenesis and progression (5). Recently, many studies have reported advances in understanding the underlying mechanisms of m6A modification in oncogenesis and progression of cancer, including BRCA. For example, ectopic expression of ALKBH5 promotes enrichment of BRCA stem cells under hypoxic conditions (6). By suppressing the upregulation of PD-1, CXCR4, and SOX10 mediated by YTHDF2-degradation, FTO inhibits IFNγ-induced killing and the response of anti-PD-1 blockade immunotherapy in melanoma cells (7). METTL3 has oncogenic effects by modulating nonsense-mediated mRNA decay of splicing factors and alternative splicing isoform switches in glioblastoma (8). Bioinformatics research has indicated that aberrations of m6A regulators are associated with poor prognosis of BRCA (9).

Long non-coding RNAs (lncRNAs), defined as transcripts longer than 200 nucleotides, do not encode proteins. Aberration of lncRNA expression appears to play major roles in cancer, such as promoting tumorigenesis and metastasis (10); for example, lncRNA BRCART1 promotes BRCA progression by targeting the microRNA (miR)-1303/PTBP3 axis (11); activation of lncRNA TINCR by H3K27 acetylation promotes trastuzumab resistance and epithelial-mesenchymal transition by targeting miR-125b in BRCA (12). m6A modification in non-coding RNAs has been reported to play a critical role in major normal bioprocesses, and many lncRNAs modified by m6A have been discovered (13). However, the effect of the interaction between m6A modification and non-coding RNAs in tumors remains unclear, and the potential mechanisms of m6A modification of lncRNAs that affect BRCA progression have scarcely been studied. LncRNAs regulated by m6A modification have not yet been identified in BRCA, and they may provide effective therapeutic targets to improve patient prognosis.

In this study, we integrated bioinformatics analyses to identify and validate a robust and stable molecular signature for survival prediction in BRCA. We constructed and verified the prognostic significance of a risk-scoring model based on 10 m6A-related lncRNAs. Based on the m6A-related prognostic lncRNAs, we divided BRCA samples from The Cancer Genome Atlas (TCGA) into different genotypes and explored the differences between the clusters. Furthermore, we constructed a competing endogenous RNA (ceRNA) network to forecast the target miRNAs and mRNAs of these m6A-related prognostic lncRNAs and attempted to predict the potential function of these lncRNAs through functional and pathway enrichment analyses of target mRNAs.

## Materials and Methods

### Data Source and Processing

From the TCGA website (https://cancergenome.nih.gov/), RNA expression files (Fragments Per Kilobase of transcript per Million mapped reads [FPKM] normalized), and the corresponding clinical data of BRCA samples and normal samples were obtained. To reduce bias in the statistical analysis in our study, BRCA patients with missing overall survival (OS) values or a follow-up time < 30 days and male BRCA patients were excluded. Finally, we acquired data from 1022 BRCA patients from TCGA. Perl5.30.1 software was used to merge the RNA expression values of each sample into a total matrix file. A list of 23 m6A-related genes was collected based on previous publications, including writers (METTL3, METTL14, METTL16, WTAP, VIRMA, ZC3H13, RBM15, and RBM15B), readers (YTHDC1, YTHDC2, YTHDF1, YTHDF2, YTHDF3, HNRNPC, FMR1, LRPPRC, HNRNPA2B1, IGFBP1, IGFBP2, IGFBP3, and RBMX), and erasers (FTO and ALKBH5). The expression matrices of 23 m6A-related genes were extracted from the total matrix using R4.0.3 software.

### Identification of m6A-Related LncRNAs

From the GENCODE website (https://www.gencodegenes.org/human/release_29.html), Genome annotation files of Genome Reference Consortium Human Build 38 (GRCh38) were downloaded to distinguish lncRNAs from mRNAs in total matrix. m6A-related lncRNAs were screened using the Pearson’s correlation analysis (|Pearson R| > 0.5, *p* < 0.001). The co-expression network was plotted using the “Cytoscape” software (14). Univariate Cox regression analysis was conducted to identify m6A-related lncRNAs associated with OS, and these lncRNAs were identified as m6A-related prognostic lncRNAs.

### m6A-Related Risk Prognostic Signature Model Development

All samples were randomly separated into training and testing sets. To construct a m6A-LPS, we implemented the least absolute shrinkage and selection operator (LASSO) Cox regression analysis *via* “glmnet” R package in the training set, aiming at dimension reduction to select the most important m6A-related lncRNAs (15, 16). The tuning parameter was selected *via* ten-fold cross-validation to avoid overfitting of the predicted signal (17). The risk score for each patient was calculated using the following formula:


$$Risk\;score=\Sigma_{i=1}^{n}\;Coef_{i}*x_{i}$$

where *Coef_i_* is the LASSO Cox regression coefficient for the target m6A-related lncRNAs and *x_i_* is the expression (FPKM) value of each lncRNA. According to the median risk score in the training set, all BRCA patient samples were then divided into high-risk and low-risk groups.

### Validation of the Risk-Scoring Model and Characteristics Identification of Risk Subtype

To verify the predictive function and value of the model, the Kaplan–Meier log-rank test, time-dependent receiver operating characteristic (ROC) curve analysis, univariate Cox regression analysis, and multivariate Cox regression analysis were performed to compare survival between the high-risk and low-risk groups in the training, testing, and total cohorts. In this part, the “survival”, “timeROC”, and “survivalROC” packages for R were used for analysis, while the “survminer” and “pheatmap” packages for R were used for drawing plots. Next, we implemented principal component analysis (PCA) using R software for both three datasets and using the “ggplot2” package to plot scatter diagrams.

In each subtype with different clinicopathological characteristics, we applied our model to separate the samples into the high- and low-risk groups to conduct stratification analysis which compared survival between the two risk groups in corresponding clinicopathological subtypes. In the total cohort, the expression of the 10 m6A-related lncRNAs in the model was compared between the high- and low-risk groups. The risk score was compared between subgroups of clinicopathological characteristics, genotypes of m6A-related lncRNAs, tumor mutation burden (TMB) conditions, and TP53 gene mutation status using the Wilcoxon test. The TMB for each sample was calculated using the Perl language. The waterfall grams about the mutation status of top 20 mutational genes and m6A regulators were plotted using the “GenVisR” R package (18). The response of clinical common chemotherapeutics were predicted by the “pRRophetic” R package for risk groups (19).

### m6A-Related LncRNAs Genotyping Analysis and Characterization

Based on the expression of m6A-related prognostic lncRNAs, a dimensional reduction of lncRNA genotyping was performed by K-means clustering analysis using the “ConsensusClusterPlus” R package (16, 20). We divided *n* samples into *k* clusters in which each sample belonged to the cluster with the most similar mean. To determine the construct validity of the genotyping clusters, we employed the Kaplan–Meier log-rank test to compare survival between the clusters. The expression of m6A regulators, tumor microenvironment scores, and TMB were compared using the Kruskal test. The correlations between the different clusters and clinicopathological characteristics were also compared.

### Immune Characteristics Analysis of m6A-Related LncRNAs

The expression of human leukocyte antigen (HLA) genes was compared between the different genotypes. The data on the proportions of 22 tumor-infiltrating immune cells in each sample were calculated using xCell, TIMER, quanTiseq, MCP-counter, EPIC, CIBERSORT-ABS, and CIBERSORT for TCGA BRCA samples downloaded from the TIMER 2.0 database website (TIMER (shinyapps.io)). We then analyzed the correlation between tumor-infiltrating immune cells and the expression of the m6A-related lncRNAs and risk score using Spearman’s correlation analysis. We normalized the gene expression files of each BRCA sample to predict the proportions of 22 tumor-infiltrating immune cells in each sample *via* the “CIBERSORT” R package and selected significant results for further analysis. The Kruskal test was used to analyze differences in tumor-infiltrating immune cells between the three genotypes. The difference expression levels of immune check point genes between risk groups were analyzed using the “limma” R package.

### Functional and Pathway Enrichment Analysis

Differentially expressed genes (DEGs) between clusters were screened using the “limma” R package with |log2(Fold change)| > 0.5 and *p* < 0.05 and gene ontology (GO) term functional enrichment analysis were implemented. Gene set enrichment analysis (GSEA) was performed using “org.Hs.eg.db” R package (21) to explore the biological characterization of different genotypes and the low- and high-risk groups were analyzed similarly.

The target miRNAs of the 13 m6A-related prognostic lncRNAs were predicted using the miRcod database (http://www.mircode.org/) using the Prel programming language. Then, the shared target mRNAs of these miRNAs were screened in miRTarBase, miRD8, and TargetScan databases. DEGs were screened (with |log2(Fold change)| > 0.5 and *p* < 0.05) using the “limma” R package between BRCA and normal breast tissue (22) to determine the intersection between the DEGs and target mRNAs. We used “Cytoscape” to plot the ceRNA network. To predict the molecular mechanism of m6A-related lncRNAs, all the mRNAs in the intersection were subjected to functional and pathway enrichment analysis, including GO and Kyoto Encyclopedia of Genes and Genomes (KEGG) pathway analyses using R software, and the loop graph was drawn using the Sangerbox tools (http://www.sangerbox.com/tool).

### Quantitative Real-Time Polymerase Chain Reaction for Clinical Samples

In total, 72 BRCA and normal breast samples were collected from patients who underwent surgical treatment at the Department of Breast and Thyroid Surgery, the Third Xiangya Hospital of Central South University from 2019 to 2021. Forty-eight of the 72 samples included 24 luminal BRCAs, 22 Her-2 positive BRCAs, and 2 triple-negative BRCAs (TNBC). The 24 normal breast tissue samples were obtained from BRCA patients who underwent mastectomy. All the fresh samples were stored in liquid nitrogen. This study was approved by the Medical Ethics Committee of the Third Xiangya Hospital of Central South University. Sample acquisition and usage were implemented in accordance with approved guidelines. Each patient signed an informed consent form.

To validate the expression levels of m6A-related lncRNAs in clinical samples, we extracted total RNA from BRCA and normal breast tissue using RNA TRIzol reagent (Shanghai YEASEN Co., Ltd). cDNA of each sample was obtained using a reverse transcription kit (Guangzhou Ribobio Co., Ltd) according to the manufacturer’s instructions. The LightCycler 480 Real-Time PCR System was used for quantitative real-time polymerase chain reaction (qRT-PCR) analysis. GAPDH mRNA expression was used as an endogenous control, and the 2^-ΔΔCT^ formulation was used to calculate related lncRNA expression levels. The primer sequences of the corresponding lncRNA involved in our study are shown in **Table 1**.

**Table 1:** The quantitative real-time polymerase chain reaction primer sequence.

Genes	Sequence (5’→3’)
LRRC8C-DT	Forward 5’-AATCCTCTCTCCGCTTCACG-3
	Reverse 5’-GTTTTCCGCGCATTGTGAG-3’
OTUD6B-AS1	Forward 5’- AGCCGAGTCAGCCATAAAGCTA-3’
	Reverse 5’- AGATTAAAGAGGTCCTCTGAAGCAG-3’
ZNF197-AS1	Forward 5’-GTCATAGTGGCACAATCATAGCTC-3’
	Reverse 5’- CAGTGAATCAACACATAGAACCCTC-3’
COL4A2-AS1	Forward 5’- TGGAATCACAGAATCCGACCT-3’
	Reverse 5’- TGCTACCACCTAGATGACCCTT-3’

## Results

### Data Preparation and Identification of m6A-Related LncRNAs

**Figure 1:**
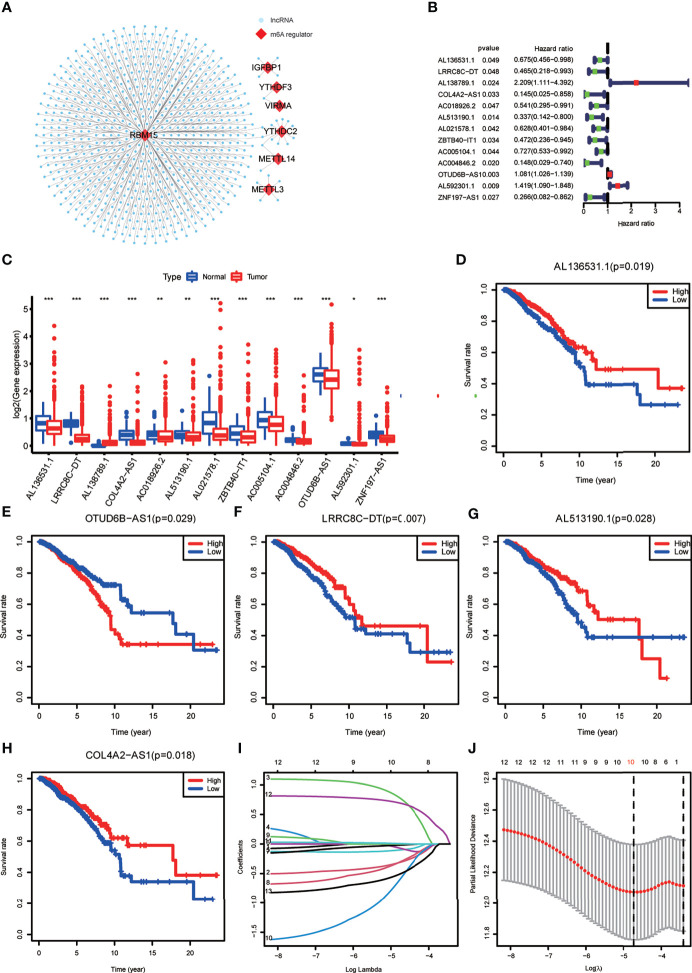
**(A)** The co-expression network of the N6-methyladenosine (m6A) regulators (red) and their target long non-coding RNAs (lncRNAs) (blue) (|Pearson R| >0.5, *p* < 0.001). **(B)** Forest plot of the prognostic ability of the 13 m6A-related lncRNAs. **(C)** The box plot reveals the expression level of the 13 prognostic m6A-related lncRNAs between breast cancer (BRCA) and normal breast tissue samples. *p < 0.05, **p < 0.01, and ***p < 0.001. **(D–H)** Kaplan–Meier curves showing that patients with different expression levels of the five of the 13 prognostic m6A-related lncRNAs had different overall survival. **(I, J)** Least absolute shrinkage and selection operator regression were performed and calculated the minimum criteria.

Expression data of 1022 BRCA samples and 112 normal samples were downloaded from TCGA, from which we obtained data for a total of 13,162 lncRNA expression profiles and 19,322 protein-coding gene expression profiles (**Table 2**). Next, the expression matrices of 23 m6A regulators were extracted from the total expression matrices. Based on the expression of m6A-related genes, 694 lncRNAs were identified as m6A-related lncRNAs (**Figure 1A**). Combined with the prognostic information, univariate Cox regression analysis was performed (*p* < 0.05) to obtain 13 m6A-related prognostic lncRNAs from the 694 lncRNAs (**Figure 1B**). Most lncRNAs were co-expressed with m6A writers, and no lncRNAs were corelated with m6A erasers. Compared with normal breast tissue, besides AL138789.1, BRCA tissue had lower expression levels of these lncRNAs (**Figure 1C**). To analyze the effect of the 13 lncRNAs on OS of BRCA, we performed the Kaplan– Meier survival analysis and the results showed that higher expression of LRRC8C-DT, AL51319.1, AL136531.1, and COL4A2-AS1 and lower expression of OTUD6B-AS1 were associated with better OS in the overall cohort of BRCA patients (**Figures 1D**–**H**). Other lncRNAs had no significant effect on OS of BRCA.

**Table 2:** Clinical baseline of 1022 breast cancer patients included in study from The Cancer Genome Atlas dataset.

Variables	Number (%)
Vital status	
Alive	882 (86.30)
Dead	140 (13.70)
Age	57.99 ± 12.90
≤ 35	32 (3.13)
≥ 35	990 (96.87)
Stage	
Stages I & II	756 (75.60)
Stages III & IV	244 (24.40)
AJCC-T	
T1	275 (26.99)
T2	581 (57.02)
T3	128 (12.56)
T4	35 (3.43)
AJCC-M	
M0	847 (97.69)
M1	20 (2.31)
AJCC-N	
N0	480 (47.76)
N1	345 (34.33)
N2	109 (10.85)
N3	71 (7.06)
Molecular type		
Luminal	349 (64.04)
Her-2 (+)	99 (18.17)
TNBRCA	97 (17.80)
TMB	
High	446 (48.69)
Low	470 (51.31)
BRCA1	
Wild-type	897 (97.93)
Mutated	19 (2.07)
BRCA2	
Wild-type	900 (98.25)
Mutated	16 (1.75)
TP53	
Wild-type	602 (65.72)
Mutated	314 (34.28)
PIK3CA	
Wild-type	615 (67.14)
Mutated	301 (32.86)

AJCC, American Joint Committee on Cancer; TMB, tumor mutation burden.

### Construction of the m6A-LPS Model

All the BRCA patient samples were randomly divided into the training and testing sets at a ratio of 7:3, which resulted in 718 samples in the training set (**Supplementary Table S1**) and 304 samples in the testing set (**Supplementary Table S2**). To construct the m6A-LPS model for predicting survival risk and OS of BRCA patients, we obtained a signature that contained 10 lncRNAs from the 13 m6A-related prognostic lncRNAs in the training set (**Figures 1I**, **J**). For each patient, a risk score was calculated based on the coefficient and expression of these 10 lncRNAs. Risk score = (-0.30*LRRC8C-DT) + (0.76*AL138789.1) + (-0.10*AC018926.2) + (-0.08*AL513190.1) + (-0.02*AL021578.1) + (-0.29*ZBTB40-IT1) + (-0.57AC004846.2) + (0.01*OTUD6B-AS1) + (0.69* AL592301.1) + (-0.50*ZNF197-AS1). Finally, the risk scores of all the BRCA patients we calculated ranged from 0.03 to 28.39, and the median value was 0.78.

### Validation of the m6A-LPS Model

**Figure 2:**
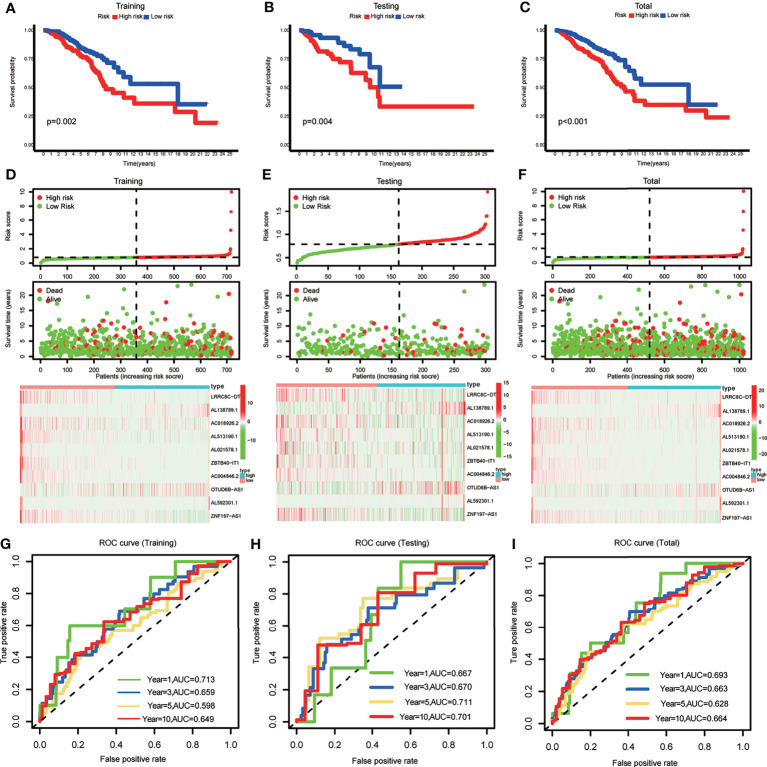
**(A–C)** Kaplan–Meier curves showing that the high-risk subgroup had worse overall survival than the low-risk subgroup in the training, testing and total cohort. **(D–F)** Distributions of risk scores and survival status, and the risk heatmap of m6A-related lncRNA prognostic signature (m6A-LPS) expression of BRCA patients in the training, testing and total cohort. **(G–I)** Time-dependent receiver operating characteristic (ROC) curves of m6A-LPS for predicting 1/3/5/10-year survival in the training, testing and total cohort.

BRCA patients were divided into low- and high-risk groups based on the median value of the risk scores in the training set. The BRCA patients in the high-risk group had lower OS rates and shorter OS indicated by Kaplan–Meier survival curves (**Figures 2A**–**C**). The risk score and survival status of each BRCA patient were represented by risk curves and scatter plots, respectively, in the training, testing, and total cohorts. Compared with the low-risk group, the high-risk group was associated with higher mortality. The risk heatmap showed that the expression of AL138789.1 and OTUD6B-AS1 increased with increasing risk score, whereas the expression of LRRC8C-DT, AC018926.2, AL51319.1, AL021578.1, ZBTB40-IT1, AC004846.2, AL592301.1, and ZNF197-AS1 decreased with increasing risk score (**Figures 2D**–**F**). The training, testing, and total cohort showed similar results.

The ROC curves demonstrated the promising predictive ability of the m6A-LPS model for BRCA patient survival in the training set (1-year AUC=0.713, 3-year AUC=0.659, 5-year AUC=0.598, and 10-year AUC=0.649), and similar trends were observed in the test set and overall cohort (**Figures 2G**–**I**). These results indicated that the m6A-LPS model had a stable and strong predictive ability for OS in BRCA patients.

**Figure 3:**
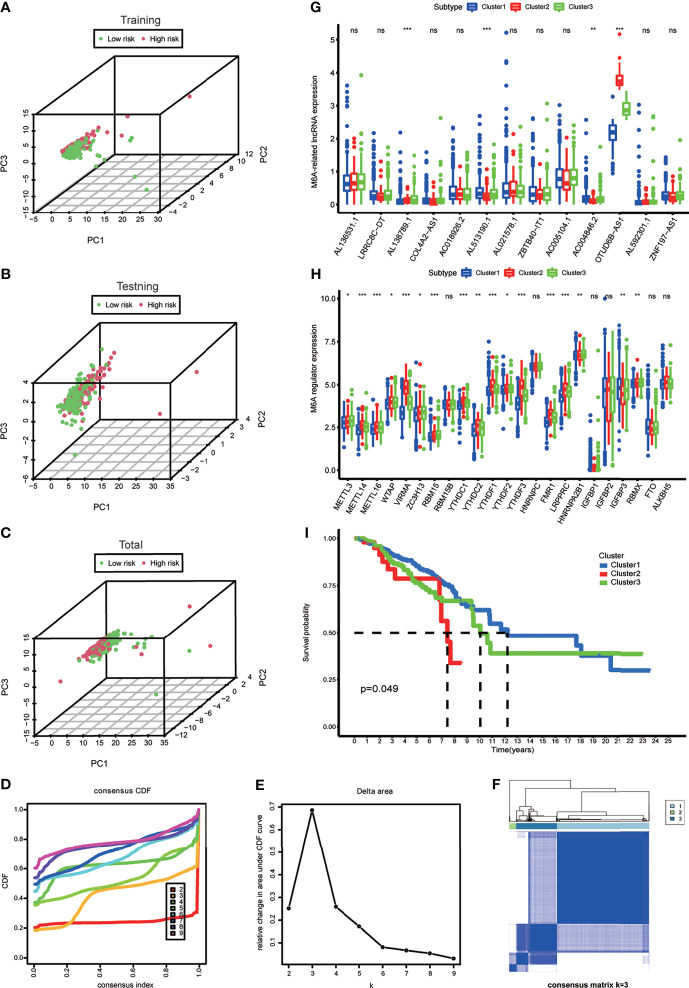
**(A–C)** Principal component analysis of the m6A-LPS expression in the training, testing and total cohort. **(D)** Consensus clustering cumulative distribution function (CDF) for k = 2 to 9. **(E)** The relative change in area under the CDF curve for k = 2 to 9. **(F)** Consensus matrix heatmap plots when k = 3. **(G)** The box plot showing the expression level of the 13 prognostic m6A-related lncRNAs between three genotypes. **(H)** The box plot showing the expression level of 23 m6A regulators between three genotypes. *p < 0.05, **p < 0.01, and ***p < 0.001. ns, no significance. **(I)** Kaplan–Meier curves showing that the Cluster 2 had worse overall survival than the other two clusters.

A diacritical pattern between the low- and high-risk groups was demonstrated by PCA in the three cohorts. There may be a difference in the m6A modification status of lncRNAs between the risk subgroups (**Figures 3A–C**).

### Identification and Comparison of Gene Expression of the m6A-Related LncRNAs Genotype

To explore the m6A modification patterns for lncRNAs, K-means clustering analysis was performed to obtain appropriate grouping according to the expression of 13 m6A-related prognostic lncRNAs. The total cohort was stratified into three groups: Cluster 1 (n = 675), Cluster 2 (n = 50), and Cluster 3 (n = 297) (**Figures 3D**–**F**). The results of correlation analysis between clinical characteristic and genotypes shown in the heatmap which helped to realize the traits of the populational constitution in each cluster at the same time (**Supplementary Figure S1**). The box plot showed differential expression levels of AL138789.1, AL51319.1, AC004846.2, and OTUD6B-AS1 (m6A-related prognostic lncRNAs) between the two genotypes (**Figure 3G**), and except for RBM15B, HNRNPC, IGFBP1, IGFBP2, FTO, and ALKBH5, the expression of the other m6A regulators showed significant difference (**Figure 3H**). This indicated that there was a diversity of m6A modification patterns for lncRNAs in BRCA. Kaplan–Meier survival curves showed that BRCA patients belonging to Cluster 2 had worse survival (**Figure 3I**).

### The Correlation of m6A-LPS Model and Clinicopathological Features

**Figure 4:**
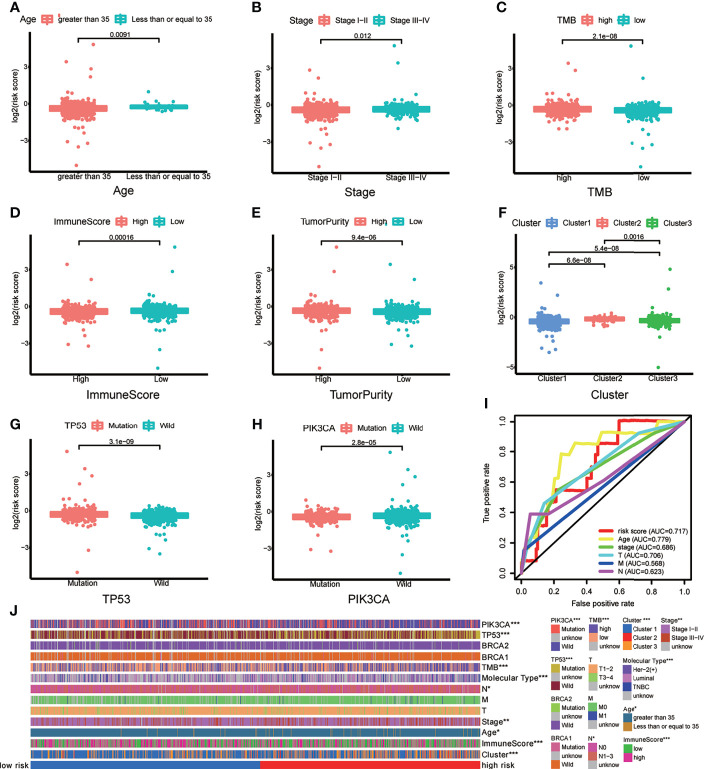
**(A–H)** Patients with different clinicopathological features (including age, American Joint Committee on Cancer [AJCC] stage level, tumor mutation burden (TMB), immune score, tumor purity, m6A-related lncRNAs genotypes, TP53 mutation status, and PIK3CA mutation status) had different levels of risk scores, calculated based on the m6A-related lncRNA prognostic signature (m6A-LPS). **(I)** Time-dependent ROC curves for the risk score, age and AJCC stage level and T/M/N stage. **(J)** Heatmap of the correlations between the risk score and clinicopathological features. *p < 0.05, **p < 0.01, and ***p < 0.001.

**Figure 5:**
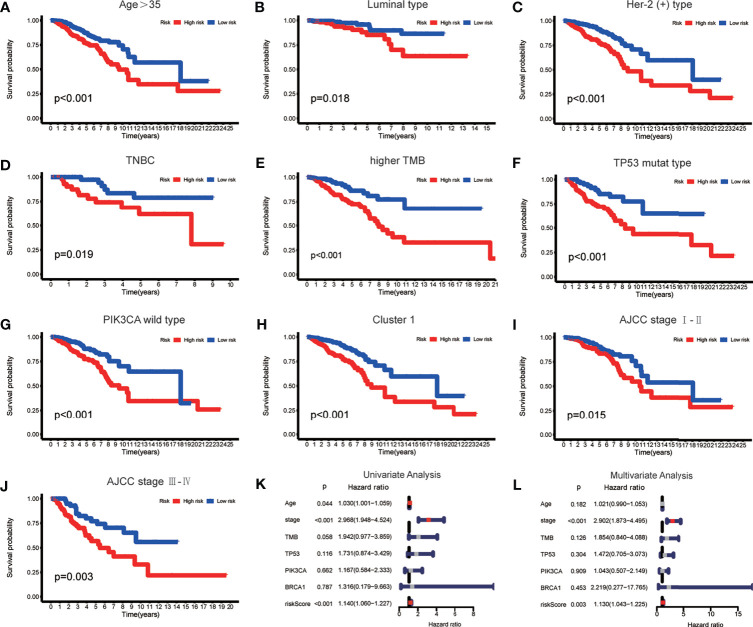
**(A–J)** The m6A-LPS retained its ability to predict overall survival (OS) in multiple subgroups of BRCA patients (including patients aged > 35 years, patients with luminal type, Her-2 positive type and TNBRCA, patients with higher TMB, patients with mutant type TP53 and wild-type PIK3CA, patients with Cluster 1, and patients with a higher or lower AJCC stage level). **(K, L)** Univariate and multivariate analyses demonstrated that risk score based on the m6A-LPS was an independent prognostic predictor.

To verify whether there is a correlation between clinicopathological features and risk scores associated with the m6A-LPS model, we performed the Wilcoxon test and Kruskal–Wallis test, and the results showed that BRCA patients with younger age, higher American Joint Committee on Cancer (AJCC) (23) stage level and TMB, lower immune score and tumor purity, mutation type of TP53, wild-type PIK3CA, and Cluster 2 of m6A-related lncRNA genotypic subgroup had higher risk scores (**Figures 4A**–**H**), whereas the mutation status of BRCA1/2 did not correlate with the risk score (**Figure 4J**). Compared with other clinicopathological features, the results of the multi-index ROC curve further supported the strong predictive ability of our model (AUC = 0.717) (**Figure 4I**). To further evaluate the prognostic value of the m6A-LPS model, stratification analysis was performed to verify whether the model retained its ability to forecast OS in various clinicopathological subgroups. Next, we confirmed that the m6A-LPS model retained its ability to predict OS for BRCA patients with age older than 35 years, luminal BRCA type, Her-2 positive BRCA type, TNBRCA, higher TMB, TP53 mutant type and PIK3CA wild-type, Cluster 1 of m6A-related lncRNAs genotype, as well as both patients with lower and higher AJCC TMN stage (**Figures 5A**–**J**). To make the Figure in the correct sequence, we moved this text to the front. These results demonstrated that our model is a stable and potentially predictive tool for patients with BRCA.

### Assessment of the m6A-LPS Model as an Independent Prognostic Factor for BRCA Patients

Based on the results of univariate and multivariate Cox regression analyses, we confirmed that the m6A-LPS model could be regarded as an independent prognostic factor for BRCA patients. In contrast, TMB and mutations in TP53, PIK3CA, and BRCA 1 cannot serve as independent prognostic factors for BRCA patients. The higher risk score, younger age, and higher AJCC stage level were significantly associated with worse survival in univariate Cox regression. The statistical significance of risk score and AJCC stage level were further verified in multivariate Cox regression; however, age was excluded (**Figures 5K, L**). The m6A-LPS model should serve as a reliable independent prognostic factor for OS in BRCA patients.

### Somatic Mutation in Breast Cancer and TMB Value Estimation

**Figure 6:**
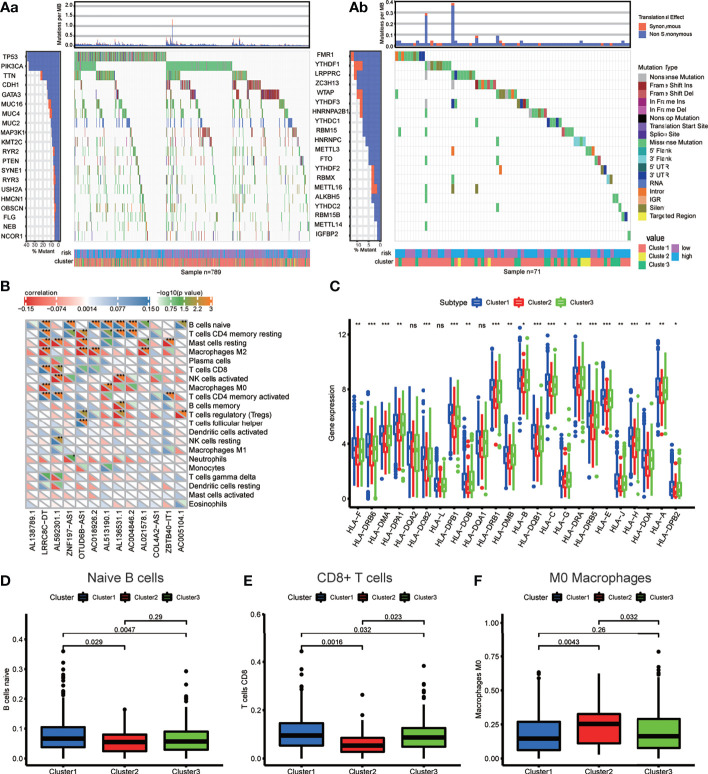
**(A)** Waterfall plot displays the frequently mutated genes in BRCA from The Cancer Genome Atlas dataset. The left panel shows the genes ordered by their mutation frequencies (including **(a)** the genes with top 20 mutation rate and **(b)** m6A regulators). The right panel presents different mutation types. **(B)** The heatmap demonstrated the correlations between each dysregulated immune microenvironment infiltration cell type and each m6A-related lncRNAs. **(C)** The box plot showing the expression level of HLA genes between three genotypes. **(D–F)** The box plot displays immunocytes (including naive B cells, CD8^+^ T cell and MO macrophages) with significant difference of infiltration level between the three genotypes. *p < 0.05, **p < 0.01, ***p < 0.001, and ****p < 0.0001. ns, no significance.

First, we extracted the variation in each TCGA BRCA sample to obtain the mutation status of each gene in 872 (88.4%) out of 986 samples. The waterfall diagram revealed that missense mutations, frameshift inserts, and frameshift deletions were common, and we integrated data of m6A-related lncRNA genotypic and risk subgroups to display the somatic mutation status of the top 20 higher mutation rate genes in 789 BRCA samples of the intersection (**Figure 6Aa**). The somatic mutation status of the m6A genes is shown in the waterfall diagram; similarly, ZC3H13, LRPPRC, YTHDF1, and FMR1 had mutation rates > 10% (**Figure 6Ab**).

### Immune-Related Characteristic Analysis

To investigate the immune characteristics mediated by m6A-related lncRNAs, we found that m6A-related lncRNAs were closely associated with many immune cells, according to the results of the correlation analysis (**Figure 6B**); for example, the expression levels of LRRC8C−DT were positively correlated with M0 and M2 macrophage abundance but negatively correlated with CD8^+^ T cell abundance, signifying that the increasing infiltration of CD8^+^ T cells in the BRCA tissue with a lower expression level of LRRC8C−DT than that in the normal breast tissue.

The different expression of HLA genes may reflect coincident immune microenvironment characteristics in m6A-related lncRNA genotypes: Cluster 1 had the highest HLA gene expression, Cluster 2 had the lowest HLA gene expression, and Cluster 3 was in the middle (**Figure 6C**). The Kruskal test was performed to compare the tumor-infiltrating immune cells between the clusters; we found a higher abundance of naive B cells and CD8^+^ T cells in Cluster 1, and a higher abundance of M0 macrophages in Cluster 2 (**Figures 6D**–**F**).

**Figure 7:**
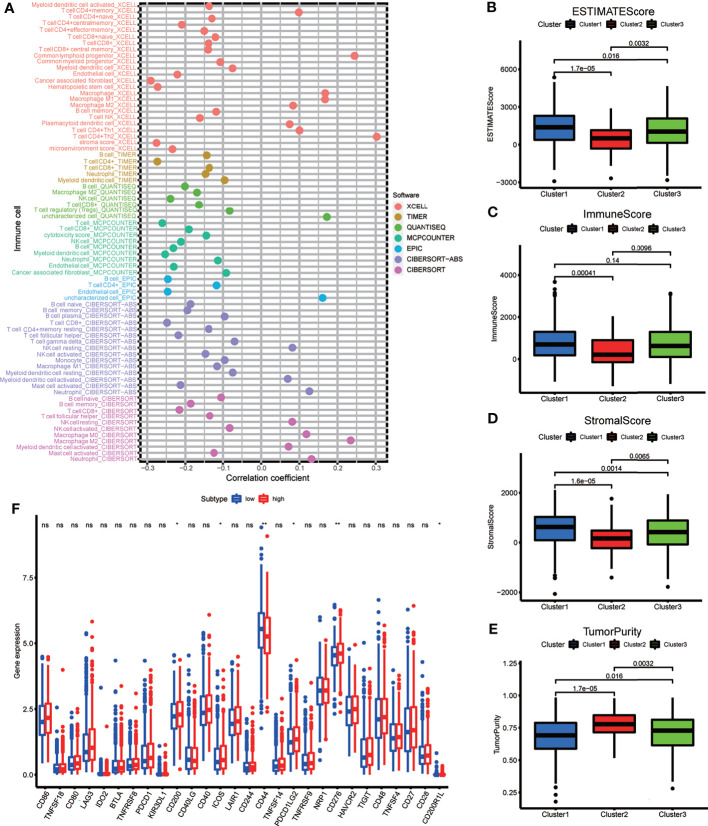
**(A)** The bubble plot demonstrated the correlations between each dysregulated immune microenvironment infiltration cell type and risk score based on m6A-LPS. **(B–E)** The box plots display the difference of immune microenvironment score (including immune score, stomal score and tumor purity) between the three genotypes. **(F)** The box plot showing the different expression level of immune check point genes between the risk subgroups. *p < 0.05, **p < 0.01, ***p < 0.001, and ****p < 0.0001. ns, no significance.

Spearman’s correlation analysis was employed to analyze the relevance between the risk score of BRCA patients and the abundance of tumor-infiltrating immune cells, which was calculated using seven software programs, and the statistically significant results (*p <* 0.05) are displayed in the bubble diagram (**Figure 7A**). We found that the abundance of CD4^+^ and CD8^+^ T cells was negatively correlated with the risk score for most software. The tumor microenvironment score was calculated based on normalized gene expression files of each sample and was compared using the Kruskal test. Based on the results, we found that Cluster 2 had lower immune and stromal scores but a higher tumor purity; in contrast, Cluster 1 had higher immune and stromal scores but a lower tumor purity (**Figures 7B**–**E**). The immune checkpoint genes, including CD200, ICOS, PDCD1LG2, and CD276, had higher expression in the high-risk group, and the expression of CD44 was higher in the low-risk group (**Figure 7F**). The above results indicate that each group has special immune characteristics and confirm that the modification of m6A methylation lncRNAs plays an indispensable role in the formation of the tumor immune microenvironment in BRCA.

### Drug Sensitivity Assessment

**Figure 8:**
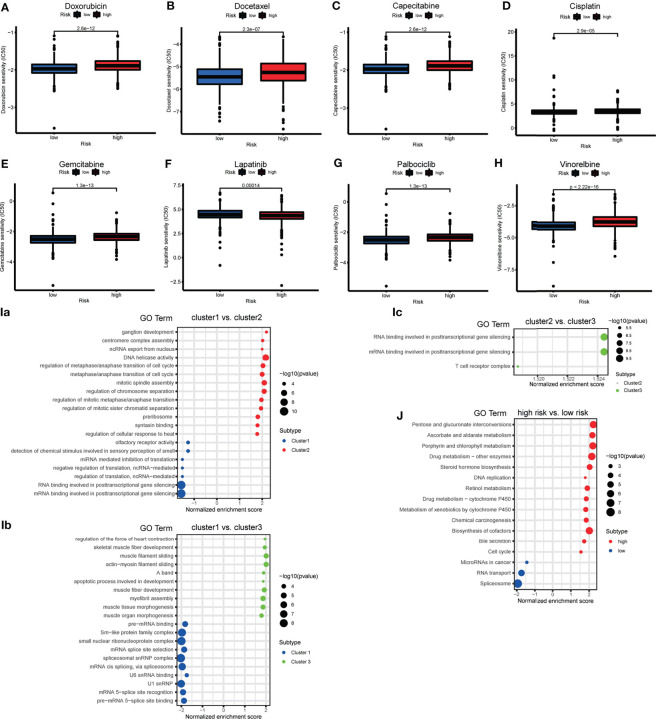
**(A–H)** The box plot showing the difference of drug half maximal inhibitory concentration (IC50) (including doxorubicin, docetaxel, capecitabine, cisplatin, gemcitabine, lapatinib, vinorelbine and palbociclib) between risk subgroups. **(I)** The bubble plot demonstrated the underlying biological function characteristics diversity among the three m6A-related lncRNA genotypes. **(a)** The differences of gene ontology (GO) term enrichment score between Cluster 1 and Cluster 2. **(b)** The differences of GO term enrichment scores between Cluster 1 and Cluster 3. **(c)** The differences of GO term enrichment score between Cluster 2 and Cluster 3. **(J)** The bubble plot demonstrated the underlying biological function characteristics diversity among risk subgroups that the differences of Kyoto Encyclopedia of Genes and Genomes (KEGG) pathway enrichment score between high- and low-risk groups.

To determine the appropriate chemotherapy or other agents for BRCA patients, we performed a drug sensitivity analysis to compare the half maximal inhibitory concentration (IC50) between risk groups. We found that, except for lapatinib, the low-risk group was more sensitive to doxorubicin, docetaxel, capecitabine, cisplatin, gemcitabine, vinorelbine, and palbociclib (**Figures 8A**–**H**). We suspect that the effect of tyrosine kinase inhibitors (TKIs) is superior in high-risk groups.

### GSEA

GSEA for the m6A**-**related lncRNA genotypes was performed to exploit the potential biological processes and pathways leading to molecular heterogeneity between m6A**-**related lncRNA genotyping. The GO results of comparison in pairs revealed molecular heterogeneity between the 3 m6A**-**related lncRNA genotyping. We found that many translation-related biological processes (BPs) were enriched in Cluster 1, cell cycle-and mitosis-related BPs were enriched in Cluster 2, and muscle fiber and apoptosis-related BPs were enriched in Cluster 3 (**Figures 8Ia–c**).

We similarly implemented GSEA for the different risk groups to show that KEGG pathways involving DNA replication, cell cycle, drug metabolism, were significantly enriched in the high-risk group. MiRNAs in cancer and RNA transport were enriched in the low-risk group (**Figure 8J**). This may provide some insights into the potential function of m6A-related lncRNAs.

### Differential Analysis for m6A-Related LncRNA Genotypes and Functional Enrichment Analysis

**Figure 9:**
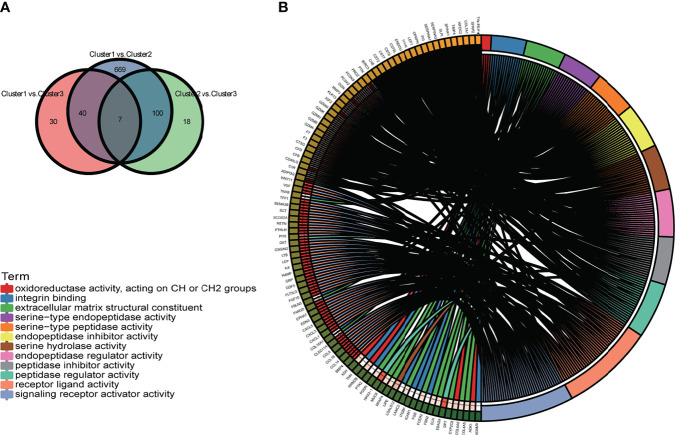
**(A)** The Venn diagram displays the differentially expressed genes (DEGs) of the three m6A-related lncRNA genotypes. **(B)** The circle plot demonstrates the enriched GO terms of the DEGs for the m6A-related lncRNAs genotypes. *p < 0.05, **p < 0.01, ***p < 0.001, and ****p < 0.0001.

To further define the differences in biological characteristics between m6A-related lncRNA genotypes, DEGs in the intersection of three genotypes compared in pairs were excluded, resulting in 717 DEGs (**Figure 9A**). We discovered that these DEGs were enriched in GO-BPs, such as signaling receptor activator activity, receptor–ligand activity, and some forms of enzyme activity (**Figure 9B**). This provided further insight into the differences between these genotypes.

### Construction of the ceRNA Network Based on m6A-Related Prognostic LncRNAs and Functional Enrichment Analysis

**Figure 10:**
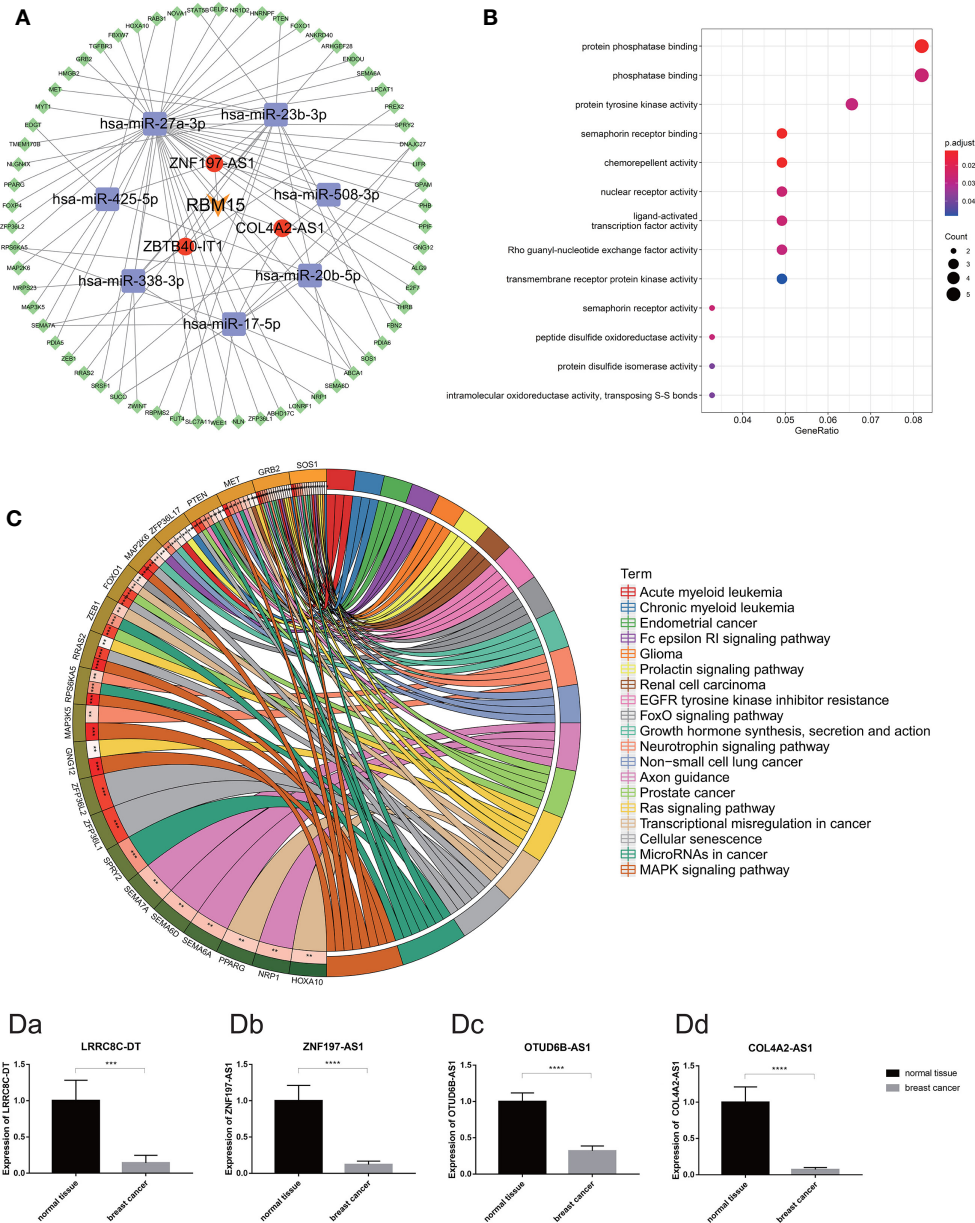
**(A)** The competing endogenous RNA network of one m6A regulates (orange) and three m6A-related lncRNAs (red) and their target miRNAs (purple) and mRNAs (green). **(B)** The bubble plot demonstrated the target mRNAs underlying biological function characteristics that enrich in the GO term. **(C)** The circle plot demonstrates the KEGG pathways involving the target mRNAs. **[D (a–d)]** Expression of m6A-related prognostic lncRNAs (including LRRC8C-DT, ZNF197-AS1, OTUD6B-AS1 and COL4A2-AS1) in breast cancer tissue and normal breast tissue carried out by quantitative real-time polymerase chain reaction. *p < 0.05, **p < 0.01, ***p < 0.001, and ****p < 0.0001.

To explore the potential function of m6A-related prognostic lncRNAs and how they regulate mRNA expression by sponging miRNAs to participate in BRCA progression, we constructed a ceRNA network based on the 13 m6A-related prognostic lncRNAs that were screened by univariate Cox regression analysis. Three of the 13 lncRNAs from the miRcode database had 48 pairs of interactions with the seven interactive miRNAs. All three lncRNAs were co-expressed with the m6A writer RBM15. In total, 538 target mRNAs of the seven miRNAs were identified from the miRTarBase, miRDB, and TargetScan databases simultaneously. Taking the intersection of all the target mRNAs and the DEGs between BRCA and normal breast tissue together, we obtained 62 target mRNAs to construct the ceRNA network. Finally, our ceRNA network included three lncRNAs, seven miRNAs, and 62 mRNAs (**Figure 10A**).

Moreover, functional enrichment analysis, including GO term analysis and KEGG pathway analysis of the 62 target mRNAs, was performed. We found that these genes were enriched in chemorepellent activity, protein phosphatase binding, nuclear receptor activity, and protein tyrosine kinase activity (GO Biological Processes), mitogen-activated protein kinase (MAPK) signaling pathway, cellular senescence, miRNAs in cancer, transcriptional dysregulation in cancer, Ras signaling pathway, etc. (KEGG pathway). All these data could guide us in new directions for further studies to determine the potential functions of these m6A-related lncRNAs in BRCA. In addition, KEGG pathway analysis revealed that these target genes were associated with multiple malignant tumors including prostate cancer, renal cell carcinoma, non-small cell lung cancer, endometrial cancer, acute myeloid leukemia, and glioma (**Figures 10B, C**).

### Validation of the Expression Level of Four m6A-Related Prognostic LncRNA in BRCA Clinical Samples

According to the difference analysis, the expression levels of m6A-related lncRNAs between BRCA and normal breast tissue were significantly different. To verify this result, we performed RT-qPCR assay to detect four of the 13 m6A-related prognostic lncRNA expression levels in the 72 clinical samples we collected, including 48 BRCA samples and 24 normal breast samples. The results of RT-qPCR in accordance with the difference analysis showed that LRRC8C-DT, ZNF197-AS1, OTUD6B-AS1, COL4A2-AS1 was downregulated in BRCA samples and upregulated in normal breast samples (**Figures 10Da–d**).

## Discussion

Recently, m6A modifications have been reported to not only affect cleavage, transport, stability, and degradation processes of non-coding RNAs, including lncRNAs, but they may also regulate biological cell functions by aberrant expression of lncRNAs (13). These processes may also be involved in diseases such as cancer. In the present study, we screened 13 m6A-related prognostic lncRNAs associated with BRCA including AL136531.1, LRRC8C-DT, AL138789.1, COL4A2-AS1, AC018926.2, AL513190.1, AL021578.1, ZBTB40-IT1, AC005104.1, AC004846.2, OTUD6B-AS1, AL592301.1, and ZNF197-AS1. Moreover, we explored the functional significance of these lncRNAs. The expression of each of these lncRNAs was significantly correlated with the survival of BRCA patients respectively demonstrated by the univariate Cox regression analysis. Especially, there was further proof of LRRC8C-DT, AL51319.1, AL136531.1, COL4A2-AS1, and OTUD6B-AS1 being correlated with survival. Among these lncRNAs, most were reported to serve as markers in our study for the first time. The lncRNA ZBTB40-IT1 was previously validated to modulate osteoporosis GWAS risk SNPs (rs34920465 and rs6426749) and plays a critical role in bone metabolism that suppresses osteogenesis (24). COL4A2-AS1 can serve as a biomarker in BRCA, and its high expression is related to poor prognosis (25). According to previous reports, OTUD6B-AS1 participates in different mechanisms in multiple tumors, such as inhibiting clear cell renal cell carcinoma proliferation *via* the Wnt/beta-catenin signaling pathway and targeting corresponding miRNAs to act on thyroid carcinoma, bladder cancer, hepatocellular carcinoma, and colorectal carcinoma (24, 26–28).

Furthermore, we identified a novel molecular signature comprising 10 lncRNAs based on 13 m6A-related prognostic lncRNAs through Cox and LASSO regression analyses and validated them. Kaplan–Meier survival curves indicated a significant divergence in patients who were divided into high- and low-risk groups. Time-dependent ROC curves demonstrated that our m6A-LPS was optimal in different cohorts. Moreover, we used a series of analyses to reveal that m6A-LPS can be an independent predictive marker and demonstrated the reproducibility and reliability of m6A-LPS for BRCA prognosis. Unfortunately, we could not find an association between our risk model and the mutation of BRCA1/2. The reasons may be that the mutation rate of BRCA1 and BRCA2 in the general population is only 0.1–0.2% and 0.8–4.4% in all BRCA cases, respectively (29, 30). The results of the drug sensitivity analysis showed that the high-risk group was sensitive to lapatinib, which means that the effect of TKI is superior in high-risk groups. In addition, GSEA revealed that the low-risk group was significantly enriched in pathways such as the Janus kinase/signal transducer and activator of transcription signaling pathway, MAPK signaling pathway, Notch signaling pathway, and FC epsilon RI signaling pathway. We believe that m6A-LPS plays a crucial role in the molecular mechanisms of oncogenesis, progression, and prognosis of BRCA. These results confirm that m6A-LPS may provide a reliable prognostic marker and a theoretical basis for the mechanism of BRCA. Despite including training and testing sets to ensure the reliability of results, our study has several limitations. Additional *in vitro* and *in vivo* experiments are needed to further confirm the interaction between these lncRNAs and m6A-related genes and how these interactions affect the pathological progress in tumors, especially in BRCA.

In addition, we tried to identify the underlying m6A-related genotype of BRCA and obtained three subtypes (Cluster 1, Cluster 2, and Cluster 3) based on the expression of 13 m6A-related prognostic lncRNAs. To further explore the different biological characteristics between the genotypes and determine the correlation between these features and m6A-related lncRNAs, we performed a series of analyses and obtained abundance results. We found that most m6A regulators displayed a significantly higher expression status in Cluster 2 than in Clusters 1 and 3. In Cluster 2, the expression of OTUD6B−AS1 and AL138789.1 was higher, while that of AL513190.1 and AC004846.2 was lower. These results suggested that these four lncRNAs are closely correlated with m6A modification. We then assessed tumor-related features in the three clusters. We found that each cluster had unique immune characteristics. Cluster 2 showed a lower immune infiltration level except for M0 macrophages, but a higher tumor purity. The difference analysis of HLA genes showed the same trend: Cluster 2 had the lowest expression level of HLA genes. The test revealed that Cluster 2 exhibited a lower survival risk than other clusters, and the Kaplan–Meier log-rank test showed that Cluster 2 had a worse survival outcome. It is worth noting that there were just 50 patients in Cluster 2. But the correlation analysis between clinical characteristics and genotypes indicated that the BRCA patients with high TMB having a higher proportion in Cluster 2, which would reinforce the association results about the BRCA samples in Cluster 2 with a worse prognosis. Maybe we will amplify the sample size in future analyses to enhance the statistical strength and the reliability of the underlying m6A-related genotype. The role of m6A modification in immunity, especially in infiltrating immunocytes of the tumor microenvironment, has been identified and continues to be researched (31). It has been reported that a lower immune score, which represents a worse immune microenvironment, most likely leads to tumor immune escape with a lower survival rate and a higher recurrence rate (32). This is consistent with the results of our study. This indicates that the m6A-related lncRNA genotype identified in this study may be closely correlated with the immune microenvironment of BRCA. We found that many translation-related BPs were enriched in Cluster 1, cell cycle-and mitosis-related BPs were enriched in Cluster 2, and muscle fiber and apoptosis-related BPs were enriched in Cluster 3. DEGs of the three genotypes were enriched in GO-BPs, such as signaling receptor activator activity, receptor–ligand activity, and some kinds of enzyme activity. These results may point to a direction for further research.

Finally, we identified target miRNAs and mRNAs for three of the 13 m6A-related prognostic lncRNAs and performed enrichment analysis to explore the potential molecular functions and pathways of m6A-related lncRNAs. The target mRNAs were enriched in pathways such as Ras protein signal transduction and regulation of apoptotic signaling pathway, miRNAs in cancer, further confirming that the three lncRNAs play an important role in the mechanism of BRCA. The mechanism of m6A modification of lncRNAs or the interaction between m6A-related genes and lncRNAs is unclear. It has been reported that m6A modifications may modulate the function of lncRNAs by providing a binding site for the m6A reader proteins or by modulating the structure of the local RNA to induce RNA-binding protein entry, and might also regulate the relationship between lncRNAs and specific DNA sites by affecting the RNA-DNA triple helix structure (13). In the current study, the three m6A-related prognostic lncRNAs involved in the ceRNA network were correlated with RBM15. A recent study showed that XIST could regulate the transcriptional silencing of genes by forming the RNA-binding protein 15 (RBM15)/RBM15B-WTAP-METTL3 complex, which recruits the silencing complex. In addition, knocking down METTL3 or RBM15 reduced the level of m6A modifications on specific transcripts and this resulted in the inactivation of the lncRNA X chromosome (33). Therefore, we believe that the m6A regulator RBM15 plays an important role in lncRNA m6A modification, and the mechanism is worthy of further research.

In conclusion, we identified 13 target lncRNAs associated with BRCA survival; among these, 10 lncRNAs were used to build a prognostic model with m6A modification acting as a novel prognostic clinical trait to predict survival outcome for BRCA patients. Further, we discovered three genotypes relevant to m6A modification lncRNAs, which may provide new insights for precision treatment. Finally, we believe that our results can provide a theoretical basis for further research, and we plan to pursue further studies.

## Data Availability Statement

Publicly available datasets were analyzed in this study. This data can be found here: https://cancergenome.nih.gov/.

## Ethics Statement

The studies involving human participants were reviewed and approved by the Medical Ethics Committee of the Third Xiangya Hospital of Central South University. The patients/participants provided their written informed consent to participate in this study.

## Author Contributions

LQ constructed and supervised this study. BD did the project administration and funding acquisition. XZ performed the data analysis, figures plotted, and writing. XZ and JL did the polymerase chain reaction experiments. JL was responsible for the critical reading of the manuscript. XW performed the figure plotting. XRW and HL were responsible for the data acquisition. All authors read and approved the final manuscript.

## Funding

This work was supported by the Natural Science Foundation of Hunan Province, China (No. 2020JJ4831).

## Conflict of Interest

The authors declare that the research was conducted in the absence of any commercial or financial relationships that could be construed as a potential conflict of interest.

## Publisher’s Note

All claims expressed in this article are solely those of the authors and do not necessarily represent those of their affiliated organizations, or those of the publisher, the editors and the reviewers. Any product that may be evaluated in this article, or claim that may be made by its manufacturer, is not guaranteed or endorsed by the publisher.

## Supplementary Material

Supplementary Figure 1 Heatmap of the correlations between the genotypes of m6A-related prognostic lncRNAs and clinicopathological features or expression levels of m6A-related prognostic lncRNAs. *p < 0.05, **p < 0.01, and ***p < 0.001.

Supplementary Table 1 Survival message and expression levels of m6A-LPS of BRCA samples belong to training set from TCGA dataset.

Supplementary Table 2 Survival message and expression levels of m6A-LPS of BRCA samples belong to testing set from TCGA dataset.
