# Supplementary figures and images for: Identification of N6-Methyladenosine-Related LncRNAs for Predicting Overall Survival and Clustering of a Potentially Novel Molecular Subtype of Breast Cancer
Source: Front Oncol. 2021 Oct 15;11:742944. doi: 10.3389/fonc.2021.742944 (PMC8554333; doi:10.3389/fonc.2021.742944)

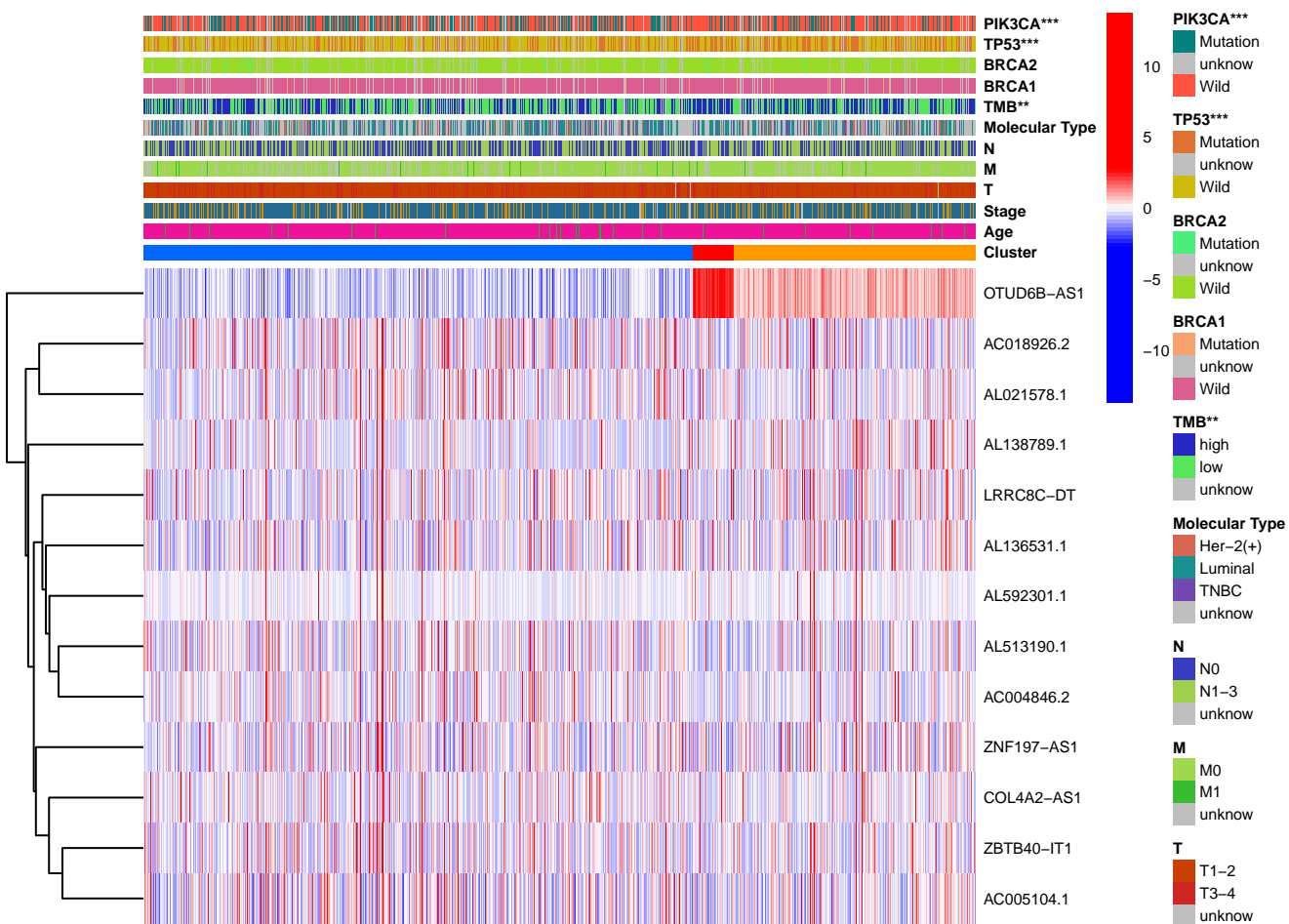

Supplement: Supplementary Figure 1 — Heatmap of the correlations between the genotypes of m6A-related prognostic lncRNAs and clinicopathological features or expression levels of m6A-related prognostic lncRNAs. *p < 0.05, **p < 0.01, and ***p < 0.001. [file Image_1.pdf]
