# Supplementary material for: Identification of N6-Methyladenosine-Related LncRNAs for Predicting Overall Survival and Clustering of a Potentially Novel Molecular Subtype of Breast Cancer
Source: Front Oncol. 2021 Oct 15;11:742944. doi: 10.3389/fonc.2021.742944 (PMC8554333; doi:10.3389/fonc.2021.742944)
Supplement: Supplementary Table 1 — Survival message and expression levels of m6A-LPS of BRCA samples belong to training set from TCGA dataset. [file Table_1.docx]

| id | futime | fustat | AL136531.1 | LRRC8C-DT | AL138789.1 | COL4A2-AS1 | AC018926.2 | AL513190.1 | AL021578.1 | ZBTB40-IT1 | AC005104.1 | AC004846.2 | OTUD6B-AS1 | AL592301.1 | ZNF197-AS1 |
| --- | --- | --- | --- | --- | --- | --- | --- | --- | --- | --- | --- | --- | --- | --- | --- |
| TCGA-A8-A06Q | 0.08493151 | 0 | 0.3073028 | 0.05434178 | 0.5082315 | 0 | 0.1594899 | 0.1872885 | 0.08001808 | 0.08312501 | 0.3850644 | 0.1168243 | 4.326007 | 0.1050608 | 0.1092767 |
| TCGA-A8-A09N | 0.08493151 | 0 | 0.2254618 | 0.1140266 | 0.04509235 | 0.04008209 | 0.1303875 | 0.6252144 | 0.2136958 | 0.2378498 | 0.685567 | 0 | 4.617745 | 0.1002052 | 0.1737105 |
| TCGA-E9-A1RD | 0.09315068 | 0 | 0.6387543 | 0.1527137 | 0.06387543 | 0.1589788 | 0.09850668 | 0.4723443 | 0.3459544 | 0.4043104 | 1.082125 | 0.1262711 | 2.945094 | 0.04258362 | 0.2362263 |
| TCGA-AC-A8OR | 0.10958904 | 0 | 0.9233564 | 0.9239807 | 0.05431508 | 0 | 0.2617594 | 0.1434456 | 0.1838592 | 0.1909981 | 0.6488318 | 0.1677686 | 3.423233 | 0 | 0.1255437 |
| TCGA-AC-A6NO | 0.13972603 | 0 | 1.092576 | 0.1209322 | 0.1213973 | 0.07193915 | 0.507041 | 0.8282403 | 0.2739573 | 0.6403375 | 1.625955 | 0.149989 | 3.193624 | 0.02248099 | 0.1558874 |
| TCGA-AN-A04D | 0.14246575 | 0 | 0.22531 | 0.1424374 | 0.2816375 | 0.1001378 | 0.3528952 | 0.1487604 | 0.7245513 | 0.1980747 | 0.4893611 | 0.1043907 | 4.688428 | 0.04693959 | 0.0650976 |
| TCGA-AN-A04C | 0.14794521 | 0 | 0.330186 | 0.08280526 | 0.03001691 | 0.05336339 | 0.3761154 | 0.1783673 | 0.3657914 | 0.05277698 | 1.043121 | 0.1483461 | 5.035287 | 0.05002818 | 0.115635 |
| TCGA-E9-A248 | 0.16164384 | 0 | 0.2258943 | 0.2522301 | 0.4517887 | 0 | 0.2488337 | 0.1917594 | 0.1747802 | 0.2269582 | 0.5957653 | 0.05980655 | 5.547389 | 0.08964061 | 0.0745902 |
| TCGA-OK-A5Q2 | 0.17534247 | 0 | 0.4860845 | 0.6145896 | 0.1215211 | 0.1080188 | 0.1561717 | 0.4279149 | 0.164542 | 0.07122118 | 1.099739 | 0.2502366 | 3.41691 | 0 | 0.1248371 |
| TCGA-AC-A8OS | 0.19178082 | 0 | 0.8235858 | 0.3786601 | 0.04117929 | 0.6222648 | 0.1190726 | 0.8156558 | 0.3624242 | 1.230853 | 3.175091 | 0.1271947 | 3.157833 | 0.09150953 | 0.09518166 |
| TCGA-BH-A0HO | 0.20821918 | 0 | 0.2824263 | 0.06492557 | 0.02567511 | 0.04564465 | 0.0494942 | 0.0508558 | 0.01738231 | 0.09028611 | 0.3624722 | 0.01586107 | 3.347122 | 0.0285279 | 0.1186907 |
| TCGA-AQ-A54N | 0.21369863 | 0 | 0.9083085 | 1.044033 | 0 | 0 | 0.1250683 | 0.3426906 | 0.2635431 | 0 | 0.4931992 | 0.2805586 | 4.10977 | 0.1802199 | 0.2499358 |
| TCGA-E9-A1R3 | 0.21369863 | 0 | 0.5003203 | 0.09310832 | 0 | 0.08471031 | 0.3444546 | 0.251684 | 0.2096849 | 0.293228 | 0.3363498 | 0.2207701 | 5.511457 | 0.01323599 | 0.1835617 |
| TCGA-BH-A0DS | 0.21369863 | 0 | 0.22090037 | 0.1320324 | 0 | 0.15708471 | 0.17033281 | 0.14584894 | 0 | 0 | 0.38382689 | 0 | 7.10410292 | 0 | 0.13615689 |
| TCGA-HN-A2NL | 0.21643836 | 0 | 0.8737225 | 1.900404 | 0.08401178 | 0.2688377 | 0.2267306 | 0.7987475 | 0.06825217 | 0.05908521 | 0.7663699 | 0.2075967 | 2.636032 | 0.05600785 | 0.3365865 |
| TCGA-LL-A6FQ | 0.21917808 | 0 | 0.310083 | 0.0891043 | 0 | 0.03445366 | 0.2241564 | 0.5374203 | 0.1574469 | 0 | 0.08418542 | 0.07183389 | 1.60692 | 0.02153354 | 0.1493176 |
| TCGA-AR-A24P | 0.23013699 | 0 | 0.6440995 | 0.1094422 | 0.2380368 | 0.07467821 | 0.215937 | 0.1664081 | 0.3981433 | 0.7385757 | 1.09483 | 0.1037998 | 3.986318 | 0.03111592 | 0.1294582 |
| TCGA-E9-A1R5 | 0.25205479 | 0 | 0.3652625 | 0.04198419 | 0.04565781 | 0.08116944 | 0.0586767 | 0.08038789 | 0.06182158 | 0.4281465 | 0.462776 | 0.05641119 | 5.18139 | 0.1014618 | 0.1172592 |
| TCGA-UU-A93S | 0.31780822 | 1 | 0.6548783 | 0 | 0.06548783 | 0 | 0.3787248 | 0.2594291 | 0.1773436 | 0 | 0.4978261 | 0.202279 | 2.92423 | 0.03638213 | 0 |
| TCGA-BH-A0E0 | 0.36712329 | 0 | 0.264509 | 0.1580973 | 0.2909599 | 0.2821429 | 0 | 0.244498 | 0.1790752 | 0.09301414 | 0.2872496 | 0.03268064 | 3.279224 | 0 | 0.203795 |
| TCGA-BH-A6R9 | 0.43835616 | 0 | 0.13402864 | 0.30040903 | 0 | 0.11913657 | 0.32296059 | 0.13273813 | 0.06805404 | 0.11782738 | 0.83692094 | 0.12419643 | 2.154629223 | 0.09307545 | 0.232345 |
| TCGA-AN-A0XV | 0.44383562 | 0 | 0.7557373 | 0.1816297 | 0.1545826 | 0.1221394 | 0.2648805 | 0.0907225 | 1.907028 | 0.7851816 | 0.7461002 | 0.0848845 | 8.031745 | 0.05725283 | 0.3970019 |
| TCGA-AN-A0XL | 0.44657534 | 0 | 0.560019 | 0.2918107 | 0.2053403 | 0.09955894 | 0.7197032 | 0.4190514 | 0.8088286 | 0.5251461 | 1.58123 | 0.2075746 | 5.573909 | 0 | 0.2013552 |
| TCGA-BH-A0RX | 0.46575342 | 0 | 0.5153891 | 0.2369605 | 0.06442364 | 0.03817697 | 0.289777 | 0.08507111 | 0.08723088 | 0.07551489 | 0.3264909 | 0.07959678 | 4.490893 | 0.07158182 | 0.3639987 |
| TCGA-AQ-A0Y5 | 0.47123288 | 1 | 0.4083495 | 0.1227576 | 0.01570575 | 0.05584267 | 0.211933 | 0.1451756 | 0.1701272 | 0.3313741 | 0.4434565 | 0.09702395 | 4.268075 | 0.01745084 | 0.2420148 |
| TCGA-AN-A0FF | 0.47123288 | 0 | 1.342279 | 0.179999 | 0.1864276 | 0.1325707 | 0.1437514 | 0.3692651 | 0.2776693 | 0.3277847 | 0.9717854 | 0 | 3.023049 | 0.02071418 | 0.287272 |
| TCGA-BH-A0HK | 0.48767123 | 0 | 0.3049427 | 0.130189 | 0 | 0.1161687 | 0.08397734 | 0.1150501 | 0.7373187 | 0.1531894 | 0.4257765 | 0.02691166 | 3.737348 | 0 | 0.4027677 |
| TCGA-E9-A1R4 | 0.50958904 | 0 | 0.85264953 | 0.26134852 | 0.22331297 | 0.03609098 | 0 | 0.42892175 | 0.19241738 | 0.35694381 | 0.74958201 | 0.25082538 | 7.08990563 | 0.20301179 | 0.03128272 |
| TCGA-EW-A1PC | 0.51232877 | 0 | 1.442157 | 0.03371494 | 0.329985 | 0.06518222 | 0.07067951 | 0.3066344 | 0.1820321 | 0.4297729 | 0.6105303 | 0.2265019 | 5.553017 | 0.08147777 | 0.4143204 |
| TCGA-AN-A0FD | 0.5369863 | 0 | 0.2456015 | 0.1882004 | 0.04093358 | 0 | 0.1183622 | 0.1621578 | 0.3048368 | 0.07197113 | 0.1333583 | 0.1264358 | 1.889934 | 0 | 0.2207654 |
| TCGA-E9-A1RF | 0.54794521 | 0 | 0.954079 | 0.1576424 | 0.1043524 | 0.1060088 | 0.2011612 | 0.4133905 | 0.5248108 | 0.2621096 | 1.003726 | 0.03683703 | 2.815081 | 0.09938323 | 0.04594281 |
| TCGA-AN-A0FS | 0.57534247 | 0 | 0.6753233 | 0.2299952 | 0.03751796 | 0.04446573 | 0.1687555 | 0.2311973 | 0.575734 | 0.3957939 | 0.7605451 | 0.1545141 | 4.671233 | 0.01389554 | 0.4046881 |
| TCGA-E9-A249 | 0.59452055 | 0 | 0.769937 | 0.07324014 | 0.119473 | 0.0707988 | 0.07676979 | 0.3330563 | 0.1437945 | 0.2800832 | 0.7784665 | 0.06560507 | 3.344475 | 0.04424925 | 0.2863772 |
| TCGA-AN-A0FN | 0.59726027 | 0 | 0.3531769 | 0.4194822 | 0.02943141 | 0.2616125 | 0.2836762 | 0.09716009 | 0.278955 | 0.4139803 | 0.8629662 | 0.03636313 | 3.376016 | 0.06540313 | 0.2721106 |
| TCGA-A2-A4RW | 0.60821918 | 0 | 1.69336825 | 0.31719116 | 0.09407601 | 0.27874375 | 0.30225225 | 0.45549872 | 0.33968208 | 0.55136125 | 1.90706128 | 0.15497722 | 3.231367824 | 0.06968594 | 0.19328619 |
| TCGA-AN-A0AK | 0.61369863 | 0 | 0.39663808 | 0.03039372 | 0 | 0.07834826 | 0 | 0.0290977 | 0.1790186 | 0 | 0.1914392 | 0.0816758 | 6.033950622 | 0 | 0.16977553 |
| TCGA-BH-A18T | 0.61369863 | 1 | 0.2678205 | 0.1504994 | 0.2529416 | 0.02645141 | 0.08604675 | 0.1964754 | 0.08058539 | 0.05232147 | 0.3877944 | 0.2022154 | 4.872975 | 0.09919278 | 0.2522012 |
| TCGA-GI-A2C8 | 0.61643836 | 0 | 0.5608882 | 0.3393153 | 0 | 0.1836827 | 0.3414412 | 0.05847238 | 0.8194106 | 0.3114243 | 1.122044 | 0.2006022 | 20.8786 | 0.03280048 | 0.1137224 |
| TCGA-LL-A73Z | 0.62191781 | 1 | 1.032589 | 0.3509048 | 0 | 0.2394409 | 0.6490868 | 0.3260612 | 0.06078894 | 0.07893656 | 0.4875493 | 0.2218757 | 6.896328 | 0.09976704 | 0.2767214 |
| TCGA-AN-A0AL | 0.62191781 | 0 | 0.18973997 | 0.13957883 | 0.018974 | 0.1349262 | 0.32918742 | 0.37582608 | 0.2312205 | 0.1334435 | 0.37089443 | 0.11721388 | 3.567831431 | 0.04216444 | 0.17542572 |
| TCGA-AN-A0FL | 0.63287671 | 0 | 0.3164353 | 0.05455782 | 0.01977721 | 0.07031896 | 0.07624948 | 0.2089257 | 0.5087959 | 0 | 0.04295502 | 0.2443516 | 5.674667 | 0.1318481 | 0 |
| TCGA-EW-A1P8 | 0.65479452 | 1 | 0.5006391 | 0.2548409 | 0.08939984 | 0 | 0.1378696 | 0.09444164 | 0.07262949 | 0 | 0.2718402 | 0.08836432 | 3.776306 | 0.05959989 | 0.0275518 |
| TCGA-AN-A0FJ | 0.6630137 | 0 | 0.30322416 | 1.04560053 | 0.06064483 | 0.10781303 | 0.05845285 | 0.16016242 | 0 | 0 | 0.3292932 | 0.22478393 | 4.345355037 | 0.03369157 | 0.14017425 |
| TCGA-A8-A07S | 0.66575342 | 0 | 0.4764974 | 0.05476982 | 0.1021066 | 0.03025381 | 0.06561066 | 0.1573031 | 0.2534663 | 0.4787415 | 0.6283483 | 0.1261549 | 3.137454 | 0.09454314 | 0.2360088 |
| TCGA-AC-A7VB | 0.68493151 | 0 | 0.1248701 | 0.06458796 | 0.2185226 | 0.0277489 | 0.8424968 | 0.5152822 | 1.098997 | 0.3842156 | 1.389956 | 0.07713981 | 3.341505 | 0.01734306 | 0.3848323 |
| TCGA-A1-A0SB | 0.70958904 | 0 | 0.2926112 | 0.3267251 | 0 | 0.2600988 | 0.0805814 | 0.3863917 | 1.075404 | 0.07349732 | 0.8171172 | 0.1291169 | 3.135433 | 0.1625618 | 0.2898602 |
| TCGA-A7-A6VY | 0.72876712 | 0 | 0.50952043 | 0.15617484 | 0.06793606 | 0.21135662 | 0.39288322 | 0.08970924 | 0.09198676 | 0.17917202 | 1.18042741 | 0.12590466 | 5.212388021 | 0.15096902 | 0.15702716 |
| TCGA-OL-A97C | 0.74246575 | 0 | 0.74223976 | 0.31282135 | 0.03092666 | 0.76973012 | 0.77502947 | 0.49006201 | 1.59126154 | 0.21750616 | 1.47776241 | 0.45852649 | 1.8379976 | 0 | 0.47655843 |
| TCGA-A8-A09R | 0.74794521 | 0 | 0.680188 | 0.07818253 | 0.04251175 | 0 | 0.05463357 | 0.03742437 | 0.1918725 | 0.1993225 | 0.3693328 | 0.1400644 | 4.167835 | 0.09447055 | 0.196523 |
| TCGA-A8-A07R | 0.74794521 | 0 | 0.2799832 | 0.2188375 | 0.1399916 | 0.0497748 | 0.4317814 | 0.0739433 | 0.09477571 | 0.2953669 | 0.3040542 | 0.08648131 | 2.536204 | 0.01555462 | 0.04314349 |
| TCGA-A8-A079 | 0.75068493 | 0 | 0.7781096 | 0.04293018 | 0.1556219 | 0.02766612 | 0.6299875 | 0.1232988 | 0.3582157 | 0.3283451 | 1.081608 | 0.05768225 | 6.832136 | 0 | 0.2158224 |
| TCGA-BH-AB28 | 0.78630137 | 0 | 0.64147772 | 0.16712829 | 0.17106072 | 0.03801349 | 0.74195013 | 0.31059169 | 0.20266715 | 0.07519153 | 1.20748746 | 0.07925593 | 3.176964021 | 0.04751687 | 0.16474548 |
| TCGA-E9-A2JT | 0.7890411 | 0 | 0.97678807 | 1.00183391 | 0 | 0.26715571 | 0 | 2.33164096 | 0.10173772 | 1.1889897 | 1.38715465 | 0.13925105 | 4.837612385 | 0.08348616 | 0.17367265 |
| TCGA-BH-A6R8 | 0.80273973 | 0 | 0.4556763 | 0.0886373 | 0.01752601 | 0 | 0.2364956 | 0.06942904 | 0.3322279 | 0.1232599 | 0.1522622 | 0.06496128 | 3.242356 | 0 | 0.08101912 |
| TCGA-BH-A8FY | 0.80821918 | 1 | 0.1606708 | 0.1108074 | 0.05355692 | 0.04760615 | 0.2064845 | 0.5304124 | 0.2175514 | 0.2824981 | 0.9887432 | 0.1654268 | 3.837489 | 0.02975385 | 0.2063188 |
| TCGA-C8-A278 | 0.81369863 | 0 | 0.123626 | 0.2913027 | 0.09271953 | 0.02747245 | 0.4170517 | 0.0408119 | 0.1883161 | 0.1086822 | 0.5034544 | 0.2291139 | 8.26807 | 0.03434057 | 0.1666868 |
| TCGA-E9-A1NI | 0.82191781 | 0 | 0.3883164 | 0.03347555 | 0.09707909 | 0.08629253 | 0.2339255 | 0.256385 | 0.2957558 | 0.341377 | 0.1581379 | 0.1499291 | 3.229831 | 0.08089925 | 0.1495922 |
| TCGA-D8-A1Y1 | 0.82739726 | 1 | 0.3304446 | 0.09933789 | 0 | 0.045189 | 0.1225003 | 0.06713084 | 0.05162636 | 0.08938484 | 0.3588538 | 0 | 5.549245 | 0.02824313 | 0.03916864 |
| TCGA-5T-A9QA | 0.83013699 | 0 | 0.6009237 | 0.4282445 | 0.09013856 | 0.05341544 | 0.2316814 | 0.1983792 | 0.325465 | 0 | 0.5873282 | 0.1484908 | 5.376212 | 0.03338465 | 0.3703928 |
| TCGA-AN-A0AJ | 0.83013699 | 0 | 0.4271312 | 0.03273036 | 0.1281394 | 0 | 0.3293542 | 0 | 0.2698939 | 0.1502 | 0.4329294 | 0.08795494 | 18.94304 | 0.0632787 | 0.06581797 |
| TCGA-A8-A07W | 0.83287671 | 0 | 0.4148839 | 0.1100488 | 0.1276566 | 0.08510439 | 0.03076062 | 0.2107124 | 0.1944557 | 0.2244511 | 0.1732895 | 0.0394306 | 11.01046 | 0.1063805 | 0.02458875 |
| TCGA-A8-A09A | 0.83287671 | 0 | 0.7832193 | 0.1800504 | 0.08702437 | 0 | 0.1677578 | 0.1723729 | 0.6480799 | 0.3060198 | 0.3307714 | 0.02688011 | 6.972377 | 0 | 0.1005739 |
| TCGA-A8-A07O | 0.83287671 | 0 | 0.5712589 | 0.3877181 | 0.4488462 | 0.04836054 | 0.07865871 | 0.1077635 | 0.01841657 | 0.1434873 | 0.4431226 | 0.06721928 | 12.00563 | 0.1511267 | 0.06287646 |
| TCGA-A8-A099 | 0.83287671 | 0 | 0.5138938 | 0.1737302 | 0.04534357 | 0.08061079 | 0.2913643 | 0.09979328 | 0.3888419 | 0 | 0.2954513 | 0 | 5.288197 | 0.08396958 | 0.2561948 |
| TCGA-D8-A27R | 0.84109589 | 0 | 0.4871732 | 0.1182157 | 0.04059777 | 0.02405794 | 0.1826084 | 0.05360916 | 0.3847913 | 0.04758713 | 0.2939205 | 0.08359901 | 5.020098 | 0.01503621 | 0.08341115 |
| TCGA-A7-A6VV | 0.85753425 | 0 | 0.5232467 | 0.1202866 | 0.2267402 | 0.03100721 | 0.1681114 | 0.6218503 | 0.3542432 | 0.3066647 | 1.098581 | 0.258593 | 3.81189 | 0.1356566 | 0.08062871 |
| TCGA-A7-A3J0 | 0.85753425 | 0 | 0.5085275 | 0.245496 | 0.02542637 | 0.02260122 | 0.07352205 | 0.2853909 | 0.189353 | 0.1788229 | 0.46941 | 0.1413667 | 5.589486 | 0.09888035 | 0.137131 |
| TCGA-A7-A6VX | 0.86849315 | 0 | 1.683017 | 0.1566025 | 0.240431 | 0.0712388 | 0.2317407 | 0.3174879 | 0.2170322 | 0.2818238 | 1.13144 | 0.07426439 | 9.21552 | 0.0890485 | 0.09262188 |
| TCGA-EW-A1PE | 0.87671233 | 0 | 0.3084049 | 0.1701545 | 0.03084049 | 0.3015515 | 0.2080804 | 0.447972 | 0.6681382 | 0.6507005 | 0.7703146 | 0.2286245 | 2.399492 | 0.1370689 | 0.2138538 |
| TCGA-A7-A3IZ | 0.88219178 | 0 | 1.138328 | 0.9912299 | 0.1379792 | 0.09198614 | 0.16624 | 0.3416266 | 0.3970065 | 0.6671522 | 1.348575 | 0.2344048 | 5.596321 | 0.03832756 | 0.4252329 |
| TCGA-A2-A25F | 0.88219178 | 0 | 0.3447937 | 0.6341034 | 0.1477687 | 0.02189166 | 0.0949518 | 0.2764312 | 0.1000409 | 0.1732088 | 0.6418913 | 0.1217143 | 4.168706 | 0.1231406 | 0.1328258 |
| TCGA-AR-A5QQ | 0.88219178 | 1 | 0.13223041 | 0.48636471 | 3.54818257 | 0 | 0.63725497 | 0.11640641 | 0.17904258 | 0 | 0.62226074 | 0.19060239 | 2.394186753 | 0.04897423 | 0.10187897 |
| TCGA-LD-A9QF | 0.88493151 | 0 | 0.39340434 | 0.25121606 | 0.0218558 | 0.0777095 | 0.71623816 | 0.28860475 | 0.11837272 | 0.3074222 | 0.85445288 | 0.13501651 | 2.765418456 | 0.07285266 | 0.10103482 |
| TCGA-A7-A5ZW | 0.89315068 | 0 | 0.3903788 | 0.1794845 | 0.02168771 | 0.1156678 | 0.2090382 | 0.08591556 | 0.02936559 | 0.2287935 | 0.5181499 | 0.1339781 | 5.238833 | 0 | 0.3007734 |
| TCGA-BH-A0GZ | 0.89863014 | 0 | 0.4129184 | 0.227817 | 0 | 0.07340771 | 0.1989968 | 0.02726284 | 0.02795498 | 0.07260103 | 0.717469 | 0.1275424 | 3.130181 | 0.04587982 | 0.0954418 |
| TCGA-C8-A1HF | 0.90958904 | 0 | 0.2522038 | 0.1417237 | 0.02802264 | 0.02490902 | 0.4591662 | 0.1110113 | 0.09485803 | 0.1970823 | 0.2738865 | 0.121179 | 4.143378 | 0.03113627 | 0.2590858 |
| TCGA-E2-A15M | 0.92054795 | 1 | 0.3054447 | 0.4166218 | 0.3665336 | 0.09050212 | 0.1766427 | 0.1882245 | 0.4135781 | 0.2864243 | 1.238364 | 0.113215 | 7.513258 | 0.04525106 | 0.2510235 |
| TCGA-E2-A1IU | 0.92328767 | 0 | 0.6164234 | 0.1720722 | 0.02201512 | 0 | 0.1697552 | 0.2616378 | 0.327898 | 0.6193265 | 1.004129 | 0.0544003 | 4.463256 | 0 | 0 |
| TCGA-E9-A1R6 | 0.92876712 | 0 | 0.1490682 | 0.05140281 | 0.03726704 | 0.03312626 | 0.1796002 | 0.3198712 | 0.1009206 | 0.1965734 | 0.4856519 | 0.09208844 | 6.427122 | 0 | 0.05742594 |
| TCGA-C8-A1HI | 0.93972603 | 0 | 0.3678275 | 0.1522045 | 0 | 0 | 0.141813 | 0.06476193 | 0.1660152 | 0.08623062 | 0.3195605 | 0.04544587 | 4.779493 | 0.02724648 | 0.1322526 |
| TCGA-C8-A1HG | 0.94520548 | 0 | 0.8867397 | 0.2717976 | 0.03694749 | 0 | 0.7834648 | 0 | 0.05002763 | 0 | 0.4012397 | 0.09129881 | 17.12976 | 0.04105276 | 0.2846676 |
| TCGA-A7-A3IY | 0.94520548 | 0 | 0.7289825 | 0.2473832 | 0.03471345 | 0 | 0.7695513 | 0.02291947 | 0.2115122 | 0 | 0.1130936 | 0.1501122 | 5.326773 | 0.07714101 | 0.2942007 |
| TCGA-W8-A86G | 0.95068493 | 0 | 1.256924 | 0.3889685 | 0.02417162 | 0.08594352 | 0.3261712 | 0.829881 | 0.2945596 | 0.3399963 | 1.83748 | 0.4479682 | 4.460224 | 0 | 0.186234 |
| TCGA-AO-A1KS | 0.95890411 | 0 | 0.6520736 | 0.149902 | 0.2018323 | 0.110404 | 0.3292167 | 0.2050143 | 0.6516794 | 0.70974 | 1.213944 | 0 | 6.925191 | 0.01725062 | 0.04784764 |
| TCGA-JL-A3YX | 0.96438356 | 0 | 0.4819052 | 0.1315546 | 0.03011908 | 0.05354503 | 0.2322435 | 0.4772652 | 0.1835182 | 0.5295662 | 0.3597935 | 0.05581914 | 4.383581 | 0.06693128 | 0.1160286 |
| TCGA-C8-A12N | 0.98082192 | 0 | 0.07470981 | 0.2232707 | 0 | 0.03320436 | 0.2880378 | 0.1726444 | 0.2528964 | 0.2627158 | 0.892461 | 0.1153819 | 5.529197 | 0.06225817 | 0.2014646 |
| TCGA-C8-A12P | 0.98082192 | 0 | 0.03642264 | 0.07535718 | 0.07284528 | 0.09712704 | 0.1755308 | 0.1202398 | 0.09863395 | 0.06403981 | 0.435094 | 0.1575033 | 1.195783 | 0.0202348 | 0.02806239 |
| TCGA-C8-A12M | 0.98082192 | 0 | 0.3050671 | 0.2454563 | 0 | 0.4519513 | 0.3430474 | 0.1678499 | 3.407802 | 1.340954 | 1.104315 | 0.03140974 | 11.46147 | 0 | 0.2742176 |
| TCGA-JL-A3YW | 0.98630137 | 0 | 0.1835283 | 0.210952 | 0.04588207 | 0.1903256 | 0.913956 | 0.3029352 | 0.9940036 | 0.1075624 | 0.6975735 | 0.1889609 | 3.015129 | 0.475814 | 0.1414022 |
| TCGA-B6-A0I9 | 0.99178082 | 1 | 0.2121833 | 0.06968252 | 0 | 0.1347195 | 0.204514 | 0.2001336 | 0.3078218 | 0.3197739 | 0.6912758 | 0.03745099 | 4.079924 | 0.03367989 | 0.1868342 |
| TCGA-A7-A426 | 0.99726027 | 0 | 1.400367 | 0.7042073 | 0.0291743 | 0.1296636 | 0.1968387 | 0.654917 | 0.9875646 | 0.3077729 | 2.78806 | 0.1982501 | 3.598213 | 0.03241589 | 0.3821225 |
| TCGA-A8-A07J | 1 | 0 | 0.09343206 | 0.1718291 | 0 | 0.04152536 | 0 | 0.5860388 | 0.1265088 | 0 | 0.6087881 | 0.1154373 | 2.668155 | 0 | 0.5758894 |
| TCGA-A8-A08I | 1 | 0 | 1.12054 | 0.315919 | 0.5391279 | 0.07517252 | 0.3668057 | 0.3350192 | 0.5868546 | 0.4089055 | 0.5280784 | 0.1044869 | 4.666288 | 0.04698282 | 0.5375499 |
| TCGA-A8-A0A1 | 1 | 0 | 0.6756201 | 0.08363113 | 0.2078831 | 0.09239249 | 0.2504616 | 0.3088222 | 0.4574015 | 0.7310175 | 1.185216 | 0.128422 | 4.338249 | 0.02887265 | 0.1201251 |
| TCGA-D8-A1JF | 1.00273973 | 0 | 0.7456225 | 0.1530424 | 0.1464616 | 0.04734111 | 0.0770006 | 0.01758198 | 0.4867654 | 0.04682088 | 0.3759441 | 0.03290116 | 9.485003 | 0.07397048 | 0.02051701 |
| TCGA-C8-A1HK | 1.00273973 | 0 | 0.4990231 | 0.09177437 | 0.07485347 | 0.08871522 | 0.1442958 | 0.1647394 | 0.06756872 | 0.263221 | 0.7045033 | 0.1233107 | 4.293144 | 0 | 0.03844801 |
| TCGA-A7-A5ZV | 1.00821918 | 0 | 0.3450581 | 0.6345896 | 0 | 0 | 0.4988792 | 0.303765 | 0.3893462 | 0 | 0.3747237 | 0.1421089 | 5.411595 | 0 | 0.04430923 |
| TCGA-C8-A130 | 1.01369863 | 0 | 0.2887757 | 0.09127968 | 0.3609696 | 0.3208619 | 0.3827148 | 0.714988 | 0.513198 | 0.06346719 | 1.450412 | 0.1114964 | 4.420301 | 0.02005387 | 0.1112459 |
| TCGA-D8-A1JS | 1.01643836 | 0 | 0.29062 | 0.05196271 | 0 | 0 | 0.1244959 | 0.2345214 | 0.0218614 | 0.05677559 | 0.2454709 | 0.1396373 | 2.783194 | 0 | 0.2239127 |
| TCGA-AC-A6IX | 1.02191781 | 0 | 1.0571979 | 0.3964033 | 0.03375415 | 0.21361555 | 0.4020869 | 0.53763205 | 0.19375143 | 0.11159532 | 0.84027505 | 0.2586018 | 4.7407515 | 0.01875231 | 0.2257217 |
| TCGA-D8-A27W | 1.02191781 | 0 | 0.2137389 | 0.08735178 | 0.05937191 | 0.06333004 | 0.2975749 | 0.1881608 | 0.1929378 | 0.2505364 | 0.1289526 | 0.0293421 | 6.772489 | 0.09235631 | 0.1280832 |
| TCGA-AN-A0XO | 1.02739726 | 0 | 0.3210889 | 0.0861158 | 0.04013611 | 0.09513746 | 0.1547416 | 0.2119982 | 0.1449202 | 0.04704599 | 0.6683299 | 0.03305935 | 3.201805 | 0.04459568 | 0.2267723 |
| TCGA-E9-A295 | 1.02739726 | 0 | 0.04632857 | 0.1384532 | 0 | 0.04118095 | 0.2232702 | 0.3058832 | 0.595933 | 0.2443705 | 0.6540503 | 0.05723993 | 4.84056 | 0 | 0.2855565 |
| TCGA-D8-A1XC | 1.03287671 | 1 | 0.4801051 | 0.08026836 | 0.06546888 | 0 | 0.1262051 | 0.1152685 | 0.1477436 | 0.3069603 | 0.7109742 | 0.02696273 | 4.971669 | 0.02424773 | 0.2353937 |
| TCGA-D8-A27V | 1.04383562 | 0 | 0.7268043 | 0.2052715 | 0.03114876 | 0.07383409 | 0.08006106 | 0.1919483 | 0.393643 | 0.3651136 | 0.5412273 | 0.1282832 | 4.964725 | 0.01153658 | 0.1119955 |
| TCGA-C8-A12V | 1.05479452 | 0 | 1.054601 | 1.124908 | 0.1054601 | 0.2249816 | 0.8131865 | 0.4456307 | 0.08567706 | 0.296679 | 0.9620254 | 0.1563579 | 3.87175 | 0.1640491 | 0.1300054 |
| TCGA-C8-A12X | 1.05479452 | 0 | 0.5116228 | 0.1008124 | 0 | 0 | 0.352236 | 0.4343114 | 0.09896392 | 0 | 0.2381179 | 0.09030298 | 3.286997 | 0 | 0.1689376 |
| TCGA-C8-A12W | 1.05479452 | 0 | 0.9279856 | 0.07757455 | 0.1687247 | 0.03749437 | 0.447222 | 0.1671001 | 0.456913 | 0.4449881 | 0.5496912 | 0 | 5.236108 | 0 | 0.1624957 |
| TCGA-D8-A73W | 1.05479452 | 1 | 1.141824 | 0.2041575 | 0 | 0.5638635 | 0.2445673 | 0.08376514 | 0.3721976 | 0.2230669 | 0.8725851 | 0.1828746 | 4.398304 | 0.1174716 | 0.2280796 |
| TCGA-C8-A12Q | 1.05479452 | 1 | 0.34891 | 0.1871548 | 0.0872275 | 0.07753555 | 0.1961743 | 0.2495642 | 0.2559001 | 0.3067341 | 0.5999357 | 0.0179619 | 2.991487 | 0.04845972 | 0.2240193 |
| TCGA-BH-A0HU | 1.0739726 | 0 | 0.4975693 | 0.03812791 | 0.4975693 | 0.1179423 | 0.09591696 | 0.4380252 | 0.3593166 | 0.2916157 | 0.4683005 | 0.1639353 | 3.781162 | 0.05528547 | 0.2044586 |
| TCGA-C8-A135 | 1.07671233 | 0 | 0.2423853 | 0.2414566 | 0.1009939 | 0.1795446 | 0.3114991 | 0.1066895 | 0.4649421 | 0.3551433 | 0.7458008 | 0 | 6.786304 | 0 | 0.1556246 |
| TCGA-E2-A15H | 1.07671233 | 0 | 0.8676157 | 0.1087919 | 0.2366225 | 0.03505518 | 0.1900582 | 0.3384971 | 0.6941816 | 0.4853794 | 1.199173 | 0.04872535 | 2.781663 | 0.04381898 | 0.1519245 |
| TCGA-C8-A1HN | 1.07945205 | 0 | 0.1635092 | 0.1002355 | 0.06812883 | 0.02422358 | 0.6041304 | 0.1079566 | 0.2582938 | 0.1916591 | 0.6510773 | 0.08417462 | 10.5447 | 0.01513974 | 0.3779346 |
| TCGA-C8-A3M8 | 1.07945205 | 0 | 1.173121 | 0.2169189 | 0.08925918 | 0.340035 | 0.5653593 | 0.2020565 | 0.621559 | 0.3138784 | 0.8031595 | 0.1102816 | 3.968376 | 0.113345 | 0.1178933 |
| TCGA-D8-A1XU | 1.08219178 | 0 | 0.2988985 | 0.132139 | 0.0114961 | 0.02043751 | 0.02216115 | 0.166986 | 0.1867913 | 0.2829809 | 1.023725 | 0.1704441 | 4.193005 | 0.02554688 | 0.1062882 |
| TCGA-C8-A8HP | 1.08493151 | 0 | 0.9595274 | 0.2465317 | 0.1128856 | 0.2006855 | 1.251262 | 0.3726622 | 0.1528493 | 0.1984801 | 1.348497 | 0.3835495 | 2.795369 | 0.1254284 | 0.1304617 |
| TCGA-A8-A06O | 1.08493151 | 0 | 0.7671003 | 0.2045601 | 0.1841041 | 0.1090987 | 0.2661746 | 0.6482895 | 0.2492806 | 0.3776494 | 1.099626 | 0.1137322 | 14.22341 | 0.01704667 | 0.04728195 |
| TCGA-A8-A086 | 1.08493151 | 0 | 0.2283416 | 0.0984231 | 0.0285427 | 0.05074257 | 0.4676876 | 0.2072977 | 0.2125606 | 0.2509248 | 0.5269421 | 0.1763255 | 9.769782 | 0.01585705 | 0.08796465 |
| TCGA-A8-A0A4 | 1.08493151 | 0 | 0.6333919 | 0.1747288 | 0.4011482 | 0.07506867 | 0 | 0.3624361 | 0.2572875 | 0.2227312 | 0.5502771 | 0.07825691 | 4.728989 | 0.09383584 | 0.1626689 |
| TCGA-D8-A27H | 1.08767123 | 0 | 0.3297815 | 0.8270382 | 0.05996027 | 0.1598941 | 0.1444826 | 0.7125953 | 0.7915771 | 0.316274 | 1.269747 | 0.05556164 | 8.345943 | 0.2165232 | 0.2078879 |
| TCGA-D8-A27T | 1.09041096 | 0 | 0.31276998 | 0.22289355 | 0.031277 | 0 | 0.45219756 | 0.1652045 | 0.38114705 | 0.38494767 | 0.81518329 | 0.09660849 | 4.097396022 | 0.03475222 | 0.07229354 |
| TCGA-LD-A74U | 1.10136986 | 0 | 0.4680828 | 0.3676513 | 0.07801381 | 0.2080368 | 0.2631791 | 0.3863132 | 0.5017531 | 0.6858357 | 2.160382 | 0.2650662 | 3.153269 | 0.043341 | 0.3906952 |
| TCGA-D8-A140 | 1.10410959 | 0 | 0.4550523 | 0.2092195 | 0.3516313 | 0.1103157 | 0.03987315 | 0.5189542 | 0.728177 | 0.6546207 | 1.617298 | 0.07666729 | 5.931604 | 0 | 0.1593646 |
| TCGA-LD-A7W6 | 1.10684932 | 0 | 1.902212 | 0.2946955 | 0.08270485 | 0.1470308 | 0.2391466 | 0.4641483 | 1.063848 | 0.6543681 | 2.200473 | 0.4087344 | 4.006829 | 0.09189428 | 0.2230243 |
| TCGA-A7-A3RF | 1.11780822 | 0 | 1.039003 | 0.1264506 | 0.03055891 | 0.02716347 | 0.2945437 | 0.3228231 | 0.1241321 | 0.2149198 | 0.6637228 | 0 | 2.838942 | 0.06790868 | 0.1648121 |
| TCGA-D8-A27G | 1.12054795 | 0 | 0.54050563 | 0.1449632 | 0.04504214 | 0.13345818 | 0.2315419 | 0.13878182 | 0.16263451 | 0.15838993 | 1.04351011 | 0.09275086 | 3.482838733 | 0.03336455 | 0.34703411 |
| TCGA-D8-A27M | 1.12328767 | 0 | 0.64380015 | 0.41036373 | 0.39505918 | 0.05202426 | 0.28205921 | 0.48303125 | 0.49529438 | 0.51452559 | 1.39829897 | 0.10846756 | 7.379756083 | 0.13006064 | 0.31565278 |
| TCGA-D8-A145 | 1.12328767 | 0 | 0.6774353 | 0.2725315 | 0.1058493 | 0.1881765 | 0.1632374 | 0.4193204 | 0.429966 | 0.07444344 | 0.6896966 | 0.130779 | 3.872508 | 0.02352206 | 0.2935916 |
| TCGA-GM-A5PV | 1.12876712 | 0 | 0.5725303 | 0.1880231 | 0.02044751 | 0 | 0.1970844 | 0.3780117 | 0.9690217 | 0.43142 | 0.6661632 | 0.1263167 | 4.576649 | 0 | 0.2520656 |
| TCGA-AC-A6IW | 1.13150685 | 0 | 1.067044 | 1.097024 | 0.0889203 | 0.07904027 | 0.5428067 | 0.1761282 | 0 | 0.05211446 | 0.4184485 | 0.09155244 | 3.272101 | 0.01646672 | 0.3653868 |
| TCGA-BH-A0HP | 1.13424658 | 0 | 0.3110772 | 0.2931992 | 0.09332316 | 0.05530261 | 0 | 0.2670044 | 0.273783 | 0.05469489 | 0.8107713 | 0.07686849 | 3.704971 | 0.01728207 | 0.2876091 |
| TCGA-AC-A62X | 1.14246575 | 0 | 0.28449074 | 0.05605729 | 0.02032077 | 0.03612581 | 0.47007076 | 0.08050043 | 0.22011776 | 0.07145764 | 0.52962724 | 0.07532022 | 2.902329044 | 0.02257863 | 0.1878774 |
| TCGA-BH-A0DK | 1.15890411 | 0 | 0.5650258 | 0.4082288 | 0.02017949 | 0.03587466 | 0.2723016 | 0.1865285 | 0.1639406 | 0.07096086 | 0.6136027 | 0.1495932 | 3.178749 | 0 | 0.2798569 |
| TCGA-D8-A142 | 1.16438356 | 0 | 0.3975938 | 0.1256762 | 0.06212402 | 0.1987969 | 0.1916114 | 0.1312552 | 0.2691749 | 0.349533 | 0.512734 | 0.04605333 | 4.45432 | 0.09663737 | 0.1340204 |
| TCGA-D8-A1JH | 1.16712329 | 0 | 0.8034713 | 0.2539708 | 0.02510848 | 0.1785492 | 0.09680377 | 0.2320894 | 0.5949541 | 0.794642 | 1.036151 | 0.07755515 | 5.088384 | 0.02789831 | 0.1741069 |
| TCGA-A8-A07I | 1.16712329 | 0 | 0.2592663 | 0.1192029 | 0.1296331 | 0.03840982 | 0.2498952 | 0.1426499 | 0.380306 | 0 | 0.6100383 | 0.1334704 | 4.853099 | 0.04801227 | 0.09987786 |
| TCGA-OL-A66P | 1.17260274 | 0 | 0.6343122 | 0.3976879 | 0.02883237 | 0 | 1.056029 | 0.152292 | 0.3123168 | 0 | 0.1252447 | 0.178115 | 2.708487 | 0.1922158 | 0.08885739 |
| TCGA-E2-A15S | 1.17260274 | 0 | 0 | 0.1544036 | 0.2518709 | 0.04975227 | 0.1888188 | 0.2217295 | 1.87571 | 0.5412609 | 1.124491 | 0.06915373 | 10.44872 | 0 | 0.3234297 |
| TCGA-D8-A1Y3 | 1.17808219 | 0 | 0.4164587 | 0.04786882 | 0.02974705 | 0 | 0.7167964 | 0.0982021 | 0.3423638 | 0 | 0.03230449 | 0.03675311 | 9.778062 | 0.0826307 | 0.1375145 |
| TCGA-D8-A143 | 1.18082192 | 0 | 1.21719317 | 0.20760432 | 0.01963215 | 0.17450798 | 0.07569021 | 0.15554494 | 0.23924084 | 0.34518062 | 0.08527992 | 0 | 3.556721819 | 0 | 0.33276963 |
| TCGA-D8-A1J8 | 1.18082192 | 0 | 0.166829 | 0.2428928 | 0.166829 | 0.09886161 | 0.4555972 | 0.1468646 | 0.4706036 | 0.09777522 | 1.721131 | 0.1030604 | 4.54872 | 0 | 0.1713813 |
| TCGA-A1-A0SG | 1.1890411 | 0 | 0.463428 | 0.2458504 | 0 | 0.06337477 | 0.06871963 | 0.4236608 | 0.1689398 | 0.3760701 | 0.6968357 | 0.06606636 | 4.880775 | 0.07921846 | 0.1098632 |
| TCGA-BH-A0E2 | 1.19178082 | 0 | 0.3039074 | 0.1222616 | 0.01899421 | 0 | 0.2563074 | 0.1504906 | 0.05143708 | 0.2003785 | 0.412544 | 0.0469355 | 2.819071 | 0 | 0.02926877 |
| TCGA-D8-A27I | 1.20273973 | 0 | 0.7342708 | 0.3895336 | 0.04236177 | 0.1004131 | 0.1633225 | 0.1864617 | 0.650065 | 0.297929 | 1.134759 | 0.1046777 | 5.380347 | 0.03137909 | 0.1958297 |
| TCGA-EW-A1PF | 1.20273973 | 0 | 0.401202 | 0.2536334 | 0.0334335 | 0.2971867 | 0.03222506 | 0.1545207 | 0.3395221 | 0.5878417 | 0.4356945 | 0.0619617 | 3.188785 | 0.0557225 | 0.1287968 |
| TCGA-C8-A27B | 1.20273973 | 0 | 0.43809088 | 0.19549695 | 0.2963556 | 0.04581343 | 0.34774046 | 0.05104384 | 0.0872329 | 0.04530998 | 0.75561057 | 0.07959862 | 5.774138445 | 0.08590017 | 0.11912961 |
| TCGA-LL-A5YO | 1.20547945 | 0 | 0.6616727 | 0.5197046 | 0 | 0 | 0.6377568 | 0.5824904 | 0.3359692 | 0.1938968 | 0.1796396 | 0.1021888 | 7.950202 | 0.3675959 | 0.042483 |
| TCGA-D8-A1XK | 1.20821918 | 0 | 0.3927056 | 0.4836276 | 0.2244032 | 0.02493369 | 1.000352 | 0.01852021 | 0.3228367 | 0.1479581 | 0.5483155 | 0.1386275 | 6.836287 | 0.06233422 | 0.04322373 |
| TCGA-A7-A4SD | 1.20821918 | 0 | 1.854451 | 0.4608763 | 0.1503609 | 0.356411 | 0.869557 | 0.7942034 | 0.1017958 | 0.881236 | 1.143015 | 0.2476988 | 3.483148 | 0.1392231 | 0.07723192 |
| TCGA-LL-A740 | 1.20821918 | 0 | 0.4980191 | 0.149714 | 0.1915458 | 0.1362103 | 0 | 0.07588059 | 0.1815497 | 0.06735676 | 0.5408352 | 0.07099767 | 2.36243 | 0.0638486 | 0.05903177 |
| TCGA-AC-A2QJ | 1.22191781 | 1 | 0.5220776 | 0.2828992 | 0.03729125 | 0.06629556 | 0.4313205 | 0.09848584 | 0.07573964 | 0 | 0.04049729 | 0.04607414 | 4.518888 | 0.1450215 | 0.1723897 |
| TCGA-A7-A4SC | 1.22191781 | 0 | 1.70864 | 0.2487676 | 0.05695468 | 0.2025055 | 0.1646882 | 0.2256251 | 1.311002 | 0.6008406 | 2.659603 | 0.2814749 | 2.788874 | 0 | 0.3071713 |
| TCGA-LL-A5YN | 1.22465753 | 0 | 1.091886 | 0.3164887 | 0 | 0 | 1.693024 | 0.8462918 | 0.642798 | 0 | 0.1546642 | 0.1466357 | 3.094416 | 0 | 0.2926124 |
| TCGA-A7-A425 | 1.22465753 | 0 | 0.74427912 | 0.3421973 | 0.01860698 | 0.19847443 | 0.50216422 | 0.63883104 | 1.00776862 | 0.06543113 | 1.77818721 | 0.06896795 | 1.970871909 | 0.06202326 | 0.25804862 |
| TCGA-D8-A1JU | 1.22465753 | 0 | 0.3041608 | 0.5104308 | 0.01520804 | 0.2162921 | 0.1172668 | 0.28115 | 0.1647359 | 0.4278306 | 0.6275897 | 0.05636957 | 5.663496 | 0.05069347 | 0.2812145 |
| TCGA-D8-A1XG | 1.22739726 | 0 | 0.8649066 | 0.1469606 | 0.05640695 | 0 | 0.1449817 | 0.07448511 | 0.07637613 | 0.1983541 | 0.4900514 | 0.02323066 | 4.833219 | 0.06267439 | 0.3476769 |
| TCGA-A7-A56D | 1.22739726 | 0 | 1.227855 | 0.1411328 | 0 | 0 | 0.641049 | 0.439123 | 0.4502714 | 0 | 0.05555907 | 0.09481509 | 0.9235991 | 0.1421129 | 0 |
| TCGA-LL-A5YP | 1.23287671 | 0 | 0.4871913 | 0.2341776 | 0.1771605 | 0.118107 | 1.237989 | 0.1754547 | 0.05996969 | 0.3114909 | 1.058153 | 0.1368034 | 4.532053 | 0 | 0.307116 |
| TCGA-E2-A158 | 1.23287671 | 0 | 0.7122219 | 0.1488447 | 0.09712117 | 0.2302131 | 0.1248144 | 0.1496227 | 0.2191733 | 0.341525 | 1.300808 | 0.03999842 | 7.975282 | 0.0359708 | 1.097485 |
| TCGA-AC-A3OD | 1.23561644 | 0 | 6.97951 | 1.395204 | 0.303457 | 0.1348698 | 2.339909 | 0.6010702 | 6.471466 | 3.201304 | 2.636368 | 1.687174 | 3.429988 | 1.011523 | 0.9352125 |
| TCGA-A7-A4SA | 1.24383562 | 0 | 0.9328708 | 0.6307443 | 0.08231213 | 0.1951102 | 0.1057827 | 0.8695422 | 1.040221 | 0.5788985 | 1.19185 | 0.5423914 | 4.462306 | 0.09145792 | 0.1268373 |
| TCGA-AC-A5XU | 1.24657534 | 0 | 1.367381 | 0.1109437 | 0.02010854 | 0.07149704 | 0.930323 | 0.2124259 | 0.2178189 | 0.2121341 | 0.524096 | 0.04968907 | 2.807767 | 0 | 0.3408447 |
| TCGA-D8-A1XV | 1.2630137 | 0 | 0.7896752 | 0.07059676 | 0.1096771 | 0 | 0.1268554 | 0.2606906 | 0.207907 | 0.6942199 | 0.8575658 | 0.08130504 | 5.014812 | 0 | 0.5070146 |
| TCGA-BH-A0H0 | 1.2630137 | 0 | 1.511124 | 0.2463275 | 0.5632373 | 0.04884443 | 0.1059277 | 0.8344536 | 0.8184369 | 0.1932307 | 1.014461 | 0.0509189 | 6.656547 | 0.01526388 | 0.08467413 |
| TCGA-EW-A6S9 | 1.26849315 | 0 | 0.6111317 | 0.05784884 | 0.08987231 | 0.1278184 | 0.06929913 | 0.1424111 | 0.3894044 | 0.4424483 | 0.4684746 | 0.1110391 | 2.702182 | 0 | 0.4431585 |
| TCGA-D8-A1XZ | 1.27671233 | 0 | 0.34128666 | 0.16345147 | 0 | 0 | 0.328951 | 0.2441115 | 0.34658166 | 0.05000537 | 1.01922713 | 0.08784727 | 10.16646897 | 0 | 0.24103713 |
| TCGA-XX-A899 | 1.27945205 | 0 | 1.27898558 | 0.53291066 | 0.0799366 | 0.28421902 | 0.42376028 | 0.55416844 | 0.35176613 | 0.35136966 | 1.95320196 | 0.19752673 | 5.207252816 | 0.02220461 | 0.27714776 |
| TCGA-C8-A26Z | 1.28767123 | 0 | 0.4933218 | 0.06003917 | 0.01450947 | 0.0257946 | 0.4754909 | 0.3640339 | 0.3536298 | 0.1020446 | 0.7248167 | 0.05378026 | 4.031067 | 0.03224326 | 0.1117904 |
| TCGA-AQ-A1H2 | 1.30136986 | 0 | 0.5831043 | 0.0548374 | 0 | 0 | 0.1788272 | 0.1924966 | 0.5562631 | 0.04660174 | 0.7195857 | 0.06549434 | 9.567643 | 0.01472486 | 0.2246309 |
| TCGA-BH-A0DP | 1.30410959 | 0 | 0.292983 | 0.2405443 | 0.08370942 | 0.03720419 | 0.1210257 | 0.2487102 | 0.5100489 | 0.2207721 | 0.3636246 | 0 | 3.667668 | 0 | 0.2579809 |
| TCGA-EW-A1J5 | 1.30684932 | 0 | 2.047124 | 0.2323231 | 0.3886945 | 0.1382025 | 0.2997162 | 0.633032 | 0.5087567 | 0.6378576 | 1.632165 | 0.2881442 | 3.688619 | 0.04318828 | 0.3593708 |
| TCGA-BH-A0E1 | 1.30684932 | 0 | 0.1549869 | 0.1425167 | 0.0581201 | 0.03444154 | 0.2240775 | 0.1023297 | 0 | 0 | 0.2524674 | 0 | 3.107476 | 0 | 0.08955906 |
| TCGA-D8-A1JC | 1.31506849 | 0 | 0.3777018 | 0.1215592 | 0.08813041 | 0 | 0.12135 | 0.1828762 | 0.2557078 | 0.3541819 | 0.738313 | 0.03111057 | 6.617481 | 0.04196686 | 0.03880082 |
| TCGA-D8-A27F | 1.3369863 | 0 | 0.1341844 | 1.042628 | 0.3354609 | 0.214695 | 1.060542 | 0.3720986 | 0.2361948 | 0.1415571 | 1.020044 | 0.09947259 | 5.256437 | 0.08945625 | 0.186092 |
| TCGA-XX-A89A | 1.3369863 | 0 | 0.21955207 | 0.33311349 | 0 | 0.07806296 | 0.29626304 | 0.8987441 | 0.50537233 | 0.46323074 | 1.81204967 | 0.29838737 | 3.3181629 | 0.02439468 | 0.03383146 |
| TCGA-LL-A6FR | 1.33972603 | 0 | 0.496869 | 0.2170232 | 0 | 0 | 1.005711 | 0.5248905 | 0.1009156 | 0 | 0 | 0.06138922 | 1.797956 | 0.165623 | 0.3062563 |
| TCGA-D8-A73U | 1.34794521 | 0 | 1.565326 | 0.4523767 | 0 | 0.1590173 | 0.3879638 | 0.7972736 | 0.1513916 | 0.4718095 | 1.991314 | 0.3315418 | 2.761565 | 0.02484645 | 0.17229 |
| TCGA-BH-A0DZ | 1.35616438 | 0 | 0.4595772 | 0.05282496 | 0 | 0 | 0.8859319 | 0 | 0 | 0 | 0.3327255 | 0.2839087 | 3.456962 | 0 | 0.05901472 |
| TCGA-AC-A3HN | 1.35890411 | 0 | 0.2947354 | 0.3510954 | 0.06698531 | 0.142902 | 0.232431 | 0.4245781 | 0.4172174 | 0.4239949 | 0.9602238 | 0.2648377 | 3.073499 | 0.02977125 | 0.1857955 |
| TCGA-D8-A1XQ | 1.36712329 | 0 | 0.23825335 | 0.13008085 | 0.01489084 | 0.15883557 | 1.11950371 | 0.0786531 | 0.40324968 | 0.05236337 | 0.51747335 | 0.16558148 | 8.911603207 | 0.03309074 | 0.57364371 |
| TCGA-D8-A27L | 1.36712329 | 0 | 0.4272623 | 0.2209977 | 0.04005584 | 0.09494718 | 0.1286935 | 0.387886 | 0.3434972 | 0.7981823 | 1.478985 | 0.1319729 | 5.669811 | 0.1038485 | 0.1028721 |
| TCGA-E2-A1BC | 1.37260274 | 0 | 0.3986541 | 0.238276 | 0 | 0 | 0.07684899 | 0.3684946 | 0.7556998 | 0.1401861 | 1.298783 | 0 | 4.993227 | 0 | 0.4914388 |
| TCGA-D8-A1XY | 1.37808219 | 0 | 0.8395773 | 0.1187731 | 0.03229143 | 0.2009245 | 0.2801185 | 0.2558441 | 0.174893 | 0.1703284 | 1.016961 | 0.0797935 | 4.127113 | 0.1076381 | 0.1243972 |
| TCGA-EW-A1J3 | 1.38082192 | 0 | 1.121154 | 0.1424333 | 0.1770243 | 0.2622582 | 0.540315 | 0.2727196 | 1.05865 | 0.7781286 | 2.531207 | 0.291623 | 4.147947 | 0.01639113 | 0.3637095 |
| TCGA-C8-A274 | 1.39178082 | 0 | 0.4125626 | 0.05690518 | 0.0962646 | 0 | 0.2120804 | 0.2179148 | 0.4282737 | 0.8704617 | 0.9856699 | 0.06796398 | 2.717736 | 0.04584029 | 0.1483371 |
| TCGA-D8-A1X7 | 1.39452055 | 0 | 0.6255687 | 0.05229415 | 0.1421747 | 0.1769285 | 0.02740717 | 0.1126446 | 0.07700294 | 0.299973 | 0.6484711 | 0.07026395 | 4.279295 | 0 | 0.1314488 |
| TCGA-C8-A273 | 1.40547945 | 0 | 0.3976598 | 0.1305944 | 0.07101068 | 0 | 0.08213284 | 0.09376926 | 0.1538398 | 0.5493574 | 0.6169254 | 0 | 2.377847 | 0.01578015 | 0.1531916 |
| TCGA-BH-A0HN | 1.41369863 | 0 | 0.8780559 | 0.08971197 | 0.07317132 | 0.04336078 | 0.2350886 | 0.2576597 | 0.1651257 | 0.08576858 | 0.741646 | 0.06026981 | 1.878174 | 0 | 0.2630879 |
| TCGA-UL-AAZ6 | 1.41917808 | 0 | 1.077561 | 0.09588974 | 0 | 0.06179561 | 0.3015328 | 0.3442534 | 0.611855 | 0 | 0.5662267 | 0.06442013 | 2.471207 | 0.01931113 | 0.1071256 |
| TCGA-A2-A0T1 | 1.42739726 | 0 | 0.2621708 | 0.2561439 | 0.08192838 | 0.1165204 | 0.03158684 | 0.1947348 | 0.1109326 | 0.1728599 | 0.533832 | 0.2429382 | 5.018287 | 0 | 0.05049839 |
| TCGA-D8-A1XD | 1.43013699 | 0 | 0.6134028 | 0.2316628 | 0.04381449 | 0.03894621 | 0 | 0.4339261 | 0.9788737 | 0.847401 | 1.094371 | 0.08120059 | 1.753454 | 0 | 0.0675151 |
| TCGA-A2-A25C | 1.43287671 | 0 | 0.8431814 | 0.1530275 | 0.1220394 | 0.1972354 | 0.1497088 | 0.2051031 | 0.1502216 | 0.5461904 | 0.7710923 | 0.1096598 | 3.887005 | 0.09861771 | 0.1196709 |
| TCGA-A2-A1FW | 1.44657534 | 0 | 1.780037 | 0.1716017 | 0.04306542 | 0.07656075 | 0.2213805 | 0.2274707 | 2.060337 | 0.6057554 | 1.184786 | 0.1064165 | 10.13675 | 0.03190031 | 0.1106014 |
| TCGA-OL-A66O | 1.44657534 | 0 | 1.055188 | 0.1408482 | 0 | 0 | 0.196848 | 0.6292639 | 3.710128 | 0 | 0.1478587 | 0.1892477 | 2.70811 | 0.01891017 | 0.2884788 |
| TCGA-AC-A62Y | 1.45205479 | 0 | 0.6086492 | 0.1772312 | 0.02028831 | 0.0721362 | 0.5475399 | 0.3214873 | 1.318597 | 0.2140305 | 0.7050416 | 0.1754664 | 3.176442 | 0.09017025 | 0.1875773 |
| TCGA-D8-A27E | 1.45205479 | 0 | 0.3540899 | 0.2003692 | 0.01361884 | 0.02421128 | 0.2100255 | 0.08991808 | 0.4978849 | 0.4310139 | 0.384532 | 0.08413185 | 4.251418 | 0.0302641 | 0.1259142 |
| TCGA-A2-A0T5 | 1.45479452 | 0 | 0.4495792 | 0.1431021 | 0.08645754 | 0.03074046 | 0.1999982 | 0.1826668 | 0.09365217 | 0.1216106 | 0.7886805 | 0.1068201 | 3.127147 | 0.03842557 | 0.2930952 |
| TCGA-LL-A9Q3 | 1.45753425 | 0 | 0 | 0.08622921 | 0 | 0 | 0.09038483 | 0.1547856 | 0.1587153 | 0 | 0.2545908 | 0.08689507 | 2.325864 | 0.02604841 | 0 |
| TCGA-D8-A1J9 | 1.45753425 | 0 | 0.09374579 | 0.0969784 | 0.1171822 | 0.06249719 | 0.09035739 | 0.06189543 | 0.1110669 | 0.08241388 | 0.7635404 | 0.01447811 | 4.433151 | 0 | 0.2347407 |
| TCGA-EW-A3U0 | 1.45753425 | 0 | 1.37036899 | 0.69306018 | 0.0587301 | 0.45243929 | 1.09440828 | 0.51701876 | 0.07952172 | 0.27536457 | 1.10550776 | 0.19349943 | 3.299597543 | 0.04350378 | 0.24133075 |
| TCGA-A2-A0T0 | 1.46027397 | 0 | 0.4355916 | 0.05722058 | 0.04667053 | 0.08296983 | 0.1499455 | 0.06162821 | 0.3159641 | 0.1094108 | 0.6419838 | 0.05766243 | 4.858892 | 0.1728538 | 0.07191607 |
| TCGA-EW-A423 | 1.46027397 | 0 | 0.9666595 | 0.229389 | 0.04677385 | 0.2494605 | 0.5409987 | 0.4117643 | 0.3377745 | 0.1644795 | 0.6095415 | 0.2889504 | 1.970277 | 0.05197094 | 0.2883011 |
| TCGA-BH-A1EY | 1.4739726 | 1 | 0.4169912 | 0.1597668 | 0.02316618 | 0.08236864 | 0.0446577 | 0.183545 | 0.2509397 | 0.08146349 | 1.006314 | 0.02862231 | 5.74401 | 0 | 0.1784874 |
| TCGA-D8-A1XM | 1.4739726 | 0 | 0.2651953 | 0.1889898 | 0.0795586 | 0.09429167 | 0.178927 | 0.3676986 | 0.08976993 | 0.559533 | 0.8063858 | 0.08191361 | 4.090601 | 0.02946615 | 0.2043238 |
| TCGA-AO-A1KT | 1.48219178 | 0 | 0.6934757 | 0.1062798 | 0.07223705 | 0.05136857 | 0.1671026 | 0.03815547 | 0.3129932 | 0 | 0.6589588 | 0.07140033 | 6.088829 | 0.06421071 | 0.3784618 |
| TCGA-A7-A4SF | 1.49315068 | 0 | 0.11905653 | 0.09579261 | 0.32740545 | 0 | 0.05737664 | 0.47164072 | 0.48361467 | 0.20933016 | 0.77575294 | 0.03677422 | 2.738953971 | 0 | 0.36691578 |
| TCGA-A8-A06R | 1.49863014 | 0 | 0.6355602 | 0.2435097 | 0 | 0 | 0.2041961 | 0.6527533 | 0.07171343 | 0 | 1.610469 | 0.2617494 | 4.648153 | 0.05884817 | 0.4624729 |
| TCGA-A8-A082 | 1.50410959 | 0 | 0.1949528 | 0.358534 | 0.2534387 | 0 | 0.03758127 | 0.4376383 | 0 | 0.06855484 | 0.2963989 | 0.2408684 | 5.821138 | 0.06498428 | 0.03004089 |
| TCGA-GM-A5PX | 1.50958904 | 0 | 0.7515977 | 0.4415516 | 0 | 0 | 0.5232005 | 0.1378446 | 0.3674949 | 0 | 0.4081073 | 0 | 4.873065 | 0.09278984 | 0.5147378 |
| TCGA-D8-A1JD | 1.51232877 | 0 | 0.3834254 | 0.1211977 | 0.1677486 | 0.02130141 | 0.1847833 | 0.205689 | 0.3407024 | 0.252808 | 0.520487 | 0.1332366 | 6.117961 | 0.01331338 | 0.2400255 |
| TCGA-D8-A1XB | 1.51232877 | 0 | 0.4064592 | 0.1764957 | 0.04516214 | 0.08028824 | 0.2176489 | 0.149091 | 0.1834513 | 0.7146536 | 0.7847177 | 0.02789939 | 4.433691 | 0.07527023 | 0.2087752 |
| TCGA-A1-A0SQ | 1.51780822 | 0 | 0.5223351 | 0.07719238 | 0 | 0 | 0.1438444 | 0.07390082 | 1.01036 | 0.06559938 | 0.3241381 | 0.04609686 | 3.26641 | 0.02072758 | 0.4024412 |
| TCGA-BH-A0BD | 1.51780822 | 0 | 0.6740599 | 0.119739 | 0.2297931 | 0.1906432 | 0.1476582 | 0.08091753 | 0.08297185 | 0.05387092 | 0.6987375 | 0.05678286 | 3.612445 | 0.08510857 | 0.02360636 |
| TCGA-EW-A1IZ | 1.51780822 | 0 | 0.36791878 | 0.42289515 | 0 | 0.04087986 | 0.57625833 | 0.51619966 | 0.43589813 | 0.24258381 | 0.49943726 | 0.22728573 | 4.202686817 | 0.05109983 | 0.14173436 |
| TCGA-B6-A0WW | 1.52876712 | 1 | 0.60110374 | 0.05024901 | 0 | 0 | 0.36869452 | 0.36079755 | 0.48094465 | 0 | 0.1186877 | 0.13503208 | 7.979431895 | 0 | 0.2105135 |
| TCGA-S3-A6ZG | 1.53972603 | 0 | 0.9859016 | 0.08596843 | 0.3569644 | 0.151096 | 0.1966069 | 0.2020156 | 0.4142887 | 1.016162 | 1.624453 | 0.1260106 | 5.238499 | 0 | 0.1833525 |
| TCGA-EW-A1P6 | 1.53972603 | 0 | 0.5791119 | 0.03630796 | 0.07896981 | 0.04679692 | 0.2029746 | 0.2433183 | 0.1069267 | 0.277696 | 0.2286908 | 0.1300918 | 9.88693 | 0.02924808 | 0.121687 |
| TCGA-D8-A3Z6 | 1.54246575 | 0 | 0.7841095 | 0.2178082 | 0 | 0.1452055 | 0.1889421 | 0.9706996 | 0.2875437 | 1.033991 | 1.596603 | 0.222013 | 2.89164 | 0.1270548 | 0.2013764 |
| TCGA-D8-A1X5 | 1.54794521 | 0 | 0.2493175 | 0.06686676 | 0.1246587 | 0.03693592 | 0.160204 | 0.1646113 | 0.5063712 | 0.7306007 | 0.3610027 | 0.07700926 | 4.580571 | 0.0461699 | 0.09604526 |
| TCGA-A2-A0YJ | 1.55068493 | 0 | 0.1191747 | 0.0456608 | 0.09931223 | 0 | 0.1531562 | 0.05245653 | 0.2958356 | 0.06984597 | 0.2157008 | 0 | 4.553587 | 0.8165672 | 0.03060666 |
| TCGA-BH-A5IZ | 1.55342466 | 0 | 0.2513519 | 0.0481517 | 0.06283797 | 0.1489493 | 0.3230225 | 0.02765908 | 0.1701677 | 0.07365623 | 0.1819742 | 0.3105506 | 3.316222 | 0.04654664 | 0.1291053 |
| TCGA-AC-A6IV | 1.55616438 | 0 | 1.14482106 | 0.51837944 | 0.10407464 | 0.30836931 | 0.26750109 | 0.80167537 | 0.16440564 | 0.12199226 | 1.69533354 | 0.47148358 | 3.375281454 | 0.03854616 | 0.16037184 |
| TCGA-B6-A0IK | 1.56438356 | 1 | 0.3415102 | 0.07850808 | 0.02845918 | 0 | 0 | 0.03758021 | 0.03853429 | 0 | 0.6799297 | 0.2109715 | 5.415059 | 0.09486393 | 0 |
| TCGA-S3-A6ZF | 1.56712329 | 0 | 0.4127979 | 0.1660681 | 0.1031995 | 0.3057762 | 0.3315646 | 0.227124 | 0.2794682 | 0.2419328 | 1.008646 | 0.1062543 | 4.616412 | 0.03822202 | 0.1855271 |
| TCGA-B6-A409 | 1.56986301 | 1 | 0.7152576 | 0.3699608 | 0.0149012 | 0.07947307 | 0.08617562 | 0.1377387 | 0.1614122 | 0.1571995 | 0.1941876 | 0.1104645 | 8.151247 | 5.231977 | 0.160732 |
| TCGA-S3-AA12 | 1.57260274 | 0 | 0.9740303 | 0.5100286 | 0 | 0.09620052 | 0.1043138 | 0.1429114 | 0.4029838 | 0.1902867 | 1.116535 | 0.1002863 | 7.940505 | 0 | 0.1250761 |
| TCGA-BH-A8FZ | 1.57260274 | 0 | 0.657724 | 0.5008531 | 0.02055388 | 0.328862 | 1.386768 | 0.4342607 | 1.363687 | 0.2891095 | 1.830318 | 0.304737 | 4.675981 | 0.04567528 | 0.2850489 |
| TCGA-A2-A0T6 | 1.57534247 | 0 | 0.4424513 | 0.2847962 | 0.01474838 | 0.1048773 | 0.142153 | 0.1947516 | 0.2196655 | 0.2074497 | 0.9609802 | 0.1457755 | 3.522528 | 0.01638708 | 0.3408934 |
| TCGA-EW-A1PA | 1.57534247 | 0 | 0.6058359 | 0.2249788 | 0.09320552 | 0.4556714 | 0.5390199 | 0.1230774 | 1.924582 | 0.983267 | 2.27742 | 0.1151574 | 4.325704 | 0 | 0.07181164 |
| TCGA-E9-A1NH | 1.57808219 | 0 | 0.4729134 | 0.1482487 | 0.02149606 | 0.03821522 | 0.3315056 | 0.1419272 | 0.2619549 | 0.2267717 | 1.307272 | 0.2124707 | 2.606572 | 0.09553806 | 0.5631072 |
| TCGA-D8-A1JI | 1.58082192 | 0 | 1.418395 | 0.1534436 | 0.02085874 | 0.1483288 | 0.1206289 | 0.1377193 | 0.3389178 | 0.1466989 | 0.5436487 | 0.2061714 | 6.408206 | 0 | 0.3535607 |
| TCGA-A8-A0A2 | 1.58630137 | 0 | 0.8667043 | 0.1735337 | 0.1258119 | 0.07455521 | 0.1347383 | 0.4245647 | 0.984255 | 0.3932583 | 1.335921 | 0.1727148 | 6.597187 | 0.1553234 | 0.4738983 |
| TCGA-A8-A09T | 1.58630137 | 0 | 0.4161102 | 0.3061041 | 0 | 0 | 0.160428 | 0 | 0.05634214 | 0 | 0 | 0.0514113 | 8.914688 | 0 | 0.1282394 |
| TCGA-D8-A147 | 1.6 | 0 | 0.9654079 | 1.371228 | 0.3275491 | 0.1225915 | 0.1329305 | 0.7967603 | 0.07002757 | 0.1212443 | 1.422838 | 0.06389903 | 5.97381 | 0.07661967 | 0.2125181 |
| TCGA-A2-A1G1 | 1.6 | 0 | 0.3735416 | 0.2060919 | 0.323736 | 0.06640739 | 0.04800534 | 0.1644199 | 0.3034696 | 0.2627105 | 0.8383557 | 0.2153753 | 5.627554 | 0.06917436 | 0 |
| TCGA-A1-A0SP | 1.6 | 0 | 0.9046077 | 1.143757 | 0 | 0.06185352 | 0.3688855 | 0.0689152 | 0.3062142 | 0.1835214 | 0.3778382 | 0.08597401 | 7.437245 | 0.03865845 | 0.1876455 |
| TCGA-AC-A23C | 1.60273973 | 0 | 0.9087617 | 0.16974 | 0.141994 | 0.1514603 | 0.05474468 | 0.3000039 | 0.2499415 | 0.5991835 | 1.418655 | 0.2982423 | 3.988026 | 0.2524338 | 0.218803 |
| TCGA-A2-A0YK | 1.6109589 | 0 | 0.9901599 | 0.5817031 | 0.01833629 | 0.1629893 | 0.1413883 | 0.3631948 | 0.670348 | 0.3223964 | 1.354065 | 0.3171683 | 4.798378 | 0.02037366 | 0.2542947 |
| TCGA-D8-A1JM | 1.61643836 | 0 | 0.44713929 | 0.19273245 | 0.06986551 | 0 | 0.08080831 | 0.20296556 | 0.43515671 | 0.04913619 | 0.78906933 | 0.06905626 | 4.475857569 | 0.07762835 | 0.08612635 |
| TCGA-E2-A154 | 1.61917808 | 0 | 0.3016534 | 0.1849216 | 0.07541335 | 0 | 0.7753341 | 0.5643035 | 0.6807411 | 0.441983 | 1.091958 | 0.0931748 | 3.622451 | 0 | 0.3486203 |
| TCGA-LL-A7SZ | 1.62739726 | 0 | 0.2548911 | 0.2636804 | 0 | 0.05664246 | 1.228391 | 0.1682912 | 0.08628188 | 0 | 0.2076036 | 0.1968271 | 14.10491 | 0.3894169 | 0 |
| TCGA-E2-A15P | 1.63013699 | 0 | 0.4292699 | 0.1788625 | 0 | 0.07154498 | 0.1034385 | 0.1948543 | 0.9626786 | 0.8962778 | 1.485934 | 0.06629651 | 4.672034 | 0.02981041 | 0.3720798 |
| TCGA-LL-A8F5 | 1.63287671 | 0 | 0.5060944 | 0.2115337 | 0.1150215 | 0 | 1.241677 | 0.1518853 | 0.2491861 | 0 | 0.6495329 | 0.1421115 | 4.222775 | 0 | 0.1063441 |
| TCGA-E2-A1L9 | 1.63835616 | 0 | 0.6768278 | 0.1555926 | 0.06507959 | 0.04627882 | 0.3010911 | 0.2062495 | 0.1409905 | 0.2288513 | 1.074255 | 0.2733845 | 2.746924 | 0.1012349 | 0.3409627 |
| TCGA-A8-A08G | 1.6630137 | 0 | 0.3322676 | 0.05728751 | 0.1661338 | 0.07383724 | 0.05337631 | 0.4021946 | 0.1687113 | 0.1460517 | 0.8118755 | 0.1197361 | 5.69096 | 0.09229654 | 0.106667 |
| TCGA-EW-A1PH | 1.6630137 | 0 | 1.158273 | 0.06086161 | 0.01654675 | 0.05883289 | 0.1275894 | 0.4369981 | 0.08961851 | 0.05818638 | 0.3234478 | 0.06133159 | 7.383337 | 0.09192639 | 0.5354454 |
| TCGA-D8-A1JL | 1.6739726 | 0 | 0.3196641 | 0.4691799 | 0.1229477 | 0.2622885 | 0.5451177 | 0.1298815 | 0.06658947 | 0.2161718 | 0.4272573 | 0.07595227 | 6.348351 | 0.0683043 | 0.3220718 |
| TCGA-BH-A18J | 1.67671233 | 1 | 0.1996837 | 0.06120574 | 0.01664031 | 0 | 0.4170102 | 0.2856549 | 0.1577192 | 0.05851538 | 0.7589789 | 0.1644757 | 2.884431 | 0.03697847 | 0.1538494 |
| TCGA-BH-A2L8 | 1.67671233 | 0 | 1.19453639 | 0.2163563 | 0.03619807 | 0.03217606 | 0.24422796 | 0.16729783 | 0.12253226 | 0.31822481 | 0.35379112 | 0.08944698 | 3.472701757 | 0.02011004 | 0.22311493 |
| TCGA-E9-A243 | 1.67671233 | 0 | 0.4291095 | 0.2724504 | 0.09195203 | 0.01816336 | 0.1969521 | 0.2158617 | 0.05533549 | 0.1077826 | 0.5325728 | 0.1009855 | 5.858161 | 0.07946471 | 0.2361529 |
| TCGA-AC-A8OP | 1.68219178 | 0 | 0.303154 | 0.2206868 | 0.02526283 | 0.4042053 | 0.1460983 | 0.4670323 | 1.676112 | 2.842763 | 2.19478 | 0.06242553 | 4.802839 | 0.1122793 | 0.07785656 |
| TCGA-A7-A13E | 1.68219178 | 1 | 1.6653109 | 1.32002837 | 0.29203371 | 0.1146251 | 1.60778687 | 1.79342377 | 2.4775426 | 1.26472 | 3.08228933 | 0.63113867 | 8.084421333 | 5.38449397 | 1.08551293 |
| TCGA-AO-A0J7 | 1.69315068 | 0 | 0.9430472 | 0.0160587 | 0.05239151 | 0 | 0.03366523 | 0.276731 | 0.8276233 | 0.1842339 | 0.151722 | 0.1294617 | 3.780489 | 0.01940426 | 0.2152845 |
| TCGA-D8-A1JN | 1.69863014 | 0 | 0.3051984 | 0.0935474 | 0 | 0.09042915 | 0 | 0.7724416 | 0.2410594 | 0.2683063 | 1.933383 | 0.1885395 | 5.119653 | 0.02825911 | 0.2743356 |
| TCGA-OL-A5S0 | 1.69863014 | 0 | 0.4423528 | 0.8361201 | 0 | 0.04368916 | 1.563335 | 0.3245137 | 0.133101 | 0 | 0.4270072 | 0.242905 | 5.512294 | 0.2730573 | 0.1893431 |
| TCGA-BH-A0HI | 1.69863014 | 0 | 0.3194032 | 0.1386936 | 0 | 0.031546 | 0.0342065 | 0 | 0.4084517 | 0.06239868 | 0.3854036 | 0 | 3.5266 | 0 | 0.3828054 |
| TCGA-BH-A0AW | 1.70410959 | 0 | 0 | 0.1946982 | 0.09963965 | 0 | 0.2881147 | 0.06578684 | 0.1349141 | 0.0875953 | 0.4869268 | 0.09233018 | 3.01257 | 0.02767768 | 0.03838446 |
| TCGA-AO-A1KO | 1.70410959 | 0 | 1.645983 | 0.2805281 | 0.141895 | 0.1009031 | 0.1641196 | 0.2623204 | 0.4034703 | 0.5987659 | 1.294391 | 0.1753143 | 4.85801 | 0 | 0.2623806 |
| TCGA-A2-A0T4 | 1.70958904 | 0 | 0.33248722 | 0.24022065 | 0.02374909 | 0 | 0.09156275 | 0.25088444 | 0.03215673 | 0.41756637 | 0.46423555 | 0.0880275 | 3.881428439 | 0 | 0.0365957 |
| TCGA-E2-A15L | 1.71506849 | 0 | 0.3479121 | 0.05089623 | 0.09488511 | 0.1124564 | 0.3048518 | 0.08353022 | 0.1284763 | 0.2224413 | 1.202164 | 0.05861628 | 3.25158 | 0 | 0.17058 |
| TCGA-A7-A26J | 1.71780822 | 0 | 2.17169373 | 0.72969397 | 0.3239472 | 0.09223828 | 1.95097367 | 2.5488155 | 2.1300676 | 2.11391417 | 3.62013607 | 0.88606574 | 6.502721333 | 0.73381521 | 0.7966982 |
| TCGA-S3-AA0Z | 1.72328767 | 0 | 0.484714 | 0.1512244 | 0.7616935 | 0.3693059 | 1.034502 | 1.051531 | 0.7500713 | 0.7304953 | 1.879951 | 0.2994372 | 6.360986 | 0.1731122 | 0.480157 |
| TCGA-D8-A1JP | 1.75068493 | 0 | 0.9073812 | 0.109786 | 0.07163536 | 0 | 0.4142768 | 0.2207197 | 0.1293276 | 0 | 0.6223524 | 0.1475117 | 11.70771 | 0 | 0.2207703 |
| TCGA-E2-A155 | 1.75342466 | 0 | 0.5428765 | 0.1185592 | 0.2035787 | 0.1206392 | 0.07848816 | 0.1971381 | 0.3675327 | 0.5727049 | 0.4126844 | 0.03353677 | 6.43104 | 0 | 0.06274014 |
| TCGA-A8-A0A6 | 1.75342466 | 0 | 0.9384922 | 0.3715614 | 0 | 0.09269059 | 0.1005079 | 0.1721215 | 0.2470878 | 0.183344 | 1.18904 | 0 | 5.247685 | 0 | 0.1606835 |
| TCGA-S3-A6ZH | 1.75616438 | 0 | 0.51622128 | 0.11867156 | 0.09679149 | 0 | 0.74634401 | 0.08520846 | 0.13105759 | 0.22691045 | 0.490527 | 0.01993132 | 4.273064775 | 0.0358487 | 0.04971634 |
| TCGA-D8-A146 | 1.76164384 | 0 | 0.5168453 | 0.150499 | 0.1033691 | 0.1531393 | 0.1328438 | 0.2274972 | 0.4432185 | 0.3634956 | 0.8606292 | 0.1490004 | 3.344141 | 0.05742725 | 0.2920217 |
| TCGA-A2-A4S2 | 1.76164384 | 0 | 0.5801468 | 0.3637284 | 0 | 0.1406416 | 0.5083433 | 0.6964373 | 0.7141184 | 0.8345768 | 1.088223 | 0.03258108 | 3.298724 | 0.02930034 | 0.08126965 |
| TCGA-LD-A66U | 1.76986301 | 0 | 0.7230946 | 0.2770478 | 0 | 0.03060718 | 0.7633357 | 0.1818749 | 1.072331 | 0 | 0.4113273 | 0.3403424 | 2.629498 | 0.01912949 | 0.3448835 |
| TCGA-E9-A1RG | 1.77260274 | 0 | 0.7443053 | 0.04752908 | 0 | 0 | 0.2789899 | 0.1638086 | 0.3079401 | 0 | 0.1796212 | 0.1277229 | 3.897621 | 0.02297239 | 0 |
| TCGA-A2-A4RY | 1.77534247 | 0 | 1.367519 | 0.7738394 | 0.1051938 | 0.0935056 | 0.5069581 | 0.6598125 | 1.459953 | 1.572127 | 2.227633 | 0.227446 | 3.772902 | 0.0876615 | 0.1620964 |
| TCGA-E2-A15F | 1.80273973 | 0 | 0.1726424 | 0.1488296 | 0.2373832 | 0.2685548 | 0.04160057 | 0.2849668 | 0.2922015 | 0.7588675 | 1.499879 | 0.1066517 | 6.667805 | 0 | 0.2992837 |
| TCGA-A2-A0YH | 1.80547945 | 0 | 0.1409495 | 0.2187148 | 0.07047477 | 0 | 0.1698187 | 0.4187772 | 0.3578409 | 0.1239117 | 0.2296011 | 0 | 7.369064 | 0.03915265 | 0 |
| TCGA-BH-A0BJ | 1.80821918 | 0 | 0.291882 | 0.243235 | 0 | 0 | 0.1054995 | 0.1927144 | 0.2717096 | 0.1282998 | 0.5150858 | 0 | 2.739581 | 0.02026958 | 0.2529956 |
| TCGA-D8-A1XJ | 1.81917808 | 0 | 0.9572647 | 0.03143726 | 0.09572647 | 0 | 0.1581711 | 0.1083483 | 0.07406602 | 0.1442659 | 0.1188073 | 0.01689601 | 3.593924 | 0.01519468 | 0.1475079 |
| TCGA-A2-A4S3 | 1.82465753 | 0 | 0.4916962 | 0.1255929 | 0.1365823 | 0 | 0.3686076 | 0.0721425 | 0.3698702 | 0.5763472 | 1.008608 | 0.1687503 | 9.369694 | 0 | 0.2525566 |
| TCGA-EW-A2FW | 1.84109589 | 0 | 0.9608263 | 0.05097222 | 0.1108646 | 0.06569752 | 0.4986679 | 0.2683929 | 0.2001504 | 0.06497557 | 0.2809238 | 0.06848777 | 3.285532 | 0.04106095 | 0.1993071 |
| TCGA-AC-A3QP | 1.84931507 | 0 | 0 | 0.3537902 | 0 | 0 | 0.3794645 | 0.2363053 | 0.1696132 | 0 | 0.03886741 | 0.0221099 | 3.625141 | 0.05965067 | 0.303328 |
| TCGA-AC-A2B8 | 1.85479452 | 0 | 0.553069 | 0.2542846 | 0.050279 | 0 | 0.2180776 | 0.2157778 | 0.221256 | 0.5304158 | 1.146634 | 0.1242415 | 3.634748 | 0.06983195 | 0.4648588 |
| TCGA-OL-A5RZ | 1.86027397 | 0 | 0.2357296 | 0.1192196 | 0.04714591 | 0 | 0.04544185 | 0.4046637 | 0.6064467 | 0.08289392 | 0.563191 | 0.2329991 | 2.380127 | 0 | 0.1089729 |
| TCGA-A7-A2KD | 1.86027397 | 0 | 0.5780393 | 0.2088155 | 0.261494 | 0.07340182 | 0.5306156 | 0.308954 | 0.2981625 | 0.3871744 | 0.9864407 | 0.1360343 | 9.206193 | 0.07646023 | 0.1908683 |
| TCGA-AO-A0J8 | 1.8630137 | 0 | 0.8436247 | 0.1026724 | 0.03721874 | 0.02205555 | 0.2391565 | 0.2457358 | 0.3695626 | 0.1308791 | 0.5119681 | 0.1532818 | 3.064212 | 0 | 0.5926322 |
| TCGA-MS-A51U | 1.86575342 | 0 | 0.3073628 | 0.3532905 | 0.05122713 | 0.3642818 | 0.4443799 | 0.1352903 | 3.606852 | 1.531184 | 1.668938 | 0.1582305 | 3.388203 | 0 | 0.3946873 |
| TCGA-EW-A1OW | 1.90136986 | 0 | 0.62639631 | 0.36553631 | 0.33729032 | 0 | 0.65019821 | 0.19088154 | 0.19572761 | 0.0847197 | 0.62792252 | 0.14883191 | 3.564149082 | 0.10707629 | 0.51974111 |
| TCGA-E2-A15C | 1.90136986 | 0 | 1.163576 | 0.1660275 | 0 | 0.07133034 | 0.541423 | 0.3443872 | 1.005064 | 0.6349184 | 0.8278838 | 0.07435981 | 7.932517 | 0.04458146 | 0.2782227 |
| TCGA-BH-A0H7 | 1.92328767 | 0 | 1.06968 | 0.135894 | 0.01407473 | 0 | 0 | 0.1300993 | 0.1334023 | 0.1484807 | 0.275126 | 0.03477926 | 11.37334 | 0.01563859 | 0.08675277 |
| TCGA-EW-A1P5 | 1.9260274 | 0 | 0.2315267 | 0.1153198 | 0 | 0.03430025 | 0.4091235 | 0.1273874 | 0.2351188 | 0.1356933 | 0.1676211 | 0.07151404 | 3.979377 | 0.08575063 | 0.1486528 |
| TCGA-A2-A4S0 | 1.93424658 | 0 | 1.242998 | 0.7330021 | 0 | 0.1921542 | 0.15627 | 0.5709121 | 0.4756427 | 1.140256 | 1.291172 | 0.03338587 | 4.623501 | 0.0600482 | 0.1665542 |
| TCGA-AC-A3YI | 1.9369863 | 0 | 0.46291353 | 0.39374255 | 0 | 0.86410526 | 0.44618172 | 0.36676506 | 0.75215288 | 0.32556556 | 1.50813459 | 0.48614858 | 5.63908649 | 0.07715226 | 0.42799068 |
| TCGA-AR-A0TU | 1.94246575 | 0 | 0.9357255 | 0.5300908 | 0.1002563 | 0.05941114 | 0.4831629 | 0.08825865 | 0 | 0.1175165 | 0.3629188 | 0.0412896 | 2.770938 | 0.07426393 | 0.02574801 |
| TCGA-OL-A66I | 1.95616438 | 0 | 0.9501828 | 0.4938481 | 0.06196844 | 0.1468882 | 1.990954 | 0.2454871 | 0.5593766 | 0 | 0.5383684 | 0.2041689 | 7.375575 | 0.06885382 | 0.1909782 |
| TCGA-A2-A1FV | 1.95616438 | 0 | 3.013458 | 0.2719201 | 0.01408158 | 0.1001357 | 0.1628713 | 0.2231359 | 1.220271 | 0.7427646 | 2.293832 | 0.08699045 | 14.64315 | 0.0156462 | 0.6292635 |
| TCGA-BH-A5J0 | 1.95890411 | 0 | 0.2953706 | 0.2425046 | 0 | 0.07501475 | 0.5693891 | 0.3621757 | 0.4285046 | 0 | 0.183294 | 0.2346021 | 2.322349 | 0.09376844 | 0.06502081 |
| TCGA-A7-A13G | 1.96712329 | 0 | 8.477801 | 1.294737 | 0.6521385 | 1.422848 | 1.828559 | 3.327154 | 11.19815 | 3.439852 | 7.082047 | 4.944258 | 2.997712 | 3.787673 | 1.415996 |
| TCGA-A7-A26G | 1.97808219 | 0 | 0.7577648 | 0.2449671 | 0.05920037 | 0.04209804 | 0.2054182 | 0.1719824 | 0.2885705 | 0.6661668 | 0.591468 | 0.07314332 | 3.741535 | 0.06577819 | 0.8939922 |
| TCGA-E9-A54X | 1.99178082 | 0 | 0.4135118 | 0.03564757 | 0 | 0 | 0.7971311 | 0.1365101 | 0.06998789 | 0.09088171 | 0.3929297 | 0.1915885 | 6.668213 | 0.0287161 | 0.3584211 |
| TCGA-BH-A0W3 | 1.99452055 | 0 | 0.2503841 | 0.04604766 | 0.1001537 | 0.04451273 | 0.2413341 | 0.1653155 | 0.5763426 | 0.2641415 | 1.087641 | 0.06187098 | 3.060112 | 0.1112818 | 0.4629896 |
| TCGA-AC-A3QQ | 2.0109589 | 0 | 6.84698 | 2.453617 | 1.057254 | 0.268509 | 2.232184 | 0.9307328 | 18.67823 | 1.416311 | 1.421518 | 1.430666 | 3.868782 | 1.734121 | 1.551577 |
| TCGA-A7-A26F | 2.02191781 | 0 | 9.693828 | 3.498174 | 2.000761 | 2.003892 | 3.965533 | 4.428133 | 36.0955 | 2.972807 | 4.835183 | 0.7311498 | 4.875671 | 5.886433 | 0.8250374 |
| TCGA-A2-A4RX | 2.03287671 | 0 | 0.47310588 | 0.25830493 | 0.17741471 | 0.42053856 | 0.62700779 | 0.46855053 | 1.1610779 | 0.41591726 | 1.41289539 | 0.07306654 | 4.61532147 | 0.06570915 | 0.18225588 |
| TCGA-BH-A0EB | 2.04109589 | 0 | 0.2829514 | 0.1662294 | 0.01571952 | 0 | 0.06060539 | 0.1245453 | 0.1277073 | 0.2763872 | 0.5121293 | 0.05826541 | 2.845367 | 0.05239841 | 0.1211135 |
| TCGA-C8-A27A | 2.04657534 | 0 | 2.350669 | 0.3218015 | 0.2245021 | 0.07043202 | 0.6873487 | 0.1918231 | 1.251683 | 0.6965805 | 1.319405 | 0.0815815 | 34.93611 | 0.08804003 | 0.5087385 |
| TCGA-E9-A24A | 2.04657534 | 0 | 0.7544549 | 0.2705631 | 0.1056237 | 0.05365013 | 0.1163497 | 0.2789511 | 0.1634475 | 0.3183634 | 1.147045 | 0.1118574 | 5.486616 | 0.03353133 | 0.4185227 |
| TCGA-BH-A0H6 | 2.04657534 | 0 | 0.4463023 | 0.2051964 | 0.0608594 | 0.07212966 | 0.4301709 | 0.3214582 | 0.3296193 | 0.2853481 | 0.5287333 | 0.1002574 | 8.974255 | 0.02254052 | 0.1875603 |
| TCGA-E2-A1LA | 2.04931507 | 0 | 0.7190763 | 0.1264096 | 0.211493 | 0.03759876 | 0.4892369 | 0.3072031 | 0.7159143 | 0 | 0.6430919 | 0.1045217 | 5.024306 | 0.07049768 | 0.1303585 |
| TCGA-A2-A0EX | 2.06027397 | 0 | 0.3553698 | 0.1815427 | 0 | 0 | 0.5328168 | 0.07821069 | 0.1336605 | 0 | 0 | 0.04878522 | 6.626788 | 0 | 0.1521112 |
| TCGA-A2-A0CM | 2.06575342 | 1 | 0.2806756 | 0.5592005 | 0.3040653 | 0.2079079 | 0.2705307 | 0.0926577 | 0.1266801 | 0.3289971 | 1.625633 | 0.08669517 | 5.71347 | 0.4158157 | 0.1441673 |
| TCGA-AC-A3YJ | 2.06575342 | 0 | 0.6197125 | 0.131504 | 0 | 0 | 0.4135245 | 0.03147413 | 0.09681957 | 0.08381572 | 0.4141483 | 0.1177951 | 3.229102 | 0 | 0.1469129 |
| TCGA-E9-A1QZ | 2.06849315 | 0 | 1.032742 | 0.5027548 | 0.1063117 | 0 | 0.2049382 | 0.300823 | 0.4112803 | 0.1068124 | 1.484378 | 0.09382168 | 3.692892 | 0.06749948 | 0.2574298 |
| TCGA-LL-A440 | 2.07945205 | 0 | 0.93669198 | 0.50707092 | 0.04532381 | 0.59088813 | 0.37860849 | 0.61844862 | 0.28639019 | 0.74377527 | 2.75634363 | 0.42932202 | 2.840865036 | 0 | 0.23280285 |
| TCGA-BH-A0W4 | 2.07945205 | 0 | 0.4859286 | 0.1787323 | 0.01619762 | 0.02879577 | 0.09367297 | 0.3208332 | 1.250118 | 0.8543799 | 1.266493 | 0 | 3.335593 | 0 | 0.3494318 |
| TCGA-EW-A6SB | 2.08219178 | 0 | 0.6006869 | 0.5523558 | 0 | 0.05339439 | 1.794824 | 0.1983011 | 0.0813342 | 0 | 0.5870967 | 0.07421615 | 6.386535 | 0.133486 | 0.1851234 |
| TCGA-A8-A07U | 2.08219178 | 0 | 1.024121 | 0.3400657 | 0.2275824 | 0.06743182 | 0.1827971 | 0.6761733 | 0.1540755 | 0.2000724 | 0.4531052 | 0.1640234 | 3.463161 | 0.08428978 | 0.08767219 |
| TCGA-A8-A09Q | 2.08493151 | 0 | 0.4063113 | 0.1601227 | 0.1160889 | 0.05159508 | 0.8112239 | 0.2299423 | 0.3340218 | 0.2551405 | 0.4097257 | 0.03585759 | 9.324208 | 0.08061732 | 0.2236063 |
| TCGA-LL-A50Y | 2.08767123 | 0 | 0.6050568 | 0.1390935 | 0 | 0 | 0.05301702 | 0.3268532 | 0.5585856 | 0 | 0.3584047 | 0.1019401 | 2.592355 | 0.1222337 | 0.04237958 |
| TCGA-E2-A159 | 2.08767123 | 0 | 0.361379 | 0.14122857 | 0.16262055 | 0.06424516 | 0.17415855 | 0.19087969 | 0.26912286 | 0.44477415 | 0.58867167 | 0.42416685 | 4.742851266 | 0.1806895 | 0.16705803 |
| TCGA-D8-A73X | 2.10136986 | 0 | 1.016207 | 0.1668649 | 0.05443967 | 0.03226055 | 0.1049439 | 0.5271737 | 0.4422744 | 0.3828724 | 1.06416 | 0.3587273 | 4.047144 | 0 | 0.4194389 |
| TCGA-A2-A0D4 | 2.10136986 | 0 | 1.035149 | 0.1102766 | 0.08836641 | 0.1346536 | 0.1703449 | 0.133357 | 0.5640636 | 0.3551303 | 0.9596352 | 0.03119388 | 6.524278 | 0 | 0.03890472 |
| TCGA-A2-A0YD | 2.10684932 | 0 | 0.7440458 | 0.2900328 | 0 | 0.2875539 | 0.3118055 | 0.1495122 | 1.33597 | 0.284394 | 0.9134066 | 0.3797044 | 3.03116 | 0.01797212 | 0.3489418 |
| TCGA-BH-A0AY | 2.12876712 | 0 | 0 | 0.2060999 | 0 | 0 | 0.2254251 | 0.4117807 | 0.1319484 | 0.06853584 | 0.4656406 | 0 | 6.05928 | 0 | 0.09009768 |
| TCGA-D8-A1X8 | 2.14520548 | 0 | 0.5309266 | 0.2593607 | 0.01659146 | 0.02949592 | 0.09595059 | 0.2190894 | 0.1572561 | 0.2333743 | 0.4684646 | 0.04099819 | 5.333122 | 0.0368699 | 0.1533977 |
| TCGA-E9-A5UO | 2.15068493 | 0 | 0.8028572 | 0.05828364 | 0.3803008 | 0.1126817 | 0 | 1.088068 | 0.2002524 | 0.2971825 | 1.376654 | 0.3393503 | 5.906929 | 0.04695071 | 0.2604521 |
| TCGA-BH-A1F0 | 2.15068493 | 1 | 0.2850876 | 0.8671099 | 0.08771926 | 0.1949317 | 0.1690974 | 0.2316657 | 0.3117807 | 0.1156737 | 0.7382707 | 0.1490211 | 5.092359 | 0.1340155 | 0.2196501 |
| TCGA-E9-A1NG | 2.15342466 | 1 | 0.3837582 | 0.1764406 | 0 | 0.0487312 | 0.1321027 | 0.09049123 | 0.5567317 | 0.1927828 | 0.6846624 | 0.01693362 | 3.593166 | 0 | 0.5068671 |
| TCGA-EW-A2FV | 2.15890411 | 0 | 0.2773593 | 0.2741712 | 0.1386796 | 0 | 0.2940677 | 0.3662516 | 0.7886548 | 0.2925988 | 0.5120479 | 0.1713416 | 3.812963 | 0.0308177 | 0.0854783 |
| TCGA-EW-A1OV | 2.16164384 | 0 | 0.50979404 | 0.33986269 | 0.0509794 | 0.09063005 | 0.34395742 | 0.60586251 | 0.31062204 | 0.44817058 | 1.43941846 | 0.06298614 | 3.63651383 | 0.05664378 | 0.31422297 |
| TCGA-A8-A06Y | 2.16712329 | 0 | 0.7837286 | 0.1232725 | 0 | 0.3666567 | 0 | 0.1089379 | 0.2234072 | 0.2175765 | 1.030289 | 0 | 4.303228 | 0 | 0.06356167 |
| TCGA-AC-A2FM | 2.16986301 | 1 | 1.994165 | 0.1264631 | 0.1719108 | 0.03056192 | 0.09941828 | 0.9534309 | 0.2560476 | 0.1813564 | 0.2613666 | 0.1061997 | 5.512131 | 0.03820239 | 0.07947079 |
| TCGA-AO-A0J5 | 2.16986301 | 1 | 0.4434574 | 0.1302621 | 0 | 0.1313948 | 0.04749209 | 0.5855834 | 0.6004501 | 0.3898527 | 0.5618465 | 0.09131685 | 2.90866 | 0 | 0.09490796 |
| TCGA-BH-A202 | 2.17808219 | 0 | 0.6487744 | 0.1491436 | 0.1969494 | 0.06178804 | 0.5359927 | 0.06119311 | 0.1568667 | 0.2444362 | 0.5787387 | 0.02862766 | 9.54306 | 0.01287251 | 0.1785208 |
| TCGA-AO-A0JG | 2.18630137 | 0 | 0.4635538 | 0.4475691 | 0 | 0.0549397 | 0.3872256 | 0.3264643 | 1.150712 | 0.3260158 | 1.006814 | 0.07636407 | 4.35705 | 0.03433732 | 0.166671 |
| TCGA-BH-A0HB | 2.20821918 | 0 | 0.3449699 | 0.1486939 | 0.04312124 | 0.03832999 | 0.2078132 | 0.1992949 | 0.2043545 | 0 | 0.09365699 | 0.1065544 | 3.671871 | 0 | 0.09967028 |
| TCGA-E9-A5FK | 2.22465753 | 0 | 0.2717725 | 0.4269223 | 0 | 0 | 0.392924 | 0.2691557 | 1.042625 | 0 | 0 | 0.05596344 | 2.804109 | 0.1258206 | 0.1046957 |
| TCGA-OL-A66H | 2.22465753 | 0 | 0.5489451 | 0.155316 | 0 | 0.1501388 | 0.2035014 | 0.1951598 | 0.08576337 | 0.07424448 | 0.8254239 | 0 | 3.757593 | 0 | 0.1952046 |
| TCGA-A2-A4S1 | 2.24657534 | 0 | 0.7067683 | 0.39264906 | 0.02944868 | 0.05235321 | 0.79475954 | 0.03888684 | 0.51836321 | 0 | 0.70357026 | 0.14553788 | 3.528548699 | 0.03272076 | 0.31764868 |
| TCGA-A8-A0A9 | 2.25205479 | 0 | 0.6910014 | 0.1059006 | 0.100771 | 0.02559265 | 0.2497596 | 0.1520773 | 0.7212145 | 0.5062281 | 1.000545 | 0.05335918 | 4.388187 | 0.09597242 | 0.3327455 |
| TCGA-A2-A0SV | 2.26027397 | 1 | 1.088514 | 0.06103247 | 0.05309825 | 0.02359922 | 0.7165065 | 0.157761 | 0.3954284 | 0 | 0.6054642 | 0.03280201 | 5.170386 | 0.02949903 | 0.08182074 |
| TCGA-BH-A0HX | 2.27123288 | 0 | 0.8746915 | 0.192336 | 0.07606013 | 0.0338045 | 0.07331097 | 0.1757648 | 0.8238954 | 0.06686605 | 0.2064981 | 0.02349348 | 11.76255 | 0 | 0.08790256 |
| TCGA-E2-A108 | 2.29315068 | 0 | 0.5940836 | 0.5365287 | 0.04243455 | 0.2640372 | 0.4090077 | 0.3081904 | 0.143643 | 0.07461019 | 0.5990759 | 0.2359295 | 6.352259 | 0.04714949 | 0.3596379 |
| TCGA-D8-A1XA | 2.29863014 | 0 | 0.2792303 | 0.1100415 | 0.1595602 | 0 | 0.07689647 | 0.342385 | 0.1890417 | 0.420818 | 0.7797511 | 0.07392749 | 4.515162 | 0.08864454 | 0.2151373 |
| TCGA-E9-A6HE | 2.32054795 | 0 | 0.4614831 | 0.1142487 | 0.0354987 | 0 | 0.3763717 | 0.2109414 | 0.4806594 | 0 | 0.1542025 | 0.08771879 | 1.922566 | 0.0197215 | 0.1094021 |
| TCGA-A1-A0SO | 2.33424658 | 0 | 1.241029 | 0.1358543 | 0.1625157 | 0.157591 | 0.1708818 | 0.0390184 | 0.160036 | 0.2078123 | 0.6417732 | 0.164284 | 5.782794 | 0.01641573 | 0.7968083 |
| TCGA-BH-A201 | 2.34520548 | 0 | 0.6605261 | 0.2277676 | 0.1200957 | 0.2935672 | 0.2315097 | 0.1982322 | 0.4065297 | 1.108575 | 0.9129443 | 0.03709518 | 4.635944 | 0.05003986 | 0.4626478 |
| TCGA-OL-A6VO | 2.35068493 | 0 | 0.7215752 | 2.355487 | 0.1082363 | 0.09621003 | 0.4520712 | 0.3573137 | 0.02442568 | 0.1268704 | 0.9795139 | 0.2228804 | 4.193278 | 0.04008751 | 0.05559488 |
| TCGA-B6-A0X4 | 2.35616438 | 1 | 1.603618 | 0 | 0.0801809 | 0.1900584 | 0.1545656 | 0.4940996 | 0.03618884 | 0.8458644 | 0.9868418 | 0.03302173 | 5.700329 | 0.08908989 | 0.3294753 |
| TCGA-E2-A10E | 2.36986301 | 0 | 0.829085 | 0.1058857 | 0.04606028 | 0.1364749 | 0.1183879 | 0.324386 | 0.2910438 | 0.1619702 | 1.500606 | 0.1707254 | 2.798723 | 0 | 0.1892686 |
| TCGA-E2-A1B6 | 2.37534247 | 0 | 2.201408 | 0.5783667 | 0.01123168 | 0.1996742 | 1.277434 | 0.5635922 | 0.167287 | 0 | 0.3903134 | 0.2220316 | 6.901172 | 0.03743892 | 0.4153737 |
| TCGA-E2-A10F | 2.40547945 | 0 | 0.82681012 | 0.2148632 | 0.08987067 | 0.09586204 | 0.10394679 | 0.07120427 | 0.12168666 | 0.18961723 | 0.62462145 | 0.08882969 | 3.388433659 | 0 | 0.16618139 |
| TCGA-OL-A5RX | 2.40547945 | 0 | 1.30225959 | 0.36130865 | 0 | 0.15966401 | 0.64923619 | 0.355785 | 1.12485435 | 1.0264115 | 1.90188013 | 0.24966766 | 3.332378416 | 0 | 0.38057954 |
| TCGA-A8-A06U | 2.41917808 | 1 | 0.6512759 | 0.07045581 | 0.03831035 | 0.03405364 | 0.07385127 | 0.1770602 | 0.4409202 | 0.06735885 | 0.7904759 | 0.04733325 | 8.648231 | 0 | 0.0295168 |
| TCGA-LL-A442 | 2.43561644 | 0 | 0.8425661 | 0.1392171 | 0.01316509 | 0.07021384 | 0.3299204 | 0.5389178 | 0.1960838 | 0.3240639 | 0.6862529 | 0.130126 | 3.865792 | 0.05851153 | 0.040573 |
| TCGA-AC-A4ZE | 2.43835616 | 0 | 1.92952 | 0.6242807 | 0.1250615 | 0.3176165 | 0.8954489 | 0.4246537 | 1.257923 | 1.005204 | 1.707368 | 0.1545161 | 4.421259 | 0 | 0.4680127 |
| TCGA-A7-A13H | 2.4630137 | 0 | 0.74727923 | 0.22644825 | 0.05095086 | 0.0905793 | 0.16369753 | 0.62795003 | 0.4599231 | 0.53750354 | 1.36483742 | 0.16786897 | 3.155578412 | 0.07548275 | 0.18319409 |
| TCGA-A7-A0DC | 2.48219178 | 0 | 11.213959 | 1.02712465 | 0.2783357 | 1.51503601 | 1.51017945 | 2.053687 | 19.410022 | 5.1360309 | 4.449919 | 1.12382376 | 8.665861 | 2.3800569 | 0.285228 |
| TCGA-EW-A1OX | 2.49589041 | 0 | 0.77352029 | 0.11854717 | 0.02148668 | 0.1145956 | 0 | 0.36884966 | 0.3200272 | 0.07555754 | 0.37334313 | 0.02654724 | 5.948901832 | 0 | 0.09932845 |
| TCGA-BH-A0DI | 2.49863014 | 0 | 0.1843258 | 0.3135657 | 0.01843258 | 0.03276903 | 0.2131961 | 0.0730204 | 0.07487424 | 0 | 0.6005185 | 0 | 2.756329 | 0 | 0.1988233 |
| TCGA-A8-A09K | 2.49863014 | 0 | 0.72838 | 0.2363911 | 0.02142294 | 0.03808523 | 0.2064862 | 0.5092 | 0.6091488 | 0.8286676 | 0.7910009 | 0.1852795 | 8.467356 | 0.04760653 | 0.2640902 |
| TCGA-A2-A3XU | 2.49863014 | 1 | 0.1646422 | 0.02270927 | 0.01646422 | 0 | 0.7299798 | 0.04348184 | 0.04458576 | 0 | 0.1072782 | 0.06102568 | 6.162862 | 0.01829358 | 0.1014809 |
| TCGA-E9-A3QA | 2.51506849 | 0 | 0.36833692 | 0.52216344 | 0.19951583 | 0.02728422 | 0.35502353 | 0.48638712 | 0.16624515 | 0.05396878 | 0.63333949 | 0.09481002 | 3.84703359 | 0.08526318 | 0.07094772 |
| TCGA-BH-A18P | 2.52328767 | 1 | 0.5882996 | 0.108193 | 0.1764899 | 0.0348622 | 0.03780238 | 0.1812642 | 0.05310462 | 0.5516655 | 0.979612 | 0.1211428 | 4.235679 | 0.1307332 | 0.2719587 |
| TCGA-BH-A0GY | 2.52876712 | 0 | 0.5663048 | 0.2102989 | 0.08712382 | 0 | 0.1259621 | 0.1725699 | 0.1769511 | 0.07659237 | 0.4730705 | 0.1076433 | 10.902 | 0.04840212 | 0.03356295 |
| TCGA-E9-A3X8 | 2.5369863 | 0 | 0.4315829 | 0.3337214 | 0 | 0 | 0.605067 | 0.3626657 | 0.371873 | 0 | 0 | 0.1454263 | 1.639126 | 0 | 0.1511453 |
| TCGA-A7-A0CJ | 2.55068493 | 0 | 1.21755 | 0.06320246 | 0.1178274 | 0.03491184 | 0.1135686 | 0.1037271 | 0.7445232 | 0.6215074 | 0.7677445 | 0.2183675 | 5.069973 | 0.0436398 | 0.1513033 |
| TCGA-E2-A570 | 2.55068493 | 0 | 1.246881 | 0.09922131 | 0.02397848 | 0.1705137 | 0.554683 | 0.3166347 | 0.194804 | 0 | 1.249919 | 0.2073815 | 2.884601 | 0 | 0.3325427 |
| TCGA-A8-A092 | 2.58082192 | 0 | 0.2617915 | 0.02674753 | 0.2181596 | 0.02585595 | 0.1121463 | 0.4225153 | 0.2756995 | 0.3580054 | 0.6001856 | 0.08984692 | 7.779245 | 0.0484799 | 0.02241125 |
| TCGA-A8-A08P | 2.58356164 | 0 | 0.4187935 | 0.2118036 | 0.3350348 | 0 | 0.08073128 | 0.5806656 | 0.2268219 | 0.07363403 | 0.5912379 | 0.05174283 | 6.392926 | 0.1163315 | 0.3871991 |
| TCGA-BH-A0EE | 2.58356164 | 0 | 0.443563 | 0.0135958 | 0.1774252 | 0 | 0.3990285 | 0.6247709 | 0.260257 | 0.05199273 | 0.417471 | 0.4749606 | 5.470684 | 0.1971391 | 0.20505 |
| TCGA-A8-A08O | 2.58356164 | 0 | 0.04438727 | 0.3061191 | 0.1109682 | 0.03945535 | 0.1711316 | 0.3223724 | 0.3005062 | 0.5463048 | 1.156881 | 0 | 5.598602 | 0.07397878 | 0 |
| TCGA-EW-A6SC | 2.60821918 | 0 | 0.2072939 | 0.1429613 | 0.1036469 | 0.1105567 | 0.07992054 | 0.4105959 | 1.319196 | 0.07289456 | 1.260647 | 0.1024464 | 4.934485 | 0 | 0.1597128 |
| TCGA-A7-A26E | 2.61369863 | 0 | 0.61685013 | 0.63564947 | 0.25794883 | 0.16429042 | 2.8714047 | 2.7913299 | 3.67734963 | 1.26018997 | 2.60808757 | 0.21311585 | 4.850362 | 0.58395468 | 1.43479047 |
| TCGA-BH-A1F2 | 2.62739726 | 1 | 1.196736 | 0.1855405 | 0.1113243 | 0.04947746 | 0.214601 | 0.0183754 | 0.5275735 | 0.04893376 | 0.574252 | 0.05157882 | 5.993616 | 0 | 0.3002003 |
| TCGA-A1-A0SK | 2.64931507 | 1 | 0.6674386 | 1.475891 | 0.158914 | 0 | 0.1225361 | 0.1049226 | 0.5164143 | 0.1117637 | 0.1725762 | 0.2159757 | 12.92511 | 0.2118853 | 0.6366762 |
| TCGA-E2-A14X | 2.6630137 | 0 | 0.5407501 | 0.8107198 | 0.0940435 | 0.188087 | 0.2492719 | 0.217322 | 0.2069222 | 0.206689 | 0.7659652 | 0.1016686 | 12.29561 | 0.07836958 | 0.1086859 |
| TCGA-OL-A5D8 | 2.66575342 | 0 | 0.7167009 | 0.07753356 | 0 | 0 | 0.2844454 | 1.002071 | 0.2854197 | 0.6671294 | 0.6409675 | 0.1562646 | 4.633898 | 0 | 0.2598557 |
| TCGA-BH-A0BC | 2.66849315 | 0 | 0.2669546 | 0.1227378 | 0.07415406 | 0.07909766 | 0.05717903 | 0.2154242 | 0.1204873 | 0.1564569 | 0.7730812 | 0.07329513 | 2.433349 | 0.01647868 | 0.2285326 |
| TCGA-E9-A227 | 2.67123288 | 0 | 0.1763957 | 0.2095121 | 0.04409893 | 0.1306635 | 0.3967132 | 0.3299837 | 0.1990361 | 0.5686015 | 0.7023901 | 0.1089703 | 3.297182 | 0.1306635 | 0.2038602 |
| TCGA-E9-A1RB | 2.6739726 | 1 | 1.03542 | 0.1020118 | 0.2218757 | 0.04382729 | 0.2376179 | 0.1627699 | 0.2169729 | 0.08669135 | 0.7228529 | 0.09137737 | 5.393842 | 0.02739206 | 0.2849126 |
| TCGA-AC-A3BB | 2.70410959 | 0 | 0.8209113 | 0.3446104 | 0.0178459 | 0.03172604 | 0.5160259 | 0.07069626 | 0.4107828 | 0.2510192 | 0.9302477 | 0.1322939 | 2.32362 | 0.05948632 | 0.2474937 |
| TCGA-AQ-A1H3 | 2.70958904 | 0 | 0.538045 | 0.162748 | 0.0707954 | 0.0251717 | 0.08188384 | 0.243061 | 0.2492318 | 0.298741 | 0.7380661 | 0.06997538 | 5.112255 | 0 | 0.2836361 |
| TCGA-A2-A0YC | 2.71232877 | 0 | 0.9820201 | 0.09959636 | 0.01444147 | 0.05134746 | 0.1113559 | 0.3813979 | 0.2346485 | 0.6093984 | 1.035081 | 0.1784275 | 3.843753 | 0.06418432 | 0.2002798 |
| TCGA-B6-A0I6 | 2.71506849 | 1 | 0.26767451 | 0.67687806 | 0.20075588 | 0.11896645 | 0.25799952 | 0.23564193 | 0.12081219 | 0.23531825 | 0.72671811 | 0.0275598 | 3.816666927 | 0.04956935 | 0.06874466 |
| TCGA-BH-A0EA | 2.71506849 | 1 | 0.44096172 | 0.28158475 | 0 | 0.1742071 | 0.14167445 | 0.22644527 | 0.43121785 | 0.08614637 | 1.33020126 | 0.06053529 | 2.75922126 | 0.05443972 | 0.07549906 |
| TCGA-A2-A3XV | 2.72876712 | 0 | 0.5464205 | 0.1847263 | 0 | 0 | 0.2168643 | 0.02122193 | 0.06528213 | 0 | 0 | 0.2581319 | 2.680511 | 0.03571376 | 0 |
| TCGA-LL-A441 | 2.72876712 | 0 | 2.46172502 | 0.71483848 | 0.11336892 | 0.14396053 | 0.81172924 | 1.79643729 | 0.57015679 | 0.56951418 | 2.42713541 | 0.32015932 | 3.397267208 | 0.0539852 | 0.37434359 |
| TCGA-AO-A0J2 | 2.73150685 | 0 | 0.2966213 | 0.1983674 | 0.06741393 | 0.0239694 | 0.4938273 | 0.1068237 | 0.438143 | 0.04741199 | 0.4392582 | 0.2332157 | 4.105552 | 0.02996175 | 0.06232813 |
| TCGA-E9-A1N4 | 2.73972603 | 0 | 0.2094342 | 0.160486 | 0.04654093 | 0.04136972 | 0.4710167 | 0.1075499 | 0.1102804 | 0.6137266 | 0.4801508 | 0.04312673 | 4.754936 | 0.1163523 | 0.2868655 |
| TCGA-E9-A3Q9 | 2.74246575 | 0 | 0.9806979 | 0.2332219 | 0.01690859 | 0.09017912 | 0.2933538 | 0.3572433 | 0.1831565 | 0.5351289 | 1.54243 | 0 | 4.454479 | 0 | 0.2605496 |
| TCGA-E2-A1B4 | 2.75068493 | 1 | 0.6539539 | 0.07516712 | 0 | 0.02422052 | 1.129318 | 0.4317715 | 0.424286 | 0.09581742 | 1.094855 | 0.3366558 | 3.025448 | 0.03027564 | 0.209937 |
| TCGA-A8-A08F | 2.75068493 | 0 | 0.4193695 | 0.1253288 | 0.3774325 | 0 | 0.1616846 | 0.2215101 | 0.2839173 | 0.516147 | 1.001932 | 0.05181399 | 5.949477 | 0 | 0.2907987 |
| TCGA-A8-A091 | 2.75068493 | 0 | 0.7486741 | 0.1408165 | 0.1474661 | 0.1209978 | 0.1312025 | 0.3145611 | 0.3071878 | 0.2792258 | 0.689852 | 0.07007597 | 11.29756 | 0.06301971 | 0.03495925 |
| TCGA-AC-A2QH | 2.75342466 | 0 | 14.19128 | 2.32644 | 1.802991 | 5.169866 | 1.233293 | 1.689629 | 0 | 7.362798 | 2.652773 | 2.371351 | 4.896789 | 1.486336 | 2.867906 |
| TCGA-A8-A09M | 2.75616438 | 0 | 0.7839952 | 0.1720366 | 0.5167241 | 0 | 0.2060885 | 0.3529302 | 0.2653862 | 0.2506278 | 0.8900974 | 0 | 4.600141 | 0.1187871 | 0.08236926 |
| TCGA-A7-A0DB | 2.75890411 | 0 | 1.7268559 | 1.08763037 | 0.27782923 | 0.10841947 | 1.61928656 | 3.04586533 | 3.86996203 | 1.65575223 | 3.31704453 | 1.07401035 | 2.851845333 | 0.29554267 | 1.3745821 |
| TCGA-BH-A1FD | 2.76438356 | 1 | 0.05668303 | 0.07166819 | 0.0991953 | 0.07557737 | 0.05463424 | 0.14969933 | 0.2494373 | 0.09966246 | 0.76945285 | 0.05252481 | 4.801785273 | 0.15745285 | 0.15285311 |
| TCGA-EW-A6SD | 2.76712329 | 0 | 0.9678311 | 0.1156947 | 0.1935662 | 0.03441177 | 0.07462794 | 0.07668098 | 0.5766034 | 0.2042017 | 1.555537 | 0.02391552 | 4.156214 | 0.1290441 | 0.02982722 |
| TCGA-AR-A5QN | 2.77534247 | 0 | 0.800583 | 0.2797439 | 0 | 0.2846517 | 0.3395244 | 0.2748632 | 0.3902419 | 0.1126095 | 0.8694114 | 0.09891373 | 5.17745 | 0.1067444 | 0.1480372 |
| TCGA-D8-A3Z5 | 2.78082192 | 0 | 1.193216 | 0.4488588 | 0.05423711 | 0 | 0.8015765 | 1.074298 | 0.3671906 | 0.1271493 | 1.727734 | 0.4244036 | 3.162703 | 0.02008782 | 0.1114342 |
| TCGA-E2-A1II | 2.80821918 | 0 | 0.73159378 | 0.34930207 | 0.19696756 | 0.05002351 | 1.13908949 | 0.07431277 | 0.15239884 | 0.19789519 | 0.30557346 | 0.03476537 | 4.185191822 | 0.06252938 | 0.17343623 |
| TCGA-AR-A1AY | 2.8109589 | 0 | 0.1843928 | 0.1356453 | 0.2581499 | 0.3605903 | 0.1066368 | 0.09739592 | 0.2247043 | 0.3242071 | 0.7609331 | 0.1139106 | 6.678278 | 0.08195235 | 0.2557229 |
| TCGA-E2-A1IH | 2.8109589 | 0 | 0.6101438 | 0.336631 | 0.01525359 | 0.0271175 | 0.2940452 | 0.1812807 | 0.1032683 | 0.05363901 | 0.5632096 | 0.131923 | 5.407525 | 0.01694844 | 0.2350474 |
| TCGA-BH-A1FH | 2.83287671 | 1 | 0.4628153 | 0.4056284 | 0.05785192 | 0 | 0.05576088 | 0.2482778 | 0.1566653 | 0.5594471 | 0.6910817 | 0.1429545 | 5.272814 | 0.03213995 | 0.2674374 |
| TCGA-E9-A1N8 | 2.84657534 | 0 | 0.1868243 | 2.201091 | 0.04670608 | 0.04151651 | 0.3151253 | 0.215863 | 0.2845843 | 0.2463617 | 0.760823 | 0.02885317 | 2.64147 | 0.07784346 | 0.1439417 |
| TCGA-A2-A0EU | 2.85753425 | 0 | 0.3210167 | 0.1383693 | 0.1203813 | 0.03566853 | 0.1547069 | 0.1324691 | 0.2988308 | 0 | 1.002269 | 0.1239447 | 8.792301 | 0.02229283 | 0.06183308 |
| TCGA-A7-A0CG | 2.85753425 | 0 | 0.4405077 | 0.2784819 | 0 | 0.0326302 | 0.1769107 | 0.2423701 | 0.3230804 | 0.3227163 | 0.9168939 | 0.1360642 | 3.864797 | 0.08157551 | 0.226264 |
| TCGA-E2-A576 | 2.85753425 | 0 | 0.4999194 | 0.172386 | 0 | 0 | 0.4015417 | 0.2475529 | 0.9589427 | 0 | 0.1809663 | 0.1801511 | 3.920153 | 0.1620109 | 0.1604878 |
| TCGA-E2-A107 | 2.86849315 | 0 | 0.5939078 | 0.1847178 | 0.1572109 | 0 | 0 | 0.276795 | 0.1182592 | 0.0614255 | 0.4552713 | 0.02158193 | 2.416724 | 0 | 0.02691679 |
| TCGA-E9-A226 | 2.87123288 | 1 | 1.128168 | 0.08644965 | 0.2393084 | 0.1215535 | 0.06590247 | 0.3160055 | 0.5091872 | 0 | 1.150908 | 0 | 3.555575 | 0 | 0 |
| TCGA-E2-A2P6 | 2.87945205 | 0 | 1.88387741 | 0.14909149 | 0.20074104 | 0.05490353 | 0.56557252 | 0.65249864 | 0.64815593 | 0.10860039 | 1.64337945 | 0.3243336 | 4.270305098 | 0.03431471 | 0.38071148 |
| TCGA-A2-A0D1 | 2.87945205 | 0 | 0.2418858 | 0.08340889 | 0 | 0.1433397 | 0.6994288 | 0.6122006 | 0.6823295 | 0.07088228 | 0.7004837 | 0.1743321 | 6.749777 | 0.04479367 | 0.1242431 |
| TCGA-E2-A573 | 2.90958904 | 0 | 1.00239609 | 0.48967625 | 0.12529951 | 0.37125781 | 0.80513742 | 0.79971077 | 0.28276335 | 0 | 1.04321765 | 0.49023232 | 2.882576867 | 0.09281445 | 0.1287186 |
| TCGA-OL-A5RV | 2.90958904 | 0 | 0.8528724 | 0.3508489 | 0.02244401 | 0.1596019 | 0.5191868 | 0.3260093 | 1.914547 | 1.262784 | 2.973578 | 0.1386503 | 3.019128 | 0 | 0.2420927 |
| TCGA-E9-A1R2 | 2.91232877 | 0 | 1.302162 | 0.2138197 | 0.209276 | 0.08267695 | 0.2241243 | 0.46058 | 0.2203941 | 0.4906105 | 1.01008 | 0.2298355 | 7.255181 | 0.05167309 | 0.1074933 |
| TCGA-A2-A0ET | 2.92054795 | 0 | 1.133187 | 0.1135525 | 0.01452804 | 0 | 0.1960411 | 0.3453158 | 0.1967126 | 0.3576134 | 0.8204072 | 0.1076982 | 8.230286 | 0 | 0.06716014 |
| TCGA-E9-A1NF | 2.9369863 | 1 | 0.3318735 | 0.05449483 | 0.07111576 | 0.1685707 | 0.09139375 | 0.03130267 | 0.674045 | 0.2500774 | 0.3089191 | 0.2050184 | 2.510887 | 0 | 0.07305632 |
| TCGA-A7-A0CE | 2.94246575 | 0 | 0.5859405 | 0.2768812 | 0.04882837 | 0.2025473 | 0.09412698 | 0.2149254 | 0.06611458 | 0 | 0.494912 | 0.201095 | 3.916021 | 0.01808458 | 0.2758842 |
| TCGA-A7-A0CH | 2.95616438 | 0 | 1.155912 | 0.03066079 | 0.1556035 | 0.07903669 | 0.08570244 | 0.3228872 | 0.5417748 | 0.2345045 | 0.6759246 | 0.05492897 | 3.256564 | 0 | 0.3082812 |
| TCGA-A7-A0DA | 2.97260274 | 0 | 0.70932451 | 0.28906642 | 0.20957315 | 0.34391492 | 0.40399644 | 0.48961752 | 0.10914084 | 0.28344636 | 0.52520943 | 0.07967141 | 6.593276474 | 0.19703459 | 0.32293776 |
| TCGA-E9-A1NE | 2.98082192 | 0 | 0.4041413 | 0.5202739 | 0.04041413 | 0.07184734 | 0.311627 | 0.1245222 | 0.5107343 | 0.4737188 | 0.7899957 | 0.1165092 | 7.429367 | 0.07484098 | 0.2075846 |
| TCGA-A2-A3XY | 2.99452055 | 1 | 0.89752124 | 0.22990692 | 0.08975212 | 0.36470704 | 0.76621435 | 0.8126897 | 0.34721757 | 0.63122373 | 1.47594961 | 0.1425737 | 5.899405012 | 0.11397095 | 0.11854443 |
| TCGA-A2-A3KC | 3.01917808 | 0 | 1.12902526 | 0.21628836 | 0.05645126 | 0.1338104 | 0.32646514 | 0.32302236 | 1.222978 | 0.19850994 | 1.96174525 | 0.02324891 | 3.036286935 | 0.06272363 | 0.2609625 |
| TCGA-OL-A5D6 | 3.02465753 | 1 | 0.46911 | 0.1470564 | 0.06396955 | 0.07581576 | 0.3699444 | 0.2815716 | 0.2021041 | 0.2249479 | 0.9262559 | 0.07903573 | 2.531103 | 0.07107727 | 0 |
| TCGA-OL-A5RW | 3.03013699 | 0 | 0.3180534 | 0.1566765 | 0 | 0.04038773 | 2.759017 | 0.3899888 | 2.153253 | 0 | 0.09868495 | 0.3929617 | 2.213965 | 0.2019387 | 0.7001404 |
| TCGA-E9-A1NA | 3.04657534 | 0 | 0.1170524 | 0.1278158 | 0.07315772 | 0 | 0.02820539 | 0.1545671 | 0.1188684 | 0.1029032 | 0.1906735 | 0.01807758 | 3.641889 | 0.03251454 | 0.0450924 |
| TCGA-BH-A28O | 3.06849315 | 0 | 0.91812925 | 0.60786489 | 0 | 0.06528919 | 0.46017081 | 0.72743116 | 0.57185596 | 0.51657382 | 2.4328348 | 0.22687364 | 3.977979489 | 0.02040287 | 0.50931857 |
| TCGA-BH-A0HQ | 3.07123288 | 0 | 0.5606642 | 0.1388028 | 0.10782 | 0 | 0.08313834 | 0.6549289 | 0.2627828 | 0.2274884 | 0.2810151 | 0.05328558 | 3.383062 | 0.02396001 | 0.1993719 |
| TCGA-A8-A08J | 3.08767123 | 1 | 0.4906727 | 0.1057484 | 0.03066704 | 0.02725959 | 0.3842617 | 0.1012392 | 0.2076189 | 0.05392008 | 0.632768 | 0.01894489 | 4.313902 | 0.01703725 | 0.0708837 |
| TCGA-BH-A0BA | 3.10136986 | 0 | 0.2217243 | 0.2463603 | 0.09238513 | 0.1642402 | 0.2493285 | 0.3415835 | 1.100803 | 0.4548191 | 0.6822286 | 0.06848627 | 5.992349 | 0.04106006 | 0.1138873 |
| TCGA-E2-A1BD | 3.10410959 | 0 | 0.4712547 | 0.06842174 | 0.04960576 | 0 | 0.09562556 | 0.6386656 | 0.2182933 | 0.6541419 | 0.5656403 | 0.07661121 | 2.936225 | 0 | 0.324866 |
| TCGA-E2-A1IF | 3.11780822 | 0 | 0.6175567 | 0.1774588 | 0 | 0.1029261 | 0.520831 | 0.3567728 | 1.515583 | 0 | 0.754481 | 0.214595 | 1.769733 | 0.04288588 | 0.1189515 |
| TCGA-E2-A10B | 3.1260274 | 0 | 0.3165733 | 0.1257031 | 0.02877939 | 0.1790718 | 0.1109567 | 0.2850229 | 0.3312269 | 0.1518034 | 0.5625655 | 0.03555755 | 3.082829 | 0.09593131 | 0.08869412 |
| TCGA-BH-A18R | 3.12876712 | 1 | 0.3164195 | 0.1587057 | 0.2301232 | 0.07670775 | 0.1109028 | 0.3038766 | 0.4868616 | 0.1011531 | 0.7184843 | 0.07108054 | 5.627277 | 0.03196156 | 0.1994648 |
| TCGA-E9-A229 | 3.14520548 | 0 | 0.1876395 | 0.1478932 | 0.1876395 | 0.09530895 | 0.3358779 | 0.5663475 | 0.344806 | 0.471308 | 0.9024162 | 0.1821542 | 3.571587 | 0.08935214 | 0.08261129 |
| TCGA-BH-A0B1 | 3.14520548 | 0 | 0.2685815 | 0.2161001 | 0.05035903 | 0 | 0 | 0.02216629 | 0.2500194 | 0.2951445 | 0.6198035 | 0.02073988 | 14.33954 | 0 | 0.1551996 |
| TCGA-AR-A1AS | 3.15068493 | 0 | 1.004563 | 0.08397597 | 0.02283097 | 0 | 0.3080805 | 0.2411854 | 0.06182716 | 0.2408541 | 0 | 0.02820814 | 2.677253 | 0.05073548 | 0.8795236 |
| TCGA-B6-A3ZX | 3.15616438 | 1 | 0.5156649 | 0.6954561 | 0.1375106 | 0.1527896 | 0.5632966 | 0.9306084 | 0.09309606 | 0.7253308 | 1.306662 | 0.2336088 | 3.399664 | 0.1336909 | 0.2118944 |
| TCGA-BH-A0DH | 3.16712329 | 0 | 0.4493019 | 0.1205024 | 0.07488365 | 0.03328162 | 0.6135046 | 0.1236044 | 0.1267424 | 0.06583178 | 0.5285905 | 0.02313009 | 5.524118 | 0.02080102 | 0.02884764 |
| TCGA-A8-A0AD | 3.16986301 | 0 | 0.4394502 | 0.1717392 | 0.1318351 | 0.07812448 | 0.08471329 | 0.02901459 | 0.5057706 | 0.2317979 | 0.1908924 | 0 | 3.829777 | 0 | 0.270865 |
| TCGA-E9-A3HO | 3.17260274 | 0 | 0.3877147 | 0.2406505 | 0.05815721 | 0 | 0.0373701 | 0.5887718 | 0.1049947 | 0 | 1.221038 | 0.5269328 | 4.571279 | 0.04307942 | 0.1194883 |
| TCGA-E2-A14Q | 3.18630137 | 0 | 0.6102614 | 0.342931 | 0.06780682 | 0.06027273 | 0.09803396 | 0.2238464 | 0.3213412 | 0.298052 | 0.6995455 | 0.3560513 | 3.837534 | 0 | 0.1044856 |
| TCGA-A7-A0CD | 3.19178082 | 0 | 0.5462928 | 0.2842177 | 0.01437613 | 0.02555756 | 0.1385651 | 0.07593443 | 0.5061046 | 0.3538739 | 0.2810175 | 0.08881005 | 4.418919 | 0.03194695 | 0.4430524 |
| TCGA-BH-A203 | 3.21643836 | 1 | 1.128904 | 0.1631257 | 0.1290176 | 0.1146823 | 0.1243543 | 0.5536932 | 0.1310193 | 0.4536883 | 1.190932 | 0.2590315 | 13.25088 | 0.03583823 | 0.2982109 |
| TCGA-AR-A5QP | 3.24657534 | 0 | 1.267966 | 0.2817702 | 0.04226554 | 0.03756936 | 0.611068 | 0.3627743 | 0.6867404 | 0.1486261 | 0.6884884 | 0.1566599 | 6.066993 | 0 | 0.3256414 |
| TCGA-E9-A247 | 3.24931507 | 0 | 0.8774737 | 0.0739633 | 0.02924912 | 0 | 0.02819193 | 0.4248566 | 0.5346525 | 0.1542811 | 0.8576214 | 0.1445517 | 4.29435 | 0.04874854 | 0.4281734 |
| TCGA-E9-A22A | 3.25753425 | 0 | 0.5213404 | 0.1141413 | 0.04965146 | 0.08826927 | 0.2632126 | 0.03278226 | 0.1176509 | 0.1745986 | 0.5661615 | 0.0766818 | 6.064271 | 0.02758415 | 0.07650948 |
| TCGA-A1-A0SN | 3.27671233 | 0 | 0.4895782 | 0.1688201 | 0.04589796 | 0 | 0.176956 | 0.1010134 | 0.1657246 | 0 | 0.3322929 | 0.01890263 | 2.737532 | 0.05099773 | 0.1650264 |
| TCGA-AR-A2LO | 3.28219178 | 0 | 1.036069 | 0.3259262 | 0.08179494 | 0 | 0.157677 | 0.3240295 | 0.6275945 | 0.2876306 | 1.006707 | 0.1684323 | 4.882923 | 0 | 0.1260404 |
| TCGA-E9-A1NC | 3.29589041 | 0 | 0.2834236 | 0.4002369 | 0 | 0.0359903 | 0.2341537 | 0.08019846 | 0.08223453 | 0 | 0.3077903 | 0.1250628 | 5.349491 | 0.1124697 | 0.1247818 |
| TCGA-A2-A3KD | 3.30410959 | 0 | 0.6909643 | 0.09668666 | 0.10514674 | 0.05340787 | 0.20269251 | 0.05950533 | 0.30508021 | 0.36974678 | 0.78299318 | 0.12991103 | 3.892401713 | 0.06675984 | 0.27775521 |
| TCGA-EW-A1IX | 3.30958904 | 0 | 0.497397 | 0.2858603 | 0.01776418 | 0.09474229 | 0.1027326 | 0.4691503 | 0.4570079 | 0.3748047 | 0.5401596 | 0.1755842 | 2.775575 | 0.03947595 | 0.3558539 |
| TCGA-A8-A08Z | 3.33424658 | 0 | 0.7973569 | 0.2367631 | 0.1328928 | 0.3248491 | 0 | 0.4387108 | 0.2249244 | 0.8762164 | 0.649431 | 0.1231439 | 4.209047 | 0.05537201 | 0.3327653 |
| TCGA-OL-A5RU | 3.33972603 | 0 | 0.9085433 | 0.3446199 | 0 | 0.0807594 | 0.3502817 | 0.5998635 | 2.306598 | 0.4792316 | 0.9866533 | 0.1964418 | 2.844924 | 0 | 0.2100004 |
| TCGA-E2-A10C | 3.34246575 | 0 | 0.4873698 | 0.112039 | 0.02215317 | 0.1181503 | 0.3843442 | 0.6728226 | 0.0899875 | 0 | 1.154772 | 0.2189657 | 5.068133 | 0 | 0.06827302 |
| TCGA-OL-A6VR | 3.34246575 | 0 | 0.77038 | 0.309923 | 0 | 0.02853259 | 0.1856337 | 0.105967 | 0.4780919 | 0.2257524 | 0.8714705 | 0.1189776 | 7.628159 | 0 | 0.2225817 |
| TCGA-EW-A1OZ | 3.36712329 | 0 | 0.2437018 | 0.04902048 | 0 | 0.05415596 | 0.6753183 | 0.06033883 | 0.2062357 | 0.05356084 | 0.4962254 | 0.01881867 | 2.411468 | 0 | 0.1173524 |
| TCGA-AO-A12H | 3.38082192 | 0 | 0.4444785 | 0.102179 | 0 | 0 | 0.228487 | 0.1173864 | 1.624948 | 0.4689004 | 0.4505122 | 0.09152711 | 3.690156 | 0 | 0.1826429 |
| TCGA-AC-A2FB | 3.38082192 | 0 | 0.55618554 | 0.40488602 | 0.0154496 | 0.13732976 | 0.14891179 | 0.26521456 | 0.54389559 | 0.27164129 | 0.50333533 | 0.07635323 | 3.966451023 | 0.05149866 | 0.23806765 |
| TCGA-E2-A14P | 3.41369863 | 0 | 0.423195 | 0.1591956 | 0.09618068 | 0.03419758 | 0.259572 | 0.1270061 | 0.5730144 | 0.3382178 | 1.128056 | 0.09506662 | 3.985904 | 0.02137349 | 0.1778494 |
| TCGA-E9-A22D | 3.41917808 | 0 | 0.2499096 | 0.2585272 | 0.08746836 | 0.02221419 | 1.059858 | 0.148502 | 0.2199486 | 0.04394015 | 0.4070926 | 0.1543843 | 3.555379 | 0 | 0.115528 |
| TCGA-EW-A1P0 | 3.42739726 | 0 | 0.9182976 | 0.1583272 | 0.06887232 | 0 | 0.08851061 | 0.06063038 | 0.3108483 | 0.1614589 | 0.3988985 | 0 | 14.15361 | 0 | 0.1768792 |
| TCGA-E9-A1ND | 3.46849315 | 0 | 0.92333396 | 0.27593889 | 0.11541675 | 0 | 0.04449802 | 0.18288871 | 0.21878717 | 0.08117222 | 0.55149359 | 0.14259984 | 2.120487385 | 0 | 0.0711397 |
| TCGA-E9-A22E | 3.47671233 | 0 | 0.2618396 | 0.1136979 | 0.02909329 | 0.0258607 | 0.1962921 | 0.09604388 | 0.2954467 | 0.2557652 | 0.5055124 | 0.05391807 | 3.929846 | 0.04848882 | 0.179323 |
| TCGA-BH-A0C0 | 3.47945205 | 0 | 0.5535559 | 0.1908813 | 0.04612966 | 0 | 0.1778493 | 0.243656 | 0.0936907 | 0 | 0.5510511 | 0.05699417 | 8.202466 | 0.05125518 | 0.07108262 |
| TCGA-AR-A1AT | 3.48493151 | 1 | 0.23923493 | 0.20898684 | 0.09569397 | 0 | 0.04611758 | 0.12636343 | 0 | 0.16825314 | 0.41568423 | 0.05911597 | 3.122595131 | 0.02658166 | 0.03686445 |
| TCGA-OL-A66K | 3.49315068 | 1 | 0.8284204 | 0.1503485 | 0 | 0.03875651 | 0.7144272 | 0.287875 | 1.948212 | 0.4599674 | 1.609886 | 0.1616102 | 4.395371 | 0.02422282 | 0.1007794 |
| TCGA-E9-A228 | 3.52054795 | 0 | 0.6347066 | 0.3742897 | 0.1793736 | 0.1717081 | 0.07979549 | 0.1275411 | 0.5604817 | 0.09704066 | 0.479495 | 0.1363815 | 5.69168 | 0 | 0.1275703 |
| TCGA-BH-A1EU | 3.52328767 | 1 | 0.6031366 | 0.3872696 | 0.04159563 | 0.2773042 | 0.1603687 | 0.2883658 | 0.8870605 | 0.6582165 | 1.69394 | 0.1670249 | 7.015809 | 0.0577717 | 0.3525279 |
| TCGA-BH-A0W5 | 3.52876712 | 0 | 0.8841101 | 0.5690824 | 0.0176822 | 0.1571751 | 0.1363447 | 0.6771286 | 0.1436523 | 0.5596126 | 1.958644 | 0.3713945 | 6.15549 | 0.1571751 | 0.2452235 |
| TCGA-OL-A66L | 3.56438356 | 0 | 0.7545458 | 0.3565542 | 0 | 0.07452304 | 1.898991 | 0.3044476 | 3.263668 | 0 | 0.2731388 | 0.3884406 | 2.675463 | 0.02328845 | 0.1937838 |
| TCGA-A8-A07B | 3.58356164 | 0 | 0.4474106 | 0.08571085 | 0.1118527 | 0.5965475 | 0.2874928 | 0.7385045 | 0.4038686 | 0 | 1.052731 | 0.3224581 | 14.45652 | 0 | 0.8043337 |
| TCGA-E2-A105 | 3.58356164 | 0 | 0.7516504 | 0.1277167 | 0.06536091 | 0.1452465 | 0.6614839 | 0.7767789 | 0.3761248 | 0.1723804 | 1.561564 | 0.201887 | 6.925711 | 0.01815581 | 0.2517916 |
| TCGA-A8-A08X | 3.58356164 | 0 | 0.158746 | 0.09731554 | 0.2645766 | 0 | 0 | 0.2445605 | 0.6090113 | 0.5582276 | 0.6895752 | 0.261512 | 6.031766 | 0.146987 | 0.04076943 |
| TCGA-D8-A1XW | 3.58630137 | 0 | 0.7494 | 0.3047958 | 0.1008808 | 0.2049641 | 0.2500315 | 0.361577 | 0.2731891 | 0.5574573 | 0.5008173 | 0.05341729 | 12.9947 | 0.06405128 | 0.1776575 |
| TCGA-BH-A0BF | 3.62739726 | 1 | 0.72980984 | 0.35232199 | 0.2371882 | 0.12974397 | 0.56274494 | 0.28911311 | 0.19763539 | 0.89822749 | 1.42659661 | 0.04508478 | 3.11016314 | 0.0202725 | 0.25303198 |
| TCGA-E2-A14O | 3.72328767 | 0 | 0.7590443 | 0.03965749 | 0 | 0 | 0.06650991 | 0.2050188 | 0.4438059 | 0.1819886 | 0.5620238 | 0.04262797 | 2.560349 | 0 | 0.2126609 |
| TCGA-BH-A0E7 | 3.73424658 | 0 | 0.892545 | 0.2120222 | 0.119006 | 0.05289156 | 0.1147046 | 0.6089441 | 0.2618468 | 0.8369653 | 0.6784958 | 0.1654137 | 8.042263 | 0.03305722 | 0.06876751 |
| TCGA-A2-A0SW | 3.73972603 | 1 | 0.3021362 | 0.2778264 | 0.3452985 | 0.1150995 | 0.8320445 | 0.1424891 | 0.4967623 | 0.3794489 | 0.6562234 | 0.05332795 | 3.899477 | 0.07193718 | 0.06651014 |
| TCGA-E9-A1RA | 3.75068493 | 0 | 0.6033367 | 0.1600363 | 0.1160263 | 0.04125379 | 0.4473303 | 0.4902789 | 0.1256815 | 0.3264036 | 0.7560084 | 0.1146824 | 3.699866 | 0.1031345 | 0.1072731 |
| TCGA-A8-A09I | 3.75616438 | 0 | 0.4825286 | 0.1331113 | 0.1833609 | 0.03431315 | 1.320849 | 0.08920474 | 0.5096156 | 0.1357443 | 0.8174603 | 0.1192349 | 3.317908 | 0.01072286 | 0.07435433 |
| TCGA-BH-A0C1 | 3.86575342 | 1 | 0.2679154 | 0.1385769 | 0.05023413 | 0.05953675 | 0.2259527 | 0.2432244 | 1.904504 | 0 | 0.6182662 | 0.08275378 | 3.865579 | 0.0558157 | 0.1290122 |
| TCGA-PE-A5DC | 3.91780822 | 1 | 1.683162 | 0.1793108 | 0.1026319 | 0 | 0.2769824 | 0.2168398 | 0.9171727 | 0.07218065 | 0.534986 | 0.2536077 | 5.401008 | 0 | 0.2530378 |
| TCGA-A1-A0SH | 3.9369863 | 0 | 0.4404988 | 0.1012641 | 0.02936659 | 0.02610363 | 0.198136 | 0.09694609 | 0.03976294 | 0.1032671 | 0.1275653 | 0.03628304 | 2.692988 | 0.03262954 | 0.1583816 |
| TCGA-A2-A3XX | 3.94246575 | 1 | 0.4581006 | 0.3309758 | 0.09816441 | 0.0581715 | 0.3784652 | 0.3456683 | 0.1329165 | 0.230129 | 0.9949696 | 0.0606421 | 7.438866 | 0.2181431 | 0.2521076 |
| TCGA-D8-A27K | 4.00273973 | 0 | 0.52348507 | 0.17299076 | 0.06543563 | 0.0290825 | 0.12614098 | 0.30242604 | 0.13290171 | 0.46020666 | 0.78167454 | 0.10105889 | 3.180635707 | 0.03635313 | 0.25207949 |
| TCGA-A1-A0SF | 4.00821918 | 0 | 0.7478909 | 0.2960998 | 0.08309899 | 0 | 0 | 0.4114943 | 0.5063295 | 0.4383243 | 0.8573108 | 0.05133528 | 3.342214 | 0 | 0.1600623 |
| TCGA-E9-A1R7 | 4.01917808 | 0 | 0.1987444 | 0.083762 | 0.1490583 | 0 | 0.03192681 | 0.06561025 | 0.08970128 | 0.1164802 | 0.4676339 | 0.06138823 | 4.540461 | 0.01840226 | 0.02552095 |
| TCGA-A2-A0CO | 4.02191781 | 0 | 1.15967284 | 0.73542471 | 0 | 0.42654633 | 0.77086686 | 0.73926874 | 0.9475465 | 0.98433768 | 1.21594655 | 0.19762764 | 3.48179356 | 0.08886382 | 0.27728935 |
| TCGA-A2-A0YL | 4.03835616 | 0 | 0.4998185 | 0.283422 | 0.04998185 | 0.1184755 | 0.0642337 | 0.08800106 | 0.2481468 | 0.2929339 | 0.5427893 | 0.08233818 | 4.330596 | 0.03702359 | 0.3850929 |
| TCGA-3C-AALJ | 4.03835616 | 0 | 0.8146107 | 0.2288817 | 0.04525615 | 0.2413661 | 0.5234446 | 0.2390421 | 0.9191658 | 0.1591425 | 0.7863512 | 0.6150643 | 3.362014 | 0 | 0.0697366 |
| TCGA-C8-A12Y | 4.04383562 | 0 | 0.4940418 | 0.09464402 | 0 | 0.07319138 | 1.296281 | 0.1268516 | 0.4087981 | 0 | 0.4172902 | 0.2204219 | 2.148604 | 0.04574461 | 0.1903211 |
| TCGA-A8-A09E | 4.08767123 | 0 | 0.6270622 | 0.03603806 | 0.07838277 | 0.06967357 | 0.1133245 | 0.2328842 | 0.07959887 | 0.2756317 | 0.3404862 | 0.02421089 | 3.356019 | 0.04354598 | 0.1207824 |
| TCGA-A2-A0YI | 4.12328767 | 0 | 0.9891187 | 0.2340714 | 0.01454586 | 0.2327338 | 0.2243217 | 0.1152465 | 0.4136031 | 0.5115029 | 1.105749 | 0.1078303 | 3.699573 | 0.04848621 | 0.2017275 |
| TCGA-BH-A1EX | 4.13150685 | 1 | 0.609539 | 0.06467257 | 0 | 0 | 0.4293324 | 0.216702 | 0.206332 | 0.3709789 | 0.7128615 | 0.05793064 | 4.262255 | 0.07814602 | 0.4335035 |
| TCGA-A2-A0T3 | 4.15342466 | 0 | 0.2277455 | 0.1047106 | 0.3090832 | 0 | 0.3449502 | 0.1718497 | 0.2202657 | 0.5148408 | 0.8126408 | 0.1004944 | 4.743007 | 0 | 0.05013431 |
| TCGA-BH-A0BV | 4.16164384 | 0 | 0.3108748 | 0.1786637 | 0 | 0 | 0.2621836 | 0.07697037 | 0.1315408 | 0.06832413 | 0.3376016 | 0.02400578 | 1.82831 | 0 | 0.1197591 |
| TCGA-A8-A09D | 4.16986301 | 0 | 0.663605 | 0.1779783 | 0.1106008 | 0.06554123 | 0.1421376 | 0.2677544 | 0.4991857 | 0.259284 | 0.6806205 | 0.09109978 | 7.642114 | 0.02048163 | 0.1420235 |
| TCGA-E2-A1LG | 4.17260274 | 0 | 0.4478844 | 0.03861072 | 0.1399639 | 0 | 0.2698099 | 0.2033038 | 0.8717637 | 0.09843613 | 0.4559909 | 0.7781775 | 6.862492 | 0.04665462 | 0.5391867 |
| TCGA-A2-A0SX | 4.20273973 | 0 | 1.753386 | 0.6046159 | 0.1785856 | 0.2020363 | 0.4694474 | 1.350614 | 0.1978433 | 0.7992644 | 1.445728 | 0.3209401 | 4.279636 | 0.1262727 | 0.8255644 |
| TCGA-AO-A0JB | 4.22465753 | 0 | 0.6890639 | 0.1900866 | 0 | 0 | 0 | 0.09099056 | 0.2332515 | 0 | 0.2993219 | 0.5108119 | 1.370559 | 0.03828133 | 0.15927 |
| TCGA-BH-A0HY | 4.23287671 | 0 | 0.43185095 | 0.06872959 | 0.23253512 | 0.11811308 | 0 | 0.21932965 | 0.29236735 | 0.64248323 | 0.79365576 | 0.04104316 | 3.943195997 | 0.01845517 | 0.2815376 |
| TCGA-A2-A3Y0 | 4.23561644 | 0 | 0.7135493 | 0.1212428 | 0.1240955 | 0.05515357 | 0.7176609 | 0.1229007 | 0.7351217 | 0.05454749 | 0.6401308 | 0.09582667 | 4.720959 | 0.08617746 | 0.09561133 |
| TCGA-AO-A0JC | 4.23835616 | 0 | 1.84000722 | 2.33605933 | 0.16727338 | 0.07434373 | 1.65258042 | 1.76706959 | 0.28311433 | 1.39700848 | 2.49774736 | 0.72334436 | 4.663277963 | 0.1626269 | 0.16109796 |
| TCGA-AR-A24K | 4.24109589 | 0 | 0.44977 | 0.07108434 | 0.2389403 | 0.07496167 | 0.2167566 | 0.4083194 | 0.09515586 | 0.4942528 | 0.9158213 | 0.03473128 | 3.687081 | 0.04685104 | 0.4115071 |
| TCGA-BH-A18V | 4.2630137 | 1 | 0.33159048 | 0.870099 | 0.15013004 | 0.21306275 | 0.26021962 | 0.28806417 | 0.40758328 | 0.24184842 | 0.8522455 | 0.26477843 | 5.538049842 | 0.17735819 | 0.19338159 |
| TCGA-BH-A0HW | 4.27671233 | 0 | 1.199276 | 0.1748314 | 0.05850126 | 0.1820039 | 0.592061 | 0.2317519 | 0.3960593 | 0.4628671 | 0.4447155 | 0.1264892 | 5.53995 | 0.0650014 | 0.06760981 |
| TCGA-E2-A15T | 4.28219178 | 0 | 0.3245139 | 0.06714082 | 0.1298056 | 0.02884568 | 0.06255691 | 0.3213893 | 0.4613682 | 0.3423444 | 0.5638613 | 0.02004719 | 4.78006 | 0 | 0.1000107 |
| TCGA-BH-A0B8 | 4.29863014 | 0 | 0.3531264 | 0.08117848 | 0 | 0.03923627 | 0.04254535 | 0.204007 | 0.4482564 | 0.1552204 | 0.1438071 | 0 | 5.715605 | 0.04904533 | 0.2040538 |
| TCGA-BH-A0B9 | 4.30684932 | 0 | 0.3815653 | 0.9453853 | 0.2119807 | 0.3956974 | 0.2656144 | 0.3918873 | 0.1578644 | 0.07454267 | 0.8287391 | 0.2095254 | 4.472021 | 0.03533012 | 0.3756448 |
| TCGA-E2-A1LS | 4.39452055 | 0 | 0.959201 | 0.07579893 | 0.04496255 | 0 | 0.1733496 | 0.09895471 | 2.029339 | 0.3162201 | 0.4231769 | 0.4259001 | 4.317123 | 0.04995839 | 0.4157051 |
| TCGA-BH-A0HA | 4.41369863 | 0 | 0.2852224 | 0.1686043 | 0 | 0 | 0.5105529 | 0.2690248 | 0.6068805 | 0.1432828 | 0.1327473 | 0.1510278 | 2.747053 | 0.0226367 | 0.1883606 |
| TCGA-EW-A1P3 | 4.41369863 | 0 | 0.7218889 | 0.2489272 | 0.02578175 | 0.1833369 | 0.2484988 | 0.4425807 | 0.4538169 | 0.5439665 | 2.127869 | 0.2866851 | 2.953252 | 0.05729277 | 0.357551 |
| TCGA-WT-AB41 | 4.41369863 | 0 | 4.218888 | 0.3452041 | 0 | 0 | 0.482454 | 0.2360602 | 0.09682131 | 0 | 0.3106168 | 0.3975655 | 1.770707 | 0.03972587 | 0.2203734 |
| TCGA-AO-A0J9 | 4.41917808 | 0 | 0.757314 | 0.3080145 | 0.02912746 | 0.1812375 | 0.168448 | 0.3077014 | 0 | 0.1536394 | 1.043844 | 0.1619442 | 1.789238 | 0 | 0.1795336 |
| TCGA-A2-A0CZ | 4.42739726 | 0 | 0.7861362 | 0.2349373 | 0 | 0.1397575 | 0.4167469 | 0.1816656 | 0.266111 | 0.2764435 | 0.341489 | 0.0242822 | 8.926551 | 0.02183712 | 0.2119918 |
| TCGA-BH-A0H5 | 4.43835616 | 0 | 0.5086702 | 0.6656339 | 0.01956424 | 0 | 0.5279988 | 0.7492001 | 0.02649037 | 0.1375947 | 0.6798795 | 0.0725161 | 4.222672 | 0 | 0.3316186 |
| TCGA-E2-A1B0 | 4.46849315 | 0 | 0.3205543 | 0.1678509 | 0.2226071 | 0.1266387 | 0.01716489 | 0.2704355 | 0.6269421 | 0.3131177 | 0.5415095 | 0.1100143 | 9.32056 | 0.0395746 | 0.06860445 |
| TCGA-AO-A12G | 4.49041096 | 0 | 0.26968854 | 0.1317444 | 0 | 0.05993079 | 0.0324926 | 0.0445153 | 0.15975908 | 0 | 0.10982791 | 0 | 8.813685305 | 0 | 0.41557142 |
| TCGA-E2-A15J | 4.49315068 | 0 | 0.2713991 | 0.07798825 | 0.05088733 | 0.06031091 | 0.3269869 | 0.2015894 | 0.3674797 | 0.3578889 | 0.9210376 | 0.08382984 | 3.777637 | 0.03769432 | 0.1829657 |
| TCGA-BH-A1FR | 4.49863014 | 1 | 0.9546138 | 0.2656517 | 0.05024283 | 0.08932059 | 0.09685365 | 0.2322089 | 0.2721191 | 0.1766781 | 0.7093105 | 0.09311412 | 5.141173 | 0 | 0.425814 |
| TCGA-BH-A0DO | 4.50410959 | 0 | 0.9089684 | 0.2674666 | 0.01817937 | 0.09695663 | 0.4205348 | 0.3360808 | 0.8861481 | 0.5114196 | 0.7107228 | 0.3144539 | 3.505541 | 0 | 0.2521184 |
| TCGA-E2-A1L6 | 4.51506849 | 0 | 0.7566567 | 0.2464208 | 0.1103458 | 0.2802432 | 0.2127147 | 0.6036601 | 0.3415094 | 1.274953 | 2.807505 | 0.2337164 | 4.608693 | 0.0175152 | 0.5101057 |
| TCGA-A2-A0SU | 4.55342466 | 0 | 0.37184653 | 0.12347394 | 0.02065814 | 0.03672558 | 0.11946876 | 0.08183693 | 0.16782918 | 0.14528802 | 0.40381523 | 0.05104714 | 3.27724946 | 0 | 0.15916384 |
| TCGA-A2-A0CY | 4.58356164 | 0 | 1.064744 | 0.117489 | 0 | 0 | 0.1231511 | 0.6186355 | 0.2306696 | 0.1497662 | 0.416262 | 0.2631028 | 1.918772 | 0 | 0.4593953 |
| TCGA-EW-A2FR | 4.58356164 | 0 | 0.1811633 | 0.1368393 | 0.02588047 | 0.2300486 | 0.0748351 | 0.2563128 | 0.07008534 | 0.04550413 | 0.4777933 | 0.1119156 | 2.774366 | 0.01437804 | 0.1595201 |
| TCGA-BH-A1FL | 4.58356164 | 1 | 0.3882068 | 0.1441617 | 0.1194483 | 0.07963217 | 0.2302617 | 0.4140435 | 0.5054228 | 0.8400756 | 1.524181 | 0.2582665 | 4.155344 | 0.04977011 | 0.3451153 |
| TCGA-AO-A0JL | 4.6109589 | 0 | 0.9374632 | 0.09236091 | 0.02232055 | 0.1587239 | 0.2151379 | 0.2652676 | 0.6044494 | 0.3139594 | 0.5332693 | 0.1654651 | 6.315279 | 0.1984049 | 0.1375777 |
| TCGA-A2-A04X | 4.61917808 | 0 | 0.7342499 | 0.1687931 | 0.1596196 | 0.05675362 | 0.8307909 | 0.1686215 | 0.4322561 | 0.3929097 | 0.554696 | 0.07888534 | 2.226227 | 0.141884 | 0.1475776 |
| TCGA-BH-A1FU | 4.62465753 | 1 | 0.1255709 | 0.3367802 | 0.06278545 | 0.22323716 | 0.1210322 | 0.19345174 | 0.31171338 | 0.58875735 | 0.40909977 | 0.05171517 | 6.4563798 | 0.09301548 | 0.03224935 |
| TCGA-D8-A1JB | 4.62465753 | 0 | 0.6150277 | 0.4132812 | 0.04730982 | 0.08410635 | 0.04559983 | 0.3435981 | 0.2562337 | 0.6654568 | 0.7706577 | 0.05845229 | 6.726828 | 0 | 0.4009564 |
| TCGA-BH-A18Q | 4.63561644 | 1 | 2.369836 | 1.410617 | 0.1904333 | 0.2256987 | 0.7342005 | 0.4191103 | 0.4584007 | 0.5208431 | 0.6893511 | 0.1568562 | 4.830605 | 0.07053084 | 0.1956297 |
| TCGA-E2-A15I | 4.63561644 | 0 | 0.7751333 | 0.2036475 | 0.05536666 | 0.03280987 | 0.2134618 | 1.121042 | 0.9245998 | 1.362872 | 2.685659 | 0.1824178 | 5.071361 | 0.08202468 | 0.2559486 |
| TCGA-BH-A1EW | 4.64109589 | 1 | 0.3782502 | 0.1391265 | 0.2458626 | 0.1344889 | 0.1822892 | 0.1748171 | 0.3072949 | 0.2660221 | 0.8215388 | 0.116834 | 6.447815 | 0.1050695 | 0.05828574 |
| TCGA-AR-A0TY | 4.65479452 | 1 | 0.3580902 | 0.1097594 | 0.1193634 | 0.05305039 | 0.05752452 | 0.05910704 | 0.8889121 | 0.1574023 | 0.3240635 | 0.073738 | 3.229903 | 0.01657825 | 0.1379481 |
| TCGA-A2-A3XW | 4.69041096 | 0 | 0.3750478 | 0.6466342 | 0 | 0.05556264 | 1.174849 | 0.4127074 | 0.2539111 | 0.3297124 | 0.1697049 | 0.1930748 | 1.338164 | 0 | 0.07224035 |
| TCGA-A2-A0CX | 4.73424658 | 0 | 0.1786708 | 0.07393276 | 0.2501392 | 0.1588185 | 0.06888514 | 0.2123406 | 0.3870781 | 0.1884879 | 0.232838 | 0.02207516 | 3.56521 | 0 | 0.05506389 |
| TCGA-D8-A13Y | 4.73424658 | 0 | 1.078035 | 0.2478241 | 0.6352704 | 0.03422332 | 1.595714 | 0.1016814 | 1.902797 | 0.135389 | 0.7944147 | 0.07135364 | 8.414529 | 0.06416873 | 0.711933 |
| TCGA-E2-A15R | 4.74520548 | 0 | 0.7502263 | 0.161339 | 0.1573055 | 0.08604746 | 0.06997835 | 0.1917426 | 0.4915264 | 0.3829585 | 0.4467849 | 0.01495033 | 7.222316 | 0 | 0.07458367 |
| TCGA-AR-A2LR | 4.77260274 | 0 | 0.84126178 | 0.65538912 | 0.65431472 | 0.66470066 | 0 | 0.15428918 | 0.37969502 | 0.4930472 | 1.2181166 | 0.14436067 | 7.179215164 | 0.05192974 | 0.6121541 |
| TCGA-BH-A208 | 4.81917808 | 1 | 0.0960149 | 0.1324343 | 0 | 0.02844886 | 0.09254448 | 0.211312 | 0.1950091 | 0.2250899 | 0.3128087 | 0.1383999 | 2.628146 | 0 | 0.1479523 |
| TCGA-OL-A5D7 | 4.87671233 | 0 | 1.729982 | 0.8285356 | 0.09611013 | 0.08543123 | 0.5558176 | 0.4441954 | 0.09760126 | 0.08449243 | 0.730611 | 0.3859249 | 2.713924 | 0.1868808 | 0.07404954 |
| TCGA-B6-A0X7 | 4.87945205 | 1 | 0.6292437 | 0.2816941 | 0.03311809 | 0.08831491 | 0.1915263 | 0.5029212 | 0.739902 | 0.2329184 | 2.409678 | 0.1636724 | 6.015819 | 0.01839894 | 0.2551635 |
| TCGA-AO-A03U | 4.91232877 | 1 | 0.3071821 | 0.3471982 | 0.06399627 | 0.06826269 | 0.1973861 | 0.06760541 | 0.745209 | 0 | 0.3613907 | 0.04744125 | 3.551841 | 0.01422139 | 0.0591683 |
| TCGA-E2-A1IK | 4.93150685 | 0 | 0.3236108 | 0.1735843 | 0 | 0.09588469 | 0.1039713 | 0.1424422 | 0.1460585 | 0 | 0.4685768 | 0.033319 | 4.347124 | 0.02996396 | 0.08311032 |
| TCGA-GM-A2D9 | 4.96438356 | 1 | 1.40295 | 0.06143187 | 0.1670179 | 0.05938414 | 0.1287849 | 0.7498575 | 0.2261455 | 0.2349263 | 2.031421 | 0.1238125 | 3.340691 | 0.1113453 | 0.2058904 |
| TCGA-BH-A0AV | 4.98630137 | 0 | 0.3863232 | 0.4235551 | 0.1040101 | 0.4754747 | 0.2577875 | 0.1962069 | 0.08047527 | 0 | 0.3549924 | 0.0367162 | 5.020614 | 0.04952862 | 0.2976487 |
| TCGA-E2-A1L7 | 5.03013699 | 0 | 0.4866543 | 0.3915609 | 0.4136562 | 0.04325816 | 0.5159708 | 0.0963937 | 0.2306288 | 0.3422624 | 1.215535 | 0.09019076 | 7.984727 | 0.02703635 | 0.4124454 |
| TCGA-AO-A12F | 5.04657534 | 0 | 0.5401148 | 0.1606832 | 0.04765719 | 0.1129652 | 0.7043311 | 0.4405176 | 0.365663 | 0.1675857 | 1.621638 | 0 | 5.337224 | 0.08825405 | 0.2937457 |
| TCGA-A2-A1FX | 5.06027397 | 0 | 0.8473175 | 0.1947856 | 0.1270976 | 0.0251057 | 0.1361153 | 0.2797197 | 0.1720927 | 0.2979578 | 0.9201638 | 0.01744798 | 8.146882 | 0.04707319 | 0.2611316 |
| TCGA-AC-A2FG | 5.07671233 | 0 | 0.7989208 | 0.4162959 | 0.01331535 | 0.1183586 | 0.4620265 | 0.4044056 | 0.3966433 | 0.4214088 | 0.7519255 | 0.1316112 | 5.392616 | 0.01479483 | 0.5539868 |
| TCGA-E2-A1IO | 5.08219178 | 0 | 0.466242 | 0.255588 | 0.08966193 | 0.09563939 | 0.1037054 | 0.2841555 | 0.8012665 | 0.6305894 | 0.5842225 | 0.06646753 | 4.784357 | 0.05977462 | 0.1381628 |
| TCGA-AO-A03M | 5.11232877 | 0 | 0.3192053 | 0.2629469 | 0.03990067 | 0.04728968 | 0.02563898 | 0.0878144 | 0.8644207 | 0.2806201 | 0.5488599 | 0.115029 | 2.577441 | 0.02955605 | 0.1229683 |
| TCGA-BH-A0BG | 5.1260274 | 0 | 0.5850886 | 0.9347967 | 0.02925443 | 0.05200788 | 0.3383645 | 0.2897275 | 0.09902769 | 0.1543091 | 0.6036208 | 0.07228894 | 5.356058 | 0 | 0.09015811 |
| TCGA-A2-A0D3 | 5.13150685 | 0 | 0.2992729 | 0.1146639 | 0.01662627 | 0.02955781 | 0.06410128 | 0.5049629 | 0.1575861 | 0.58466 | 1.047229 | 0.3286737 | 4.640414 | 0.0554209 | 0.384299 |
| TCGA-BH-A0BM | 5.13972603 | 0 | 0.2260707 | 0.1948885 | 0 | 0 | 0.4357989 | 0.07463131 | 0.4974193 | 0.6956021 | 1.779923 | 0.03491439 | 4.063177 | 0 | 0.1306347 |
| TCGA-A2-A0EW | 5.16164384 | 1 | 0.6445875 | 0.3869171 | 0.03581042 | 0.2546518 | 0.1725803 | 0.2837249 | 0.8485402 | 0.2518535 | 1.322231 | 0.06636679 | 4.419538 | 0.01989468 | 0.1379534 |
| TCGA-BH-A0AZ | 5.25753425 | 0 | 0.32990497 | 0.24648073 | 0 | 0 | 0 | 0.13613685 | 0.30710477 | 0.50754611 | 0.35826785 | 0.10190115 | 2.852698028 | 0 | 0.2541804 |
| TCGA-A2-A0EY | 5.2739726 | 0 | 0.9111126 | 0.1047256 | 0.1051284 | 0.1557457 | 0.1351047 | 0.30078 | 0.3558648 | 0.2464548 | 0.6849994 | 0.1515364 | 4.590728 | 0.09734109 | 0.2159941 |
| TCGA-BH-A1FJ | 5.27945205 | 1 | 0.2909458 | 0.04013046 | 0.2182094 | 0.07758556 | 0.5608594 | 0.09604814 | 0.2166705 | 0.5115531 | 0.9478778 | 0.07189395 | 4.599355 | 0 | 0.1569146 |
| TCGA-BH-A0H3 | 5.28219178 | 0 | 0.3174164 | 0.3429557 | 0.03174164 | 0.08464438 | 0.09178307 | 0.1676588 | 1.052981 | 0.5022854 | 0.8272935 | 0.03921747 | 5.319538 | 0.05290274 | 0.3179259 |
| TCGA-AR-A2LM | 5.30136986 | 0 | 0.8828995 | 0.2976826 | 0.04414497 | 0.05231997 | 0.1985637 | 0.1165865 | 0.5379585 | 0.6726853 | 1.086646 | 0.1272648 | 3.418168 | 0 | 0.2267479 |
| TCGA-E2-A150 | 5.30136986 | 0 | 0.3885591 | 0.1786479 | 0.1942796 | 0.1381544 | 0.07490296 | 0.2565452 | 0.02630584 | 0.3415904 | 0.6751435 | 0 | 5.726543 | 0.06475985 | 0.08981142 |
| TCGA-AR-A0TX | 5.40273973 | 0 | 0.2315706 | 0.2661731 | 0.04962228 | 0.08821739 | 0.1275432 | 0.3931559 | 0.4031373 | 0.1163306 | 0.8262895 | 0.1634917 | 2.764115 | 0.01837862 | 0.2039054 |
| TCGA-AO-A0JF | 5.42465753 | 0 | 0.1863326 | 0.2398764 | 0.05589977 | 0.1325031 | 0.1077586 | 0.07381537 | 0.2270682 | 0.06552354 | 0.6070563 | 0.06906535 | 3.271564 | 0.04140723 | 0.1435628 |
| TCGA-AR-A0U0 | 5.44657534 | 0 | 0.20565225 | 0.16073969 | 0.06169568 | 0.0731208 | 0.31715046 | 0.27156281 | 0.16707432 | 0.21695182 | 0.75933138 | 0.05081754 | 8.34190411 | 0.11425125 | 0.22182715 |
| TCGA-OL-A66J | 5.46849315 | 0 | 1.07828 | 0.5474028 | 0.02246417 | 0.2396178 | 0.6062619 | 0.2076468 | 0.7908402 | 0 | 1.366147 | 0.3330595 | 3.639128 | 0.04992038 | 0.3461573 |
| TCGA-BH-A18S | 5.50410959 | 1 | 0.3748007 | 0.2114863 | 0 | 0 | 0 | 0.02249648 | 0.4613523 | 0.1797246 | 0.5180298 | 0.06314648 | 4.621516 | 0 | 0.3937786 |
| TCGA-AR-A2LL | 5.51232877 | 0 | 0.7901275 | 0.08256296 | 0.01795744 | 0.06384869 | 0.5192514 | 0.2134145 | 0.2431473 | 0.4420294 | 1.092073 | 0.1774944 | 6.650082 | 0.03990543 | 0.1936983 |
| TCGA-GM-A3XN | 5.53150685 | 0 | 1.228532 | 0.3892831 | 0 | 0.4131999 | 0.2880309 | 0.8769029 | 0.7418116 | 0.4670391 | 1.658676 | 0.1846067 | 3.363125 | 0.01844642 | 0.1790754 |
| TCGA-AO-A03N | 5.56438356 | 0 | 0.8560379 | 0.08945015 | 0 | 0 | 0.187522 | 0.2312169 | 0.2897731 | 0 | 0.1690244 | 0.6730519 | 2.293854 | 0 | 0.02997944 |
| TCGA-V7-A7HQ | 5.56986301 | 0 | 1.215401 | 0.2211937 | 0 | 0 | 1.903641 | 0.3677968 | 0.4114194 | 0 | 0 | 0.1877068 | 3.171492 | 0.1688057 | 0.1170531 |
| TCGA-BH-A0DG | 5.59178082 | 0 | 0.9466993 | 0.18136 | 0.2366748 | 0.1051888 | 0.2281203 | 0.26044 | 0.267052 | 0.3467763 | 0.7710673 | 0.0731042 | 2.202506 | 0.1095717 | 0.1215665 |
| TCGA-B6-A408 | 5.67671233 | 0 | 0.4582668 | 0.1701787 | 0.0352513 | 0.06266897 | 0.3397715 | 0.5353154 | 0.1909238 | 0.3718818 | 1.492996 | 0.3484298 | 4.516671 | 0.09792027 | 0.2444392 |
| TCGA-AO-A03R | 5.72876712 | 0 | 0.2171419 | 0.1747118 | 0.1357137 | 0.04825375 | 1.203437 | 0.2508929 | 1.065802 | 0.1908939 | 0.7074305 | 0.1676771 | 5.641569 | 0.06031719 | 0.2091254 |
| TCGA-B6-A0X5 | 5.74520548 | 1 | 1.182052 | 0.5434723 | 0.07387826 | 0 | 0.1424159 | 0.4227422 | 0.06668842 | 0.08659723 | 0.6953248 | 0.09127816 | 6.668592 | 0.02736232 | 0.5312594 |
| TCGA-GM-A3XL | 5.77534247 | 0 | 1.145388 | 0.4062461 | 0.2945284 | 0 | 0.8201058 | 0.2592822 | 0.4431081 | 0.05753912 | 0.6041608 | 0.0404329 | 4.621745 | 0.1818076 | 0.05042755 |
| TCGA-E2-A14Y | 5.77808219 | 0 | 0 | 0.04370941 | 0.2614369 | 0.08450486 | 0.4581589 | 0.0627684 | 0.6114386 | 0 | 0.5162062 | 0.05872925 | 7.240923 | 0.07922331 | 0.1098699 |
| TCGA-AO-A03T | 5.81917808 | 0 | 0.8332023 | 0.2081965 | 0 | 0.06440211 | 0.174584 | 0.1195913 | 0.5395607 | 0.1273888 | 0.4327472 | 0.04475822 | 2.598591 | 0 | 0.02791103 |
| TCGA-BH-A1EN | 5.82739726 | 1 | 0.2657334 | 0.08145086 | 0.01476297 | 0.05249055 | 0.02845873 | 0.03898886 | 0.1599148 | 0.3633961 | 0.3206437 | 0.03647992 | 3.945833 | 0 | 0.06824615 |
| TCGA-E2-A1IG | 5.8630137 | 0 | 0.5747906 | 0.1409448 | 0.07663874 | 0.06812332 | 0.1477373 | 0.278303 | 0.129713 | 0.2694989 | 0.8738897 | 0.0473444 | 2.984434 | 0.04257708 | 0.118095 |
| TCGA-BH-A0DX | 5.90684932 | 0 | 0.51494372 | 0.25253945 | 0 | 0.24412147 | 0.03308875 | 0.18132819 | 0.27889758 | 0.12071941 | 1.23027277 | 0.19086717 | 2.33091514 | 0.11443194 | 0.15869855 |
| TCGA-B6-A40C | 5.92876712 | 0 | 0.6075828 | 0.2349066 | 0.2209392 | 0.07364639 | 0.292811 | 0.1823432 | 0.2056698 | 0.4370226 | 0.8697606 | 0.2047313 | 3.606945 | 0.030686 | 0.2127825 |
| TCGA-AO-A0JM | 5.98356164 | 0 | 0.1588616 | 0.07303982 | 0.0794308 | 0.04707011 | 0.1020797 | 0.06992533 | 0.2868023 | 0 | 0.1725194 | 0.03271281 | 4.657191 | 0 | 0.2039956 |
| TCGA-A2-A0ES | 6 | 0 | 0.8127582 | 0.5265518 | 0.05541533 | 0.3612259 | 0.4629072 | 0.3170946 | 0.8503792 | 0.4546899 | 1.36407 | 0.0912891 | 5.078771 | 0.0205242 | 0.5692747 |
| TCGA-AO-A0JD | 6 | 0 | 0.4729361 | 0.07526835 | 0.3274173 | 0.03233751 | 0.07012954 | 0.120098 | 0.1724059 | 0.3837858 | 0.2370442 | 0.06742184 | 5.748102 | 0.02021094 | 0.2522637 |
| TCGA-BH-A1FN | 6.00547945 | 1 | 0.8445514 | 0.08628877 | 1.219908 | 0.04170624 | 0.04522363 | 0.123914 | 0.3176498 | 0.2474876 | 0.8152532 | 0.2898503 | 6.806259 | 0.0521328 | 0.1084496 |
| TCGA-GM-A2DH | 6.00821918 | 0 | 0.4831413 | 0.3266675 | 0.1847305 | 0.05052458 | 0.1917499 | 0.5441643 | 0.8465896 | 0.2998162 | 1.08022 | 0.1053408 | 4.038687 | 0.03157786 | 0.8758675 |
| TCGA-BH-A0BO | 6.01917808 | 0 | 0.3892083 | 0.3877171 | 0.01621701 | 0.3747932 | 0.4689257 | 0.08565795 | 0.9442007 | 0.2851343 | 1.197564 | 0.2003646 | 3.298134 | 0.1261323 | 0.1499358 |
| TCGA-AC-A2BK | 6.08767123 | 0 | 0.7385317 | 0.3314702 | 0.1582568 | 0.0312606 | 0.2711763 | 0.1160985 | 0.2857104 | 0.2473366 | 0.5346836 | 0.3910592 | 4.921789 | 0.01953788 | 0.3522462 |
| TCGA-AR-A5QM | 6.11232877 | 0 | 0.85067871 | 0.27789867 | 0.02238628 | 0.1193935 | 0.73362273 | 0.20692684 | 0.66685229 | 0.55104694 | 0.97243577 | 0.22126981 | 2.42482661 | 0.09949459 | 0.27596572 |
| TCGA-E2-A1L8 | 6.1369863 | 0 | 0.9276378 | 0.1919251 | 0.01546063 | 0.1374278 | 0.238429 | 0.6328863 | 0.439614 | 0.3262023 | 2.014779 | 0.01910194 | 3.356724 | 0 | 0.405004 |
| TCGA-AC-A23G | 6.15890411 | 0 | 0.19610034 | 0.33059061 | 0.03268339 | 0.29051902 | 0.37802475 | 0.12947478 | 0.70806328 | 0.2298612 | 0.1419731 | 0.12114307 | 2.318350491 | 0.03631488 | 0.15108854 |
| TCGA-BH-A0BZ | 6.17808219 | 0 | 0.2368824 | 0.1293323 | 0.01480515 | 0.05264053 | 0.1141602 | 0.07820051 | 0.2205111 | 0.1561862 | 0.4501837 | 0.0731683 | 5.740525 | 0.03290033 | 0.09125485 |
| TCGA-AC-A2FO | 6.17808219 | 0 | 0.5096568 | 0.2793875 | 0 | 0.2787866 | 0.03778734 | 0.4918072 | 0.2388757 | 0.275723 | 1.192097 | 0.1937513 | 6.347859 | 0.0217802 | 0.09061684 |
| TCGA-A2-A0ER | 6.2 | 0 | 0.732368 | 0.2681296 | 0.06781185 | 0.02411088 | 0.07843299 | 0.1432724 | 0.1469097 | 0.2861511 | 0.2061971 | 0.08378298 | 4.04095 | 0.0150693 | 0.08359471 |
| TCGA-BH-A1FE | 6.22739726 | 1 | 0.82069435 | 0.44877995 | 0.2635508 | 0.40203297 | 0.23147365 | 0.11488705 | 0.2283718 | 0.315798 | 0.5420542 | 0.1188072 | 4.3502035 | 0.03081121 | 0.30106945 |
| TCGA-BH-A0BL | 6.24109589 | 0 | 1.124456 | 0.3537307 | 0.2071366 | 0.2893336 | 0.5989491 | 0.4102842 | 0.1001668 | 0.2601401 | 0.9319137 | 0.1096807 | 3.971065 | 0.09863645 | 0.1595916 |
| TCGA-AR-A0TV | 6.26849315 | 0 | 0.4302353 | 0.1071467 | 0.07170588 | 0.09560783 | 0.1036711 | 0.1183591 | 0.2670007 | 0.2521525 | 0.5061591 | 0.02214853 | 10.33309 | 0.0398366 | 0.02762345 |
| TCGA-A2-A0CT | 6.27123288 | 0 | 0.5171653 | 0.05944429 | 0 | 0 | 0 | 0.1138191 | 0.8169607 | 0.1515503 | 0.2808137 | 0.1064948 | 11.71518 | 0.04788568 | 0.06640967 |
| TCGA-BH-A0BP | 6.29041096 | 1 | 0.4426387 | 0.2148183 | 0.02459104 | 0.1311522 | 0 | 0.1948341 | 0.2996707 | 0.172948 | 0.9613872 | 0.0607655 | 3.767597 | 0 | 0.2273585 |
| TCGA-E2-A1LB | 6.31780822 | 0 | 0.565051 | 0.07422674 | 0.1614432 | 0.03587626 | 0.03890196 | 0.1332406 | 0.0546493 | 0.2128921 | 0.2629843 | 0.07479992 | 2.311627 | 0.02242266 | 0.4042557 |
| TCGA-E2-A14T | 6.33150685 | 0 | 1.354153 | 0.05764807 | 0.06269227 | 0.02229059 | 0.07251155 | 0.2317976 | 0.6790926 | 0.1763651 | 0.4629583 | 0.09294916 | 5.36443 | 0 | 0.1159254 |
| TCGA-E2-A1AZ | 6.38082192 | 0 | 0.53642203 | 0.56372758 | 0.91958061 | 0.06811708 | 0.14772379 | 0.50595907 | 0.36316301 | 0.26947417 | 0.41609983 | 0.3313804 | 6.50700079 | 0.08514635 | 0.05904209 |
| TCGA-AO-A0JE | 6.39726027 | 0 | 0.5442183 | 0.1417887 | 0.07256244 | 0.09674992 | 0.3147286 | 0.2155913 | 0.1719392 | 0.2551646 | 0.2364025 | 0.1568918 | 3.283604 | 0.02015623 | 0.3074877 |
| TCGA-A2-A0CS | 6.43287671 | 1 | 0.713433 | 0.05125237 | 0 | 0 | 0.08595578 | 0.1177606 | 0.09056272 | 0 | 0.4358075 | 0 | 1.845024 | 0 | 0.03435472 |
| TCGA-B6-A0I1 | 6.46849315 | 1 | 1.189295 | 0.04784519 | 0.05946474 | 0 | 0.487181 | 0.4515067 | 0.181162 | 0.0522767 | 0.355174 | 0.3122473 | 6.042069 | 0.06607193 | 0 |
| TCGA-E2-A1IE | 6.47123288 | 0 | 0.6361122 | 0.1286848 | 0.1399447 | 0.02261732 | 0.2207233 | 0.1847963 | 0.3272972 | 0.4473756 | 0.9394889 | 0.07859302 | 4.067053 | 0.01413583 | 0.0588123 |
| TCGA-BH-A0BT | 6.47945205 | 0 | 1.62575553 | 0.13590433 | 0.0184745 | 0.13137419 | 0.17806742 | 0.53670059 | 0.22513348 | 0.06496526 | 0.80251199 | 0.13695378 | 2.747838972 | 0 | 0.17080753 |
| TCGA-BH-A0BW | 6.49589041 | 0 | 1.04770984 | 0.44729813 | 0.26192746 | 0.06652126 | 0.32459169 | 0.19764226 | 0.43065244 | 1.18422462 | 1.74731182 | 0.04623099 | 5.221736217 | 0.04157579 | 0.11531775 |
| TCGA-AO-A12C | 6.49863014 | 0 | 0.9714361 | 0.1202485 | 0.05604439 | 0.03321149 | 0.2160748 | 0.1480127 | 0.2023606 | 0.1313861 | 1.298404 | 0.04616269 | 3.554608 | 0.08302873 | 0.05757369 |
| TCGA-BH-A0DE | 6.49863014 | 0 | 0.64883497 | 0.29831493 | 0.01908338 | 0.23748208 | 0.18393621 | 0.40319222 | 0.87853536 | 0.20131919 | 0.53882489 | 0.07073377 | 5.265222149 | 0.02120376 | 0.14703087 |
| TCGA-B6-A0RM | 6.50136986 | 1 | 0.775087 | 0.1862801 | 0.2113874 | 0.1252666 | 0.2377047 | 0.04652267 | 0.214667 | 0.06194502 | 1.224325 | 0.02176447 | 9.1464 | 0 | 0.08143334 |
| TCGA-A2-A04Q | 6.53424658 | 0 | 0.8632609 | 0.454783 | 0.05395381 | 0 | 0.5200367 | 0.3799773 | 0.2191636 | 0.06324256 | 1.367155 | 0.1777629 | 4.953443 | 0.1598631 | 0.1662782 |
| TCGA-BH-A0DT | 6.58356164 | 0 | 0.62991504 | 0.2624646 | 0.01968485 | 0.03499528 | 0.11384007 | 0.1819562 | 0.10661468 | 0.48455003 | 1.11161478 | 0.14592627 | 3.805278087 | 0.02187205 | 0.18199793 |
| TCGA-GM-A2DB | 6.59178082 | 0 | 0.3693418 | 0.05660411 | 0.09233546 | 0.1641519 | 0.8899804 | 1.056714 | 0.5000962 | 0 | 0.4010952 | 0.6844945 | 1.843943 | 0 | 0.04742752 |
| TCGA-B6-A0WV | 6.62191781 | 1 | 1.833697 | 0.05546574 | 0.1930208 | 0.0428935 | 0.09302206 | 0.1911622 | 0.5880463 | 0.08484429 | 1.467307 | 0.08943047 | 4.432828 | 0 | 0.03717896 |
| TCGA-A2-A0EQ | 6.64657534 | 0 | 0.7718562 | 0.3460045 | 0.1350748 | 0.03430472 | 0.4091768 | 0.6115394 | 0.3135325 | 0 | 1.299233 | 0.02384112 | 7.343231 | 0.06432135 | 0.3270787 |
| TCGA-AO-A03L | 6.69041096 | 0 | 0.3313675 | 0.1752058 | 0.0662735 | 0 | 0.1277562 | 0.1312708 | 0.08973563 | 0.1165248 | 0.4318274 | 0.1432941 | 2.490798 | 0.05522792 | 0.1021229 |
| TCGA-B6-A0RL | 6.76438356 | 1 | 0.2971399 | 0.03035912 | 0.1980933 | 0 | 1.018311 | 0.2179843 | 0.3352778 | 0.1160986 | 0.6453718 | 0.08158282 | 4.135267 | 0.07336788 | 0.6359335 |
| TCGA-BH-A0B6 | 6.80273973 | 0 | 0.2234853 | 0.2397544 | 0.0744951 | 0 | 0.1077038 | 0.09837043 | 0.1260848 | 0 | 0.04044983 | 0.02301007 | 3.725127 | 0 | 0.08609386 |
| TCGA-AO-A03O | 6.80273973 | 1 | 0.09040615 | 0.1039151 | 0 | 0.1205415 | 0 | 0.02984522 | 0.1836176 | 0.2384338 | 0.5890718 | 0.02792468 | 10.82527 | 0 | 0 |
| TCGA-BH-A0E9 | 6.81917808 | 0 | 0.8392509 | 0.5368522 | 0.05473376 | 0.2270437 | 0.1055109 | 0.7227566 | 1.408101 | 0.4490975 | 1.386919 | 0.2028742 | 3.835826 | 0.02027176 | 0.2249092 |
| TCGA-AO-A1KR | 6.88493151 | 0 | 1.635047 | 0.08487452 | 0.02637173 | 0 | 0.7117189 | 0.1741187 | 0.5356176 | 0.5564144 | 0.9164473 | 0.5864909 | 2.329875 | 0.05860384 | 0.121911 |
| TCGA-E2-A106 | 6.96164384 | 0 | 0.3842821 | 0.05621682 | 0.01746737 | 0.0931593 | 0.06734408 | 0.1845246 | 0.2601628 | 0.2456949 | 0.9105163 | 0.1294878 | 6.11698 | 0.01940819 | 0.2153281 |
| TCGA-AR-A0TS | 7.00821918 | 0 | 0.813559 | 0.8182346 | 0.4915252 | 0.2410545 | 0.03267305 | 0.3804815 | 0.1606464 | 0.1788042 | 1.362067 | 0.3350565 | 6.755965 | 0.2824858 | 0.1567048 |
| TCGA-BH-A0B7 | 7.0109589 | 0 | 0.2415015 | 0.3251745 | 0.03450022 | 0.03066686 | 0.09975966 | 0.2050082 | 0.1634989 | 0.5459375 | 0.4121293 | 0.170503 | 3.169266 | 0 | 0.239231 |
| TCGA-B6-A0IN | 7.04931507 | 1 | 0.3717839 | 0.2564027 | 0 | 0.06008629 | 0.2931922 | 0.2901003 | 0.02288195 | 0 | 0.1835215 | 0.1670353 | 2.659336 | 0.0563309 | 0.05208122 |
| TCGA-GM-A2DI | 7.09589041 | 0 | 0.08217258 | 0.604488 | 0.04108629 | 0.1460846 | 0 | 0.2712713 | 0.7788431 | 0.5779171 | 0.8031347 | 0.152289 | 4.154987 | 0.1369543 | 0.3165557 |
| TCGA-B6-A401 | 7.11232877 | 0 | 1.90903878 | 0.18159703 | 0 | 0.35108759 | 1.2689913 | 0.56502405 | 0.53480197 | 0.86807372 | 1.67997796 | 0.10166629 | 3.034111944 | 0.07314325 | 0.1775162 |
| TCGA-AR-A254 | 7.1369863 | 0 | 0.5182448 | 0.1667914 | 0.2979907 | 0.0460662 | 0.2247809 | 0.1197594 | 0.1403427 | 0.1822399 | 0.9286196 | 0.0800378 | 9.150914 | 0.08637413 | 0.07985794 |
| TCGA-AR-A1AX | 7.20273973 | 0 | 0.99772046 | 0.32280441 | 0.09238152 | 0.06569353 | 0.5342546 | 0.36596807 | 0.12508641 | 0.38982973 | 1.6854403 | 0.11413934 | 6.827469692 | 0.06158768 | 0.11388284 |
| TCGA-AR-A2LJ | 7.2109589 | 0 | 0.1434542 | 0.2061124 | 0 | 0.03187871 | 0.5185092 | 0.2604671 | 0.4855996 | 0 | 0.2336811 | 0.04431018 | 2.415015 | 0.0199242 | 0.1105265 |
| TCGA-AR-A1AW | 7.2109589 | 0 | 3.407062 | 0.7194812 | 0.118851 | 0.1408605 | 0.4582207 | 0.8108679 | 0.4023165 | 0.8358752 | 1.591851 | 0.1468429 | 8.01913 | 0.1320567 | 0.5189001 |
| TCGA-AC-A2FE | 7.22191781 | 1 | 0.8998475 | 0.2881285 | 0 | 0.08569976 | 0.7124438 | 0.4031543 | 0.3481175 | 0.2260214 | 0.6631068 | 0.1389726 | 4.46426 | 0 | 0.1238038 |
| TCGA-AC-A2FK | 7.26027397 | 0 | 0.4957267 | 0.9013212 | 0.04506606 | 0.08011744 | 0.3474973 | 0.4165666 | 0.5796932 | 0.8716073 | 1.027751 | 0.1670402 | 5.266632 | 0.1251835 | 0.2430529 |
| TCGA-E2-A1B1 | 7.26849315 | 0 | 0.8401611 | 0.3189064 | 0.1074625 | 0.1215737 | 0.2636538 | 0.2838073 | 0.7936708 | 0.6183655 | 0.8275185 | 0.1448424 | 4.861734 | 0.03256438 | 0.286023 |
| TCGA-A2-A04U | 7.27123288 | 0 | 2.572988 | 0.2566849 | 0.1213673 | 0.1726113 | 0 | 0.416689 | 0.3615343 | 0 | 0.3690446 | 0.5998077 | 2.892942 | 0 | 0.3740374 |
| TCGA-AR-A250 | 7.41643836 | 0 | 1.131986 | 0.1537703 | 0.2958599 | 0.1372104 | 0.1239853 | 0.1868477 | 0.8360352 | 0.6332788 | 1.229306 | 0.07946548 | 4.052605 | 0.04287825 | 0.2576825 |
| TCGA-BH-A0C3 | 7.42191781 | 0 | 0.5714593 | 0.3753427 | 0.2245019 | 0.1814156 | 0.1573726 | 0.2156026 | 0.359249 | 0.2870753 | 0.8865561 | 0.07564822 | 3.329063 | 0.06803086 | 0.125797 |
| TCGA-BH-A1F5 | 7.43013699 | 1 | 0.3693499 | 0.2392863 | 0.03357726 | 0 | 0.1941818 | 0.1330158 | 0.5683035 | 0.3542217 | 0.838672 | 0.08297085 | 13.10771 | 0.01865404 | 0.07761037 |
| TCGA-BH-A18K | 7.56986301 | 1 | 0.7158836 | 0.1332241 | 0.1363588 | 0.03030195 | 0.1642877 | 0.1575535 | 0.2769487 | 0.3596276 | 0.7404097 | 0.1684742 | 2.793891 | 0.01893872 | 0.1313247 |
| TCGA-BH-A0C7 | 7.58082192 | 0 | 0.6625585 | 0.1949598 | 0.1722652 | 0.04711527 | 0.2298998 | 0.5074451 | 0.9868291 | 0.2795851 | 0.8346439 | 0.04911631 | 4.562161 | 0.07361762 | 0.04083828 |
| TCGA-A2-A3XT | 7.5890411 | 0 | 0.8635583 | 1.861973 | 0.4317791 | 0.05293844 | 0.8323453 | 0.2949115 | 0.3628784 | 0.523567 | 0.840787 | 0.183956 | 5.137909 | 0.2150624 | 0.1605998 |
| TCGA-BH-A1EO | 7.66575342 | 1 | 0.2762168 | 0.1650951 | 0.05524336 | 0 | 0.1064932 | 0.0364743 | 0.2992024 | 0 | 0.05999279 | 0 | 12.71783 | 0.06138151 | 0.1276893 |
| TCGA-A2-A0CP | 7.70684932 | 0 | 0.743817 | 0.302525 | 0 | 0.1525778 | 0.4411889 | 0.9444295 | 0.4260989 | 0.3018023 | 1.739802 | 0.3534622 | 3.628961 | 0.03178705 | 0.3967514 |
| TCGA-AR-A252 | 7.77534247 | 0 | 0.4731472 | 0.367097 | 0.0591434 | 0.07885786 | 0.08550853 | 0.3904929 | 0.300305 | 0.5719362 | 0.5780531 | 0.2009505 | 4.88029 | 0.06571489 | 0.2050558 |
| TCGA-AR-A256 | 7.81917808 | 1 | 0.6016753 | 0.1521478 | 0.1353769 | 0.1069645 | 0.0579928 | 0.1589019 | 0.5295421 | 0.2644726 | 0.8167538 | 0.03716913 | 4.969603 | 0.1504188 | 0.2086065 |
| TCGA-AR-A1AP | 7.82465753 | 0 | 0.4443705 | 0.08172331 | 0.04443705 | 0.1579984 | 0.2569853 | 0.2053762 | 0.5415178 | 0 | 1.158178 | 0.1098058 | 4.799113 | 0.02468725 | 0.1027116 |
| TCGA-AR-A24L | 7.85205479 | 1 | 0.9420717 | 0.2165682 | 0.1611439 | 0.0661103 | 0.1911623 | 0.03273687 | 0.3021119 | 0.4358921 | 0.5384549 | 0.04594538 | 4.632012 | 0.02754596 | 0.343816 |
| TCGA-AR-A1AU | 7.85753425 | 0 | 0.92782732 | 0.28712605 | 0.07137133 | 0.25376474 | 0 | 0.35342063 | 1.18381787 | 0.56469626 | 0.73631963 | 0.0661356 | 7.214811479 | 0.03965074 | 0.27494574 |
| TCGA-AO-A03P | 7.97534247 | 1 | 2.811441 | 0.04712664 | 0.04392876 | 0.02603186 | 0.9032742 | 0.05800772 | 0.1189608 | 0 | 0.254429 | 0.01809164 | 4.33717 | 0 | 0.06769119 |
| TCGA-AR-A1AN | 8 | 0 | 0.224333 | 0.2210177 | 0 | 0.02848672 | 0.06177844 | 0.08463731 | 0.06508955 | 0.05634737 | 0.1392111 | 0.09898862 | 3.809228 | 0 | 0.1975323 |
| TCGA-B6-A0WS | 8.12328767 | 1 | 0.2792556 | 0.2648114 | 0.1221743 | 0 | 0.3028073 | 0.0691417 | 0.1654265 | 0.1227497 | 0.4928041 | 0.1078207 | 4.776484 | 0.01939275 | 0.1075784 |
| TCGA-B6-A0IH | 8.12328767 | 1 | 1.185772 | 0.3318501 | 0.02577764 | 0.2291346 | 0.04969184 | 0.7148243 | 0.8725859 | 0.27194 | 0.6158641 | 0.2866394 | 4.67009 | 0.1432091 | 0.1986078 |
| TCGA-AR-A1AL | 8.13972603 | 0 | 0.7907947 | 0.3003518 | 0.08595594 | 0.275059 | 0.364536 | 0.2043079 | 1.722513 | 0.7254304 | 1.456195 | 0.1062004 | 5.709912 | 0.07640528 | 0.60928 |
| TCGA-AR-A24S | 8.15342466 | 0 | 1.37836 | 0.2780646 | 0.267233 | 0.1750298 | 0.4066957 | 0.5386061 | 0.4761036 | 0.8408024 | 1.160831 | 0.3475495 | 5.929866 | 0.01562766 | 0.1300382 |
| TCGA-AO-A12B | 8.1890411 | 0 | 0.2482401 | 0.06657781 | 0 | 0.07355263 | 0.1196338 | 0.05463332 | 0.2520915 | 0.07274436 | 0.3145124 | 0.1022353 | 3.807258 | 0.0229852 | 0.03187674 |
| TCGA-AR-A1AM | 8.19452055 | 0 | 0.93277816 | 0.31735901 | 0.01865556 | 0.06633089 | 0.35962531 | 0.54196079 | 0.37890001 | 0.26240792 | 1.01297175 | 0.36878951 | 4.345451586 | 0.04145681 | 0.25872242 |
| TCGA-AR-A24X | 8.23013699 | 0 | 0.5142778 | 0.09457982 | 0.07714167 | 0 | 0.24784472 | 0.1018652 | 0.13926846 | 0.27126741 | 0.22339669 | 0.09531017 | 4.453137338 | 0.02857099 | 0.31698664 |
| TCGA-AR-A0TW | 8.24383562 | 0 | 0.199327 | 0.1145557 | 0 | 0 | 0.160102 | 0.02193419 | 0.1349463 | 0.1752325 | 0.2886182 | 0.04104545 | 3.741734 | 0.092281 | 0.07678727 |
| TCGA-A2-A0CV | 8.24931507 | 0 | 0.70175634 | 0.17422916 | 0.01403513 | 0.07485401 | 0.59522466 | 0.12973322 | 0.19003839 | 0.09870859 | 0.33531887 | 0.10404418 | 4.392581292 | 0.04678376 | 0.41091607 |
| TCGA-A2-A0CL | 8.26027397 | 0 | 1.246938 | 0.6449677 | 0.1363838 | 0.2770972 | 0.3004669 | 0.385916 | 0.4748563 | 0.5481044 | 1.90426 | 0.09628861 | 3.999787 | 0.1082411 | 0.1501129 |
| TCGA-A2-A0ST | 8.26575342 | 0 | 0.54378357 | 0.41348707 | 0 | 0.2230907 | 0.64508153 | 0.69044576 | 0.31150887 | 0.51482468 | 0.99936556 | 0.23256559 | 3.104112686 | 0.1626703 | 0.12891276 |
| TCGA-AC-A2BM | 8.27945205 | 0 | 1.81784 | 0.1305919 | 0.1514866 | 0.1009911 | 0.1095084 | 0.2500467 | 0.2307554 | 0.4661127 | 0.3701483 | 0.04679124 | 9.105837 | 0.02103981 | 0.08753642 |
| TCGA-AR-A251 | 8.30136986 | 0 | 0.5317173 | 1.471334 | 0.3938647 | 0.1050306 | 0.2277772 | 0.2600482 | 0.6266282 | 0.5540075 | 0.3421811 | 0.121657 | 11.99016 | 0.3610426 | 0.09103774 |
| TCGA-AR-A24N | 8.31506849 | 0 | 0.3365463 | 0.05802523 | 0.1963187 | 0.02492936 | 0.4054775 | 0.09258496 | 0.189871 | 0.3451757 | 1.096441 | 0.06930169 | 5.793369 | 0.06232339 | 0.1296487 |
| TCGA-B6-A0RS | 8.39178082 | 1 | 0.4375137 | 0.1005779 | 0.1020865 | 0.05185347 | 0.05622666 | 0.327383 | 0.2567076 | 0.05128365 | 0.5384784 | 0 | 3.981819 | 0.03240842 | 0.1573083 |
| TCGA-A2-A04W | 8.49863014 | 0 | 0.3581848 | 0.123512 | 0.02238655 | 0 | 0.1726192 | 0.02956133 | 0.181871 | 0.3148877 | 0.3403566 | 0.08297717 | 1.591535 | 0 | 0.1724806 |
| TCGA-AO-A12A | 8.5260274 | 0 | 0.5280357 | 0.38844 | 0.05280357 | 0.156455 | 0.30537 | 0.1859383 | 0.4766472 | 0.9903087 | 1.376238 | 0.1087332 | 3.405298 | 0.03911375 | 0.2441 |
| TCGA-E2-A1LI | 8.55068493 | 0 | 1.36653 | 1.051423 | 0.1673302 | 0.1239483 | 0.3225642 | 0.4419174 | 0.2643298 | 0.04903449 | 0.9388662 | 0.08614167 | 10.65745 | 0.1549354 | 0.3867664 |
| TCGA-B6-A0RP | 8.56438356 | 1 | 0.7843247 | 0.1602707 | 0.01452453 | 0.05164278 | 0.1679946 | 0.1917957 | 0.5309956 | 0.3575269 | 1.009488 | 0.1256176 | 4.408844 | 0.01613837 | 0.3581008 |
| TCGA-AR-A24U | 8.56986301 | 0 | 0.6107017 | 0.1995033 | 0.160711 | 0.171425 | 0.1549021 | 0.106109 | 0.3916905 | 0.3390825 | 0.8028277 | 0.1787056 | 9.340653 | 0.1071407 | 0.09905781 |
| TCGA-B6-A40B | 8.63561644 | 0 | 0.12137539 | 0.09068276 | 0.01517192 | 0.02697231 | 0.96515371 | 0.40068905 | 1.91050686 | 0.05335182 | 0.98857785 | 0.18745234 | 2.317221643 | 0 | 0.25716776 |
| TCGA-AR-A24Q | 8.69041096 | 0 | 1.247834 | 0.3824778 | 0.02772964 | 0 | 0.294001 | 0.2013927 | 0.1877324 | 0.09751083 | 0.8431819 | 0.05139084 | 7.528951 | 0.04621607 | 0.4700241 |
| TCGA-AR-A24T | 8.77260274 | 0 | 0.5379904 | 0.2182517 | 0.04746974 | 0.1406511 | 0.2135185 | 0.2716288 | 0.3427998 | 0.5564219 | 0.6873446 | 0.03909991 | 4.016877 | 0.01758139 | 0.2925904 |
| TCGA-AR-A24V | 8.77534247 | 0 | 1.030447 | 0.1856662 | 0.01392496 | 0.04951099 | 0.05368661 | 0.09193925 | 0.1696921 | 0.09793382 | 0.6048854 | 0.03440918 | 6.028822 | 0.01547218 | 0.1287445 |
| TCGA-E2-A1LH | 8.89589041 | 0 | 0.7034802 | 0.4998606 | 0.09592912 | 0.1136938 | 0.3698472 | 0.844493 | 0.3030765 | 0 | 1.389019 | 0.3555674 | 6.166028 | 0.07105861 | 0.09854677 |
| TCGA-AO-A128 | 8.89863014 | 0 | 0.5241786 | 0.3361339 | 0.04138252 | 0.0980919 | 0.5850059 | 0.7286056 | 0.1307431 | 0.2425349 | 0.8688456 | 0.1533869 | 5.150192 | 0.06130744 | 0.06376761 |
| TCGA-AR-A0U4 | 8.93424658 | 0 | 0.35092777 | 0.75025937 | 0.1579175 | 0.1559679 | 0.16912182 | 0.13901953 | 0.35637235 | 0.06170159 | 0.64786665 | 0.15175255 | 6.534299567 | 0.07798395 | 0.37852883 |
| TCGA-A2-A25A | 8.97534247 | 0 | 0.7606704 | 0.6428925 | 0.02237266 | 0.1193209 | 0.3018961 | 0.3692874 | 1.151135 | 0.5900482 | 1.384878 | 0.2211352 | 4.659053 | 0.03728777 | 0.5343582 |
| TCGA-A2-A0CW | 8.99452055 | 0 | 0.2678315 | 0.04104697 | 0.05951811 | 0 | 0.1147337 | 0.1178901 | 2.034864 | 0.3139417 | 0.5493979 | 0.1103038 | 2.907265 | 0.04959842 | 0.02292832 |
| TCGA-A2-A0CR | 8.99452055 | 0 | 0.821656 | 0.8095133 | 0 | 0 | 0.9805189 | 0.7749949 | 0.7681813 | 0.06879381 | 0.08498058 | 0.4834159 | 2.144045 | 0.0652108 | 0.1808736 |
| TCGA-AO-A129 | 9.00273973 | 0 | 0.92196533 | 0.483237 | 0.03687861 | 0.32780989 | 0.28436521 | 0.48698031 | 0.12483593 | 0.12968304 | 0.80098345 | 0.11391077 | 2.745551064 | 0 | 0.14206849 |
| TCGA-AR-A0TT | 9.08493151 | 0 | 0.6951567 | 0.2996365 | 0 | 0.03861982 | 0.3350153 | 0.3442316 | 0.3235567 | 0.5347359 | 1.46266 | 0.1610402 | 6.462215 | 0.04827477 | 0.3347464 |
| TCGA-GI-A2C9 | 9.15616438 | 0 | 0.80229466 | 0.7672519 | 0.06418357 | 0.02852603 | 0.12372737 | 0.23307377 | 0.26071747 | 0.33855071 | 1.28947992 | 0.01982504 | 5.244916212 | 0.01782877 | 0.24725614 |
| TCGA-GM-A3NW | 9.20821918 | 0 | 0.4802112 | 0.5519669 | 0.01846966 | 0.2955146 | 0.2848334 | 0.3170583 | 1.625539 | 0.3896896 | 1.083108 | 0.2281966 | 4.331657 | 0.04104369 | 0.1423024 |
| TCGA-A8-A08T | 9.33972603 | 1 | 0.8590201 | 0.1594997 | 0.08259808 | 0.146841 | 0.09553513 | 0.5017237 | 0.3355183 | 0.4647277 | 1.076391 | 0.02041034 | 9.844141 | 0.01835513 | 0.2545559 |
| TCGA-AR-A24R | 9.39726027 | 0 | 0.5386132 | 0.07168476 | 0.07087016 | 0 | 0.1912641 | 0.1684507 | 0.710101 | 0.199371 | 1.077483 | 0.07004927 | 6.859015 | 0.1102425 | 0.1747296 |
| TCGA-B6-A0WY | 9.48219178 | 1 | 0.365082 | 0.3204482 | 0.01659464 | 0.1180063 | 0.06397932 | 0.1753051 | 0.1123473 | 0.1750643 | 0.3243839 | 0.06150908 | 4.854283 | 0 | 0.2301407 |
| TCGA-A2-A0EP | 9.87123288 | 0 | 0.4286416 | 0.703845 | 0 | 0.2721534 | 0.7672759 | 0.6468791 | 0.22801 | 0.3768278 | 2.127969 | 0.3404544 | 3.07426 | 0.05102876 | 0.1651268 |
| TCGA-BH-A1FB | 10.0520548 | 1 | 0.2903716 | 0.3137348 | 0.01451858 | 0.05162162 | 0.2518886 | 0.134202 | 0.6683871 | 0.4594891 | 0.6306714 | 0.1255661 | 6.167533 | 0.09679053 | 0.2460934 |
| TCGA-A2-A04R | 10.1616438 | 0 | 0.5532527 | 0.03179613 | 0.2420481 | 0.03073626 | 0.2666278 | 0.6164164 | 0.4916068 | 0.303985 | 0.7885729 | 0.04272222 | 3.886303 | 0.03842033 | 0.1332069 |
| TCGA-AR-A1AH | 10.430137 | 0 | 1.694025 | 0.1668991 | 0.622295 | 0.3380368 | 0.7330919 | 0.2054344 | 0.3042721 | 0.1823575 | 1.914754 | 0.3203578 | 6.456652 | 0 | 0.4261839 |
| TCGA-B6-A0IP | 10.7561644 | 1 | 0.4534469 | 0.1433309 | 0.1275319 | 0.02519149 | 0.3004768 | 0.3368106 | 0.2494278 | 0.4484639 | 1.723508 | 0.175076 | 3.203332 | 0.03148937 | 0.2401886 |
| TCGA-B6-A0IB | 10.7972603 | 1 | 1.08205 | 0.09799132 | 0.0327894 | 0.02914614 | 0.4108552 | 0.4979299 | 0.1997887 | 0 | 0.8546016 | 0 | 3.50714 | 0.03643267 | 0.1263155 |
| TCGA-AQ-A04L | 10.8410959 | 0 | 0.6565419 | 0.04766184 | 0 | 0.03071541 | 0 | 0.182518 | 0.3275155 | 0 | 0.8630891 | 0.06403985 | 5.721991 | 0.01919713 | 0.3727263 |
| TCGA-3C-AAAU | 11.0876712 | 0 | 0.9344455 | 0.04957271 | 0.2695516 | 0.1597343 | 1.1778 | 0.2847533 | 0.1703232 | 0.1263832 | 0.6635116 | 0.1776196 | 4.630779 | 0.01996679 | 0.4153604 |
| TCGA-AR-A0U3 | 11.1780822 | 0 | 0.5172273 | 0.3170742 | 0.1436743 | 0.05108418 | 0.05539249 | 0.4173866 | 0.350168 | 0.3031369 | 0.3120527 | 0 | 3.539422 | 0.03192761 | 0.3542274 |
| TCGA-A2-A0CK | 11.3945205 | 0 | 0.3920801 | 0.3425067 | 0.019604 | 0.06970312 | 0.1889543 | 0.2070959 | 1.300666 | 0.06893716 | 0.8089978 | 0.145327 | 4.068394 | 0.04356445 | 0.2114589 |
| TCGA-B6-A1KN | 11.5972603 | 0 | 1.339767 | 0.1796622 | 0.05582362 | 0.1240525 | 0.5918649 | 0.2027158 | 0.9826216 | 0.7852113 | 0.9699669 | 0.1551853 | 7.97751 | 0.09303936 | 0.08602034 |
| TCGA-B6-A0RQ | 11.690411 | 1 | 1.28564319 | 0.52622345 | 0 | 0.39022232 | 0.4533564 | 0.39336619 | 0.23352011 | 0.16540036 | 1.25996154 | 0.09685607 | 3.894987103 | 0.06968256 | 0.14495762 |
| TCGA-B6-A0IQ | 11.739726 | 0 | 1.818088 | 0.2026431 | 0.2616945 | 0.07345811 | 0.1327556 | 0.4365049 | 0.1491956 | 0.4359053 | 3.739384 | 0.3743811 | 1.958394 | 0.09182264 | 0.4669248 |
| TCGA-A2-A04N | 11.9287671 | 0 | 0.4857048 | 0.08588944 | 0.01868095 | 0.06642117 | 0.1800574 | 0.4193578 | 0.3035326 | 0.2627651 | 1.257794 | 0.06924214 | 7.864094 | 0.04151323 | 0.1727164 |
| TCGA-B6-A0I2 | 11.9479452 | 0 | 0.9747811 | 1.38038 | 0.05848687 | 0.1732944 | 0.6013108 | 0.4376459 | 0.5015517 | 0 | 0.6351515 | 0.1204363 | 3.715751 | 0.108309 | 0.2403313 |
| TCGA-B6-A0IG | 12.2082192 | 1 | 0.59671255 | 0.03919294 | 0.08524465 | 0.02525767 | 0.41081759 | 0.39397803 | 0.15389725 | 0.19984094 | 0.70972922 | 0.12287517 | 2.371926364 | 0.01578605 | 0.08757075 |
| TCGA-AR-A24H | 13.4082192 | 0 | 1.117624 | 0.2169589 | 0.3849593 | 0.1545356 | 0.1675688 | 0.09838779 | 0.1681427 | 0.1746714 | 1.564336 | 0.1841131 | 5.882296 | 0.02759565 | 0.4401129 |
| TCGA-AR-A2LE | 13.8684932 | 0 | 0.3694917 | 0.09437847 | 0.04105464 | 0.2189581 | 0.1187122 | 0.487912 | 2.112374 | 0.5774718 | 1.070021 | 0.1014477 | 4.122271 | 0.06842439 | 0.06326236 |
| TCGA-B6-A0RV | 14.1260274 | 0 | 0.6771314 | 0.2821381 | 0.04232072 | 0.07523683 | 0.0815821 | 0.1955951 | 0.2005608 | 0.2232301 | 0.5055506 | 0.1045763 | 5.30414 | 0.02351151 | 0.3586732 |
| TCGA-B6-A2IU | 14.1808219 | 0 | 0.30375019 | 0.15711217 | 0.34171896 | 0 | 0.21957845 | 0.45123825 | 0.25705235 | 0.93461597 | 0.65972892 | 0.09382245 | 5.52603942 | 0 | 0.11701452 |
| TCGA-B6-A0WT | 15.7232877 | 0 | 0.712512 | 0.1191243 | 0.06477382 | 0 | 0.06243259 | 0.4704343 | 0.1315575 | 0.4555521 | 0.4923982 | 0.1200441 | 4.465286 | 0 | 0.149718 |
| TCGA-B6-A0WZ | 17.2383562 | 0 | 0.3243687 | 0.04971168 | 0.07208194 | 0.06407283 | 0.4515977 | 0.3331435 | 0.3904015 | 0.5069499 | 1.682999 | 0.2003822 | 1.969208 | 0 | 0.5831348 |
| TCGA-B6-A0RH | 17.6876712 | 1 | 0.2299416 | 0.1651879 | 0.0574854 | 0.1277453 | 0.1939267 | 0.2467049 | 0.05837728 | 0 | 0.1248552 | 0.159805 | 2.408475 | 0 | 0.08858103 |
| TCGA-GM-A2DA | 18.0630137 | 1 | 0.354597 | 0.1834122 | 0.04432462 | 0.3151973 | 0.3417802 | 0.7608959 | 1.080295 | 0.779334 | 2.021684 | 0.5476401 | 2.450136 | 0.09849916 | 0.3415059 |
| TCGA-B6-A0IJ | 19.4684932 | 0 | 0.509004 | 0.9176237 | 0.01339484 | 0.1428783 | 0.8521057 | 0.1945661 | 0.2176426 | 0 | 0.5236717 | 0.09929768 | 2.998091 | 0.1488316 | 0.1651242 |
| TCGA-B6-A0RI | 19.5232877 | 0 | 0.24965543 | 0.09326208 | 0.2184485 | 0 | 0.12031587 | 0.0824172 | 0.19014659 | 0.16460797 | 0.98280643 | 0.13494888 | 2.99636367 | 0.06934873 | 0.14426317 |
| TCGA-B6-A0X1 | 20.4246575 | 1 | 0.57057577 | 0.31480043 | 0.45646062 | 0.05071785 | 0.27497628 | 0.33904915 | 0.19314272 | 0 | 0.55766682 | 0.03524792 | 2.374597923 | 0.06339731 | 0 |
| TCGA-B6-A0RN | 21.939726 | 0 | 0.5617841 | 0.1383705 | 0.02006372 | 0.03566883 | 0.2707393 | 0.2384464 | 0.05433334 | 0.1411075 | 0.566505 | 0.02478915 | 3.580417 | 0.06687906 | 0.4328353 |
| TCGA-B6-A0IA | 22.9890411 | 0 | 0.4453135 | 0 | 0.03180811 | 0 | 0.2146089 | 0.04200245 | 0.0646032 | 0.05592634 | 0.2763419 | 0.09824898 | 5.943018 | 0.05301351 | 0.2940846 |
| TCGA-B6-A0I5 | 23.4410959 | 0 | 0.5725078 | 0.08099138 | 0 | 0.1174375 | 0.2122364 | 0.05815337 | 0.1192595 | 0 | 0.6695531 | 0.2176448 | 2.940921 | 0.1223307 | 0.06786115 |
