# Supplementary material for: Identification of N6-Methyladenosine-Related LncRNAs for Predicting Overall Survival and Clustering of a Potentially Novel Molecular Subtype of Breast Cancer
Source: Front Oncol. 2021 Oct 15;11:742944. doi: 10.3389/fonc.2021.742944 (PMC8554333; doi:10.3389/fonc.2021.742944)
Supplement: Supplementary Table 2 — Survival message and expression levels of m6A-LPS of BRCA samples belong to testing set from TCGA dataset. [file Table_2.docx]

| id | Futime(years) | fustat | AL136531.1 | LRRC8C-DT | AL138789.1 | COL4A2-AS1 | AC018926.2 | AL513190.1 | AL021578.1 | ZBTB40-IT1 | AC005104.1 | AC004846.2 | OTUD6B-AS1 | AL592301.1 | ZNF197-AS1 |
| --- | --- | --- | --- | --- | --- | --- | --- | --- | --- | --- | --- | --- | --- | --- | --- |
| TCGA-A8-A06Z | 0.084931507 | 0 | 0.542595 | 0.04157816 | 0.1130406 | 0.04019223 | 0.2832826 | 0.1641966 | 0.198977 | 0.2782539 | 0.4173808 | 0.05586564 | 12.44474 | 0.07536042 | 0.01741878 |
| TCGA-AC-A8OQ | 0.093150685 | 0 | 0.943921641 | 0.409876574 | 0.157320273 | 0.233067072 | 0.606535994 | 0.553975103 | 0.213014757 | 1.014225939 | 1.765403973 | 0.097186269 | 3.480995765 | 0.233067072 | 0.121209841 |
| TCGA-D8-A27P | 0.134246575 | 0 | 0.4386005 | 0.07332924 | 0.01993639 | 0.1063274 | 0.1152948 | 0.289585 | 0.404914 | 0.7711658 | 0.9526166 | 0.04926366 | 5.121552 | 0.04430309 | 0.1843236 |
| TCGA-BH-A0HL | 0.197260274 | 0 | 0.1326822 | 0.274515 | 0.1824381 | 0.1179398 | 0.1918297 | 0.5475196 | 0.336852 | 0.4082531 | 0.8285135 | 0.08196586 | 5.853122 | 0.03685618 | 0.3577948 |
| TCGA-AN-A04A | 0.246575342 | 0 | 0.2955602 | 0.0543559 | 0.01477801 | 0 | 0.1424387 | 0.03902858 | 0.08003887 | 0.05196663 | 0.06419407 | 0.05477564 | 3.883747 | 0.03284002 | 0.204947 |
| TCGA-BH-A0DQ | 0.268493151 | 0 | 0.6191405 | 0.1779139 | 0.03869628 | 0.2063802 | 0.3729762 | 0.204393 | 0.1571866 | 0.5442993 | 0.4202311 | 0 | 3.792738 | 0.08599173 | 0.1788849 |
| TCGA-E2-A1IL | 0.323287671 | 0 | 0.3395911 | 0.1115242 | 0.0242565 | 0.129368 | 0.2805572 | 0.5124896 | 0.6240319 | 1.364762 | 1.580514 | 0.08990828 | 3.084681 | 0 | 0.5980416 |
| TCGA-BH-A18G | 0.408219178 | 0 | 0.05269367 | 0.02422697 | 0.02634683 | 0.04683882 | 0.3047345 | 0.1391635 | 0.1783707 | 0.463241 | 0.05722389 | 0.3906249 | 2.437115 | 0.1463713 | 0.121796 |
| TCGA-A2-A0CU | 0.432876712 | 1 | 0.3423899 | 0.1180655 | 0.05135848 | 0.09130397 | 0 | 0.2034559 | 0.5563232 | 0.3010021 | 0.5949218 | 0.0634545 | 4.214661 | 0 | 0.1582798 |
| TCGA-AR-A0TR | 0.438356164 | 1 | 0.555057 | 0.08058904 | 0.07303381 | 0.05193516 | 0.08447285 | 0.5400685 | 0.3362234 | 0.3081866 | 0.5710517 | 0.1263288 | 4.816513 | 0.01622974 | 0.2926042 |
| TCGA-AN-A0XW | 0.465753425 | 0 | 0.435732832 | 0.200336934 | 0.108933208 | 0 | 0.629975179 | 0.095897185 | 0.172080667 | 0.191530915 | 0.512626861 | 0.089726195 | 3.196389289 | 0 | 0.111905703 |
| TCGA-AC-A3EH | 0.539726027 | 1 | 0.5409692 | 0.1476784 | 0.01690529 | 0.03005385 | 0.3258851 | 0.1785868 | 0.1144505 | 0.1188943 | 0.3304563 | 0.1462079 | 4.440107 | 0.05635096 | 0.1823492 |
| TCGA-AN-A0FK | 0.583561644 | 0 | 0.105945 | 0.1298943 | 0.03531501 | 0.03139112 | 0.1361543 | 0 | 0.2151775 | 0.1241847 | 0.1917557 | 0.04363244 | 2.993048 | 0 | 0.08162699 |
| TCGA-AN-A0FT | 0.58630137 | 0 | 0.7043814 | 0.08481878 | 0 | 0.1192603 | 0.03232961 | 0.1771681 | 1.021871 | 0.0589749 | 0.4735337 | 0.1657673 | 5.433699 | 0.01863443 | 0.1292147 |
| TCGA-B6-A400 | 0.589041096 | 0 | 0.5089418 | 0.2859954 | 0.1413727 | 1.45771 | 0.6813143 | 0.280023 | 0.2871322 | 0.09942696 | 1.043983 | 0.1921359 | 5.420581 | 0.01570808 | 0.2614147 |
| TCGA-E2-A56Z | 0.690410959 | 0 | 1.036031 | 0.06946567 | 0.02158397 | 0 | 0.249646 | 0.1425077 | 0.4968272 | 0.3035988 | 0.5156714 | 0.1066698 | 4.710328 | 0.02398219 | 0 |
| TCGA-A2-A0T2 | 0.698630137 | 1 | 0.5342361 | 0.1403576 | 0 | 0.06783951 | 0.07356091 | 0.3275333 | 0.1808416 | 0.134188 | 0.4144042 | 0.04714715 | 2.542203 | 0.04239969 | 0.02940075 |
| TCGA-EW-A1IY | 0.706849315 | 0 | 0.543509 | 0.3311032 | 0.0543509 | 0.07246787 | 0.4976709 | 0.1614827 | 0.2023785 | 0.143343 | 1.032913 | 0.1175155 | 5.188164 | 0 | 0.5443815 |
| TCGA-E2-A1LK | 0.728767123 | 1 | 1.24893 | 0.1827066 | 0 | 0 | 0.1094353 | 0.3748195 | 0.4612025 | 0.1996292 | 0.3082509 | 0.07013997 | 15.94875 | 0.4100022 | 0.4373897 |
| TCGA-E2-A15K | 0.753424658 | 0 | 0.554784876 | 0.092004221 | 0.110378556 | 0.067752509 | 0.45512595 | 0.408595622 | 0.213598796 | 0.482122867 | 0.785841671 | 0.282882106 | 3.949719474 | 0.162628821 | 0.234904382 |
| TCGA-A7-A6VW | 0.780821918 | 0 | 0.8070541 | 0.1948062 | 0 | 0.07173814 | 0.3889417 | 0.7726405 | 0.3278302 | 0 | 0.1752878 | 0.1994265 | 2.949394 | 0.08967268 | 0.341994 |
| TCGA-E2-A15O | 0.791780822 | 0 | 0.8318772 | 0.08052048 | 0.04378301 | 0.1556729 | 0.6330074 | 0.2601686 | 0.5335475 | 0.3849056 | 1.616603 | 0.1622845 | 10.44322 | 0.02432389 | 0.1011999 |
| TCGA-BH-A0E6 | 0.802739726 | 0 | 0.7971485 | 1.007889 | 0.05978614 | 0.1771441 | 0.4610015 | 0.1315788 | 0.02698387 | 0 | 0.519409 | 0.07386704 | 2.203202 | 0.02214301 | 0 |
| TCGA-PL-A8LZ | 0.82739726 | 0 | 0.4551486 | 0.3243588 | 0.1820594 | 0.5259495 | 0.8773949 | 0.09015323 | 0.3697681 | 0.3201045 | 0.7414185 | 0.1405864 | 2.272334 | 0.0758581 | 0.2454734 |
| TCGA-C8-A1HL | 0.868493151 | 0 | 0.2455689 | 0.2483915 | 0.07367066 | 0.1746268 | 0.8520944 | 0.09728176 | 0.2327536 | 0.3454156 | 1.333406 | 0.273065 | 2.758022 | 0.02728543 | 0.1135214 |
| TCGA-BH-A42T | 0.876712329 | 1 | 0.6204495 | 0.1996849 | 0.1240899 | 0.02757554 | 1.345553 | 0.1638601 | 0.2520303 | 0.05454502 | 0.5727227 | 0.05749339 | 4.004579 | 0.01723471 | 0.1195087 |
| TCGA-A8-A07P | 0.915068493 | 0 | 0.2266604 | 0.1116554 | 0.03238006 | 0.05756455 | 0.1248388 | 0.1710309 | 0.1534513 | 0.113864 | 0.4922941 | 0 | 4.386747 | 0.01798892 | 0.1247386 |
| TCGA-A7-A5ZX | 0.920547945 | 0 | 0.8320038 | 0.5259794 | 0 | 0 | 0.2004828 | 0.6317278 | 0.2253099 | 0 | 1.807067 | 0.1798927 | 4.735319 | 0 | 0.6730817 |
| TCGA-A7-A3J1 | 0.939726027 | 0 | 1.093281 | 0.4188814 | 0.09811492 | 0.1495084 | 0.2161568 | 0.7033272 | 1.13871 | 0.98577 | 1.36993 | 0.1212231 | 3.067797 | 0.04672139 | 0.1511883 |
| TCGA-4H-AAAK | 0.953424658 | 0 | 0.341615641 | 0.225780452 | 0 | 0.11387188 | 0.411585109 | 0.197357041 | 0.636012194 | 0.300321442 | 0.556477967 | 0.105518345 | 3.854475026 | 0.071169925 | 0.164501914 |
| TCGA-C8-A12L | 0.994520548 | 0 | 0.1171806 | 0.06285547 | 0.4491921 | 0 | 0.5270772 | 0.2063152 | 0.2379972 | 0.4807408 | 0.5090196 | 0.1447791 | 2.7894 | 0.1302006 | 0.3611343 |
| TCGA-A8-A09B | 1 | 0 | 0.5050412 | 0.1470618 | 0.03366941 | 0.1496418 | 0.4867867 | 0.3112221 | 0.2963288 | 0.5327907 | 1.243178 | 0.1039982 | 3.356519 | 0.01870523 | 0.2853523 |
| TCGA-BH-A1EV | 1 | 1 | 0.8798468 | 0.04045273 | 0.05279081 | 0.06256689 | 0.03392181 | 0.3717867 | 0.0953064 | 0.1237587 | 0.5732939 | 0.1304483 | 5.602907 | 0.0391043 | 0.325388 |
| TCGA-A8-A097 | 1 | 0 | 0.7197334 | 0.2225097 | 0.186138 | 0.08824318 | 0.4545055 | 0.03277257 | 0.1008138 | 0.08727348 | 0.5390421 | 0.1379864 | 2.30168 | 0.05515199 | 0.4206778 |
| TCGA-EW-A1IW | 1.016438356 | 0 | 0.3612419 | 0.2108043 | 0.01389392 | 0.0988012 | 0.3214015 | 0.1651217 | 0.3574402 | 0.1465732 | 0.4224757 | 0.1029974 | 3.682478 | 0 | 0.1926861 |
| TCGA-C8-A1HE | 1.02739726 | 0 | 0.5931101 | 0.1859279 | 0.0539191 | 0.09585618 | 0.7795533 | 0.1423998 | 0.2737782 | 0.1896056 | 0.6441015 | 0.09992729 | 4.111096 | 0 | 0.2700282 |
| TCGA-C8-A1HO | 1.02739726 | 0 | 0.5199363 | 0.06327825 | 0.1987992 | 0.1631173 | 0.5011434 | 0.12116 | 0.269178 | 0.2688746 | 0.863562 | 0.03778778 | 8.428676 | 0 | 0.2827715 |
| TCGA-C8-A1HM | 1.02739726 | 0 | 0.9778357 | 0.2360293 | 0.2444589 | 0 | 0.7068692 | 0.3550875 | 0.297902 | 0.08596358 | 0.690237 | 0.1812205 | 5.709819 | 0.05432421 | 0.03766943 |
| TCGA-C8-A26X | 1.030136986 | 0 | 0.1999451 | 0.2390148 | 0.01999451 | 0 | 0.4239799 | 0.2112212 | 0.3248755 | 0.07031035 | 0.4342698 | 0.1729255 | 3.433904 | 0.1332967 | 0.09243046 |
| TCGA-LL-A7T0 | 1.030136986 | 0 | 1.084867 | 0.130119 | 0.07075221 | 0.04192723 | 0.4546327 | 0.4671397 | 0.6386659 | 0.331732 | 1.07569 | 0.378802 | 4.472378 | 0.05240904 | 0.1090243 |
| TCGA-C8-A137 | 1.038356164 | 0 | 0.242390971 | 0.04457765 | 0.121195486 | 0 | 0 | 0.352083722 | 0 | 0 | 0.526460028 | 0.119791677 | 1.700004176 | 0.134661651 | 0 |
| TCGA-C8-A138 | 1.04109589 | 0 | 0.5259309 | 0.1813555 | 0.04382758 | 0.1168735 | 0.4646779 | 0.3472446 | 1.424242 | 0.154119 | 0.5711467 | 0.1895247 | 3.700509 | 0.02434865 | 0.2026058 |
| TCGA-C8-A8HQ | 1.04109589 | 0 | 0.2623519 | 0.1432381 | 0.09838196 | 0.2332017 | 0.7586078 | 0.3247822 | 0.3996333 | 0.3459585 | 1.282082 | 0.2228472 | 3.748632 | 0.1093133 | 0.1515998 |
| TCGA-C8-A26W | 1.043835616 | 0 | 0.2517541 | 0.180054 | 0.3216858 | 0.1740522 | 0.6470788 | 0.3693779 | 0.4734445 | 0.2950964 | 1.032837 | 0.2592063 | 4.229838 | 0.04662113 | 0.2586238 |
| TCGA-C8-A12Z | 1.046575342 | 0 | 0.3767636 | 0.05774154 | 0.1255879 | 0 | 0.2017476 | 0.3316763 | 0.1133656 | 0.0736046 | 0.09092333 | 0.05172215 | 6.099444 | 0.09302804 | 0.1290148 |
| TCGA-C8-A134 | 1.049315068 | 0 | 0.1775229 | 0.7957922 | 0.310665 | 0.3155962 | 1.240521 | 0.1611624 | 0.1051616 | 0.1170481 | 0.8193364 | 0.02741666 | 5.870752 | 0.01232798 | 0.1367753 |
| TCGA-C8-A132 | 1.049315068 | 0 | 0.4803495 | 0.2633216 | 0.01847498 | 0.1313776 | 0.3561442 | 0.2439612 | 0.2751704 | 0.1299339 | 1.043293 | 0.09130493 | 4.698899 | 0.02052776 | 0.2846867 |
| TCGA-C8-A12O | 1.054794521 | 0 | 0.418637237 | 0.103641403 | 0.096608593 | 0.114499073 | 0.372466865 | 0.148833046 | 0.087206629 | 0.226481684 | 0.48960011 | 0.039787323 | 7.007137504 | 0.053671441 | 0.099244783 |
| TCGA-C8-A12U | 1.054794521 | 0 | 0.4781133 | 0.08992726 | 0.1086621 | 0 | 0.9216641 | 0.1147902 | 0.5296703 | 0.0764217 | 0.3776131 | 0.1074035 | 8.329279 | 0 | 0.1674408 |
| TCGA-LL-A5YM | 1.079452055 | 0 | 0.528457746 | 0.121484539 | 0 | 0 | 1.825195426 | 0.087228239 | 0.119257037 | 0 | 0.191296921 | 0.163230192 | 1.688531988 | 0.048931273 | 0.169649357 |
| TCGA-A8-A06P | 1.084931507 | 0 | 0.40484849 | 0.16465986 | 0.0155711 | 0.11072779 | 0.09004971 | 0.37010803 | 0.147585 | 0.38328851 | 0.47347405 | 0.03847684 | 4.7862268 | 0.05190365 | 0.04798797 |
| TCGA-EW-A1J2 | 1.104109589 | 0 | 0.6245555 | 0.2469507 | 0.03747333 | 0.08882567 | 0.1685547 | 0.329889 | 0.6257888 | 0.2635487 | 1.112331 | 0.1697634 | 4.518003 | 0 | 0.3849588 |
| TCGA-D8-A1JT | 1.109589041 | 0 | 0.949137 | 0.08595459 | 0.02876173 | 0.02556598 | 0.5267208 | 0.1139392 | 0.1752478 | 0.1011401 | 0.2811101 | 0.03553573 | 10.70402 | 0 | 0.1551194 |
| TCGA-C8-A8HR | 1.117808219 | 0 | 1.064468831 | 0.489410957 | 0.133058604 | 0.157699086 | 0.641246284 | 0.20498712 | 0.360327954 | 0.623864516 | 1.252316272 | 0.10959782 | 6.866169394 | 0.024640482 | 0.273378833 |
| TCGA-C8-A131 | 1.126027397 | 0 | 0.420152 | 0.115904 | 0.0840304 | 0.07469369 | 0.1619863 | 0.2219235 | 0.284447 | 0.1477458 | 0.7756652 | 0.2855087 | 5.225575 | 0.09336711 | 0.03237126 |
| TCGA-A1-A0SJ | 1.139726027 | 0 | 0.3855753 | 0.16116 | 0.0350523 | 0.06231521 | 0.06757071 | 0.1851456 | 0.3796921 | 0.6163042 | 0.2664609 | 0.04330787 | 3.50278 | 0 | 0 |
| TCGA-A7-A4SB | 1.145205479 | 0 | 0.6121412 | 0.319823 | 0.278246 | 0.1483979 | 0 | 0.881814 | 0.7535012 | 0.4892238 | 1.813006 | 0.1375115 | 4.605298 | 0.06183245 | 0.04287579 |
| TCGA-S3-AA11 | 1.153424658 | 0 | 1.151764 | 0.08058322 | 0.2253452 | 0 | 1.110134 | 0.5290085 | 0.6441462 | 2.289221 | 2.882244 | 0.216548 | 3.124107 | 0 | 0.07716476 |
| TCGA-S3-AA17 | 1.161643836 | 0 | 0.783037315 | 0.360017156 | 0.043502073 | 0.193342547 | 0.545086217 | 0.919108449 | 1.443115315 | 0.458922969 | 1.700714531 | 0.134369338 | 1.692302313 | 0.024167818 | 0.033516846 |
| TCGA-A8-A09X | 1.167123288 | 1 | 0.3408671 | 0.1480138 | 0.1136224 | 0 | 0.07301035 | 0.0250063 | 0.05128231 | 0.3995511 | 0.2056513 | 0.3041628 | 4.290005 | 0.1472882 | 0.0583614 |
| TCGA-D8-A1Y2 | 1.18630137 | 0 | 0.6335467 | 0.1525783 | 0.03016889 | 0.05363358 | 0.1744707 | 0.3784598 | 0.08169855 | 0.1591326 | 0.5569641 | 0.2050087 | 5.12585 | 0.03352099 | 0.1627086 |
| TCGA-A1-A0SD | 1.197260274 | 0 | 1.274187 | 0.2697915 | 0.08382807 | 0.0894166 | 0.1939155 | 0.1771113 | 0.6810291 | 0.235824 | 0.983178 | 0.06214281 | 4.440921 | 0.03725692 | 0.2583465 |
| TCGA-AC-A3TN | 1.249315068 | 0 | 0.5028888 | 0.0990914 | 0 | 0.09578835 | 0.7270682 | 0.3083146 | 0.9241077 | 0.1894715 | 0.1560353 | 0.2662842 | 2.074538 | 0 | 0.3044317 |
| TCGA-A8-A09V | 1.252054795 | 0 | 0.9234121 | 0.09005761 | 0.4197328 | 0.04974611 | 0.1887955 | 0.8868082 | 0.530438 | 0.9347895 | 2.066377 | 0.3111533 | 4.716318 | 0.07772829 | 0.3018303 |
| TCGA-A8-A084 | 1.254794521 | 0 | 0.346135 | 0.03536501 | 0.1153783 | 0.06837235 | 0.1853467 | 0.634819 | 0.2603743 | 0.135242 | 1.044148 | 0.2375873 | 3.136663 | 0.04273272 | 0.3259485 |
| TCGA-D8-A1XF | 1.268493151 | 0 | 1.220876 | 0.04952845 | 0.1615866 | 0.03191833 | 0.1730512 | 0.1422495 | 0.2431016 | 0 | 0.623924 | 0.02218263 | 12.58918 | 0.01994896 | 0.1383299 |
| TCGA-BH-A18N | 1.282191781 | 1 | 0.3212562 | 0.5661987 | 0.1070854 | 0.03172901 | 0.1720247 | 0.1649734 | 0.2899915 | 0.4393247 | 0.6589871 | 0.1323063 | 9.677426 | 0.01983063 | 0.3025205 |
| TCGA-AC-A3W7 | 1.290410959 | 0 | 0.5735839 | 0.2081974 | 0.1056602 | 0.2146747 | 0.3491696 | 0.8371426 | 0.7766439 | 0.4246312 | 1.803122 | 0.3170388 | 3.566568 | 0.08385729 | 0.09303718 |
| TCGA-D8-A1Y0 | 1.293150685 | 0 | 0.3660427 | 0.3926895 | 0.1952228 | 0.2169142 | 0.2822498 | 0.209455 | 0.5617129 | 0.5577793 | 1.431045 | 0.06030047 | 8.908318 | 0.05422855 | 0.09400775 |
| TCGA-LL-A73Y | 1.306849315 | 0 | 0.6124834 | 0.5084472 | 0.08506713 | 0.3024609 | 0.3935636 | 1.100841 | 0.6910955 | 0.5982743 | 1.441137 | 0.189184 | 11.5056 | 0.1890381 | 0.5243303 |
| TCGA-D8-A1XR | 1.320547945 | 0 | 0.8558744 | 0.1371307 | 0.1296779 | 0.161377 | 0.2249834 | 0.3082304 | 0.1755865 | 0.2280052 | 0.732299 | 0.2082858 | 4.891377 | 0.02881732 | 0.0799299 |
| TCGA-A2-A1G6 | 1.37260274 | 0 | 0.9262033 | 0.6715179 | 0.0534348 | 0.1899904 | 0.1716781 | 0.5409631 | 0.3617589 | 0.5010736 | 1.354004 | 0.2200661 | 7.395683 | 0 | 0.7136077 |
| TCGA-D8-A1JA | 1.375342466 | 0 | 0.2300959 | 0.1057912 | 0.1406142 | 0.02272552 | 0.09856853 | 0.06752012 | 0.328863 | 0.04495158 | 0.388699 | 0 | 3.627007 | 0.0284069 | 0.1575831 |
| TCGA-AC-A3W5 | 1.380821918 | 0 | 0.07439533 | 0.2650868 | 0.01859883 | 0.06612918 | 0.2868254 | 0.3438354 | 0.3525646 | 0.4578174 | 0.3635609 | 0.275751 | 3.093182 | 0.165323 | 0.05731903 |
| TCGA-D8-A1XT | 1.38630137 | 0 | 0.8345002 | 0.1978341 | 0.1434297 | 0.09272225 | 0.1508133 | 0.1721802 | 0.1412412 | 0.4585166 | 1.387687 | 0.03222009 | 2.503917 | 0.04346355 | 0.1004615 |
| TCGA-AC-A5EH | 1.4 | 0 | 0.3558072 | 0.06361815 | 0.0593012 | 0 | 0.2286311 | 0.2088185 | 0.37471 | 0 | 0.343464 | 0.04884526 | 1.875848 | 0 | 0.1218387 |
| TCGA-A8-A075 | 1.419178082 | 0 | 0.360877252 | 0.107848374 | 0.108263176 | 0.064155956 | 0.139133398 | 0.333575672 | 0.122158731 | 0.634509454 | 0.783805796 | 0.178348603 | 7.56995438 | 0.260633571 | 0.139021734 |
| TCGA-A8-A0AB | 1.419178082 | 0 | 0.531436142 | 0.048867691 | 0.093001325 | 0.023619384 | 0.307336564 | 0.403511349 | 0.197883133 | 0.654075252 | 0.981112878 | 0.016415016 | 3.797261141 | 0.132859036 | 0.429925868 |
| TCGA-D8-A27N | 1.421917808 | 0 | 0.8922093 | 0.1025528 | 0.1189612 | 0.05287166 | 0.1719922 | 0.2159957 | 0.1208069 | 0 | 1.065807 | 0.2020963 | 2.321021 | 0.08261197 | 0.1145694 |
| TCGA-LL-A5YL | 1.421917808 | 0 | 0.484004 | 0.259619 | 0 | 0 | 0.1943791 | 0.02663021 | 0.2730629 | 0 | 0 | 0.09966621 | 2.935115 | 0.06722278 | 0.3729084 |
| TCGA-AR-A1AR | 1.435616438 | 1 | 0.3246704 | 0.1779802 | 0.4870056 | 0.06659905 | 0.2407195 | 0.3792573 | 0.1183566 | 0.3952032 | 0.8136536 | 0.2468536 | 4.293195 | 0.09712362 | 0.1346947 |
| TCGA-S3-AA15 | 1.438356164 | 0 | 1.100458122 | 0.899480457 | 0.122273125 | 0.54343611 | 1.237462949 | 0.847670631 | 0.082780113 | 0.644957141 | 1.593423525 | 0.151071042 | 2.60295858 | 0.067929514 | 0.188414446 |
| TCGA-E2-A15D | 1.44109589 | 0 | 0.990829656 | 0.260316494 | 0.10616032 | 0.188729458 | 0.204646401 | 0.280368384 | 0.14374317 | 0.933277541 | 1.652450235 | 0.065581665 | 4.672538113 | 0.019659319 | 0.190850014 |
| TCGA-S3-AA14 | 1.449315068 | 0 | 0.7759253 | 0.1664821 | 0.2327776 | 0.2758845 | 0.4985865 | 0.1366141 | 0.5253091 | 0.9095095 | 0.7864582 | 0.127823 | 2.144544 | 0 | 0.07970982 |
| TCGA-D8-A1X6 | 1.482191781 | 0 | 0.4373731 | 0.1131137 | 0.1093433 | 0.07289551 | 0.1053911 | 0.1263389 | 0.03701324 | 0.1922519 | 0.3562315 | 0.06754797 | 15.83976 | 0.0455597 | 0.06318391 |
| TCGA-A8-A093 | 1.495890411 | 0 | 0.4738819 | 0.07262557 | 0.04738819 | 0 | 0.2436019 | 0.5214656 | 0.2566582 | 0.3332796 | 0.4116983 | 0.05854911 | 2.856698 | 0.07020472 | 0.09736257 |
| TCGA-A2-A04P | 1.501369863 | 1 | 1.347912827 | 0.088532862 | 0.019255898 | 0.171163534 | 0.742396049 | 0.33055516 | 0.80825883 | 0.067713046 | 1.212860152 | 0.309283914 | 2.17804101 | 0.171163534 | 0.029672009 |
| TCGA-E2-A15G | 1.517808219 | 0 | 0.8584386 | 0.1246372 | 0.04518098 | 0.1204826 | 0.04354793 | 0.805427 | 0.4588195 | 0.4766345 | 2.502331 | 0.1674662 | 3.381693 | 0.1004022 | 0.4177246 |
| TCGA-A2-A0YE | 1.517808219 | 0 | 0.321186 | 0.1107538 | 0.1445337 | 0.05709974 | 0.2167038 | 0.1696498 | 0.1087231 | 0.1129445 | 1.151038 | 0.09920804 | 3.93056 | 0.053531 | 0.2969553 |
| TCGA-E2-A14Z | 1.542465753 | 1 | 0.5979793 | 0.3436663 | 0.2562768 | 0.07593388 | 0.3293518 | 0.3948144 | 0.08675097 | 0.2252983 | 0.5102344 | 0.05277258 | 4.609486 | 0.04745867 | 0.1974525 |
| TCGA-EW-A1J1 | 1.575342466 | 0 | 0.3753664 | 0.1109457 | 0.01340594 | 0.09533114 | 0.2842706 | 0.3009422 | 0.3085825 | 0.2828506 | 0.6114565 | 0.1987599 | 5.966882 | 0 | 0.1032881 |
| TCGA-D8-A1JE | 1.575342466 | 0 | 1.176119 | 0.1228965 | 0.06682495 | 0.07127995 | 0.1030553 | 0.05294522 | 0.1447717 | 0.1409933 | 0.6095887 | 0.09907638 | 4.737999 | 0.02969998 | 0.08237811 |
| TCGA-A8-A07F | 1.580821918 | 0 | 0.3870798 | 0.1368983 | 0.1935399 | 0.105868 | 0.2008941 | 0.2948867 | 0.2620568 | 0.523523 | 0.4203582 | 0.1471524 | 3.101514 | 0.01654187 | 0.04588179 |
| TCGA-BH-A1FG | 1.580821918 | 1 | 0.2545604 | 0.05851964 | 0 | 0 | 0.3680392 | 0.02100911 | 0.2154249 | 0.167842 | 0.1036671 | 0.1179431 | 10.15192 | 0.1060668 | 0.1716138 |
| TCGA-A8-A07G | 1.580821918 | 0 | 0.6226357 | 0.1295028 | 0.05929864 | 0.02635495 | 0.1428883 | 0.07830357 | 0.1405102 | 0.156392 | 0.5795704 | 0.03663237 | 2.948649 | 0.01647185 | 0.1370627 |
| TCGA-AQ-A7U7 | 1.6 | 1 | 1.411209 | 0.6488316 | 0.04032025 | 0.3942424 | 0.699532 | 0.4791842 | 0.9554022 | 0.8507129 | 2.714775 | 0.3487157 | 3.269101 | 0.1120007 | 0.3106536 |
| TCGA-S3-AA10 | 1.605479452 | 0 | 0.2546135 | 0.4389887 | 0.08487115 | 0.2640436 | 0.6135264 | 0.2521619 | 0.5745861 | 0.447672 | 1.566852 | 0.2359353 | 4.081273 | 0.02357532 | 0.1961709 |
| TCGA-AC-A2QI | 1.610958904 | 0 | 0.8646098 | 0.1510583 | 0.0172922 | 0.03074168 | 0.0666687 | 0.2283426 | 0.1170699 | 0.1216154 | 0.638481 | 0.1281892 | 3.784772 | 0.0384271 | 0.2398147 |
| TCGA-AC-A5XS | 1.610958904 | 0 | 0.8015688 | 0.4660914 | 0 | 0 | 0.318128 | 0.2490512 | 0.6384353 | 0 | 0.870482 | 0.2038967 | 3.69111 | 0.02619506 | 0 |
| TCGA-A2-A1G4 | 1.630136986 | 0 | 1.182885 | 0.3383989 | 0.02628634 | 0.09346255 | 0.5320609 | 0.1561994 | 0.1957572 | 0.5546129 | 0.8849339 | 0.08119333 | 5.639949 | 0.01460352 | 0.2835381 |
| TCGA-OL-A6VQ | 1.643835616 | 0 | 0.313299572 | 0.28809156 | 0 | 0 | 0.503292485 | 0.068951763 | 1.767557528 | 0.918093983 | 1.020704487 | 0.193544137 | 3.23675111 | 0.058018439 | 0.080462169 |
| TCGA-D8-A1XL | 1.660273973 | 0 | 1.900776 | 0.08739198 | 0 | 0.08447892 | 0.3053455 | 0.167331 | 0.1286844 | 0.05570038 | 0.1720159 | 0.09785203 | 6.538997 | 0.03519955 | 0.09763213 |
| TCGA-EW-A1PB | 1.665753425 | 0 | 0.4724411 | 0.8326548 | 0.01574804 | 0.111986 | 0.7892992 | 0.1455664 | 0.04264631 | 0.1107554 | 0.3420388 | 0.07782814 | 4.536189 | 0.05249346 | 0.2426664 |
| TCGA-A8-A07E | 1.665753425 | 0 | 0.2503753 | 0.1918585 | 0.08345843 | 0.09891369 | 0.02681395 | 0.1285742 | 0.0941703 | 0.09782673 | 0.5135903 | 0.1374862 | 2.911114 | 0.04636579 | 0.4286789 |
| TCGA-D8-A1JJ | 1.673972603 | 0 | 0.3773063 | 0.06672084 | 0.2612121 | 0.1031949 | 0.2237962 | 0.2107901 | 0.1964924 | 0.3572131 | 1.197715 | 0.1075777 | 8.587382 | 0.03224841 | 0.1118083 |
| TCGA-AR-A2LH | 1.687671233 | 1 | 0.5460722 | 0.5021353 | 0.02730361 | 0.04853975 | 0.2105339 | 0.3605428 | 0.3327266 | 0.4800635 | 0.5930196 | 0.2024051 | 3.908383 | 0.09101203 | 0.04207298 |
| TCGA-C8-A26V | 1.687671233 | 0 | 0.2464271 | 0.1596497 | 0.05600615 | 0.03982659 | 0.1727419 | 0.02958234 | 0.1061668 | 0.1575558 | 0.4135839 | 0.06919679 | 2.965222 | 0.1120123 | 0.2071239 |
| TCGA-A2-A1G0 | 1.687671233 | 0 | 1.021632 | 0.2715546 | 0.04788902 | 0.1418934 | 0.09231619 | 0.2529489 | 1.12394 | 1.066539 | 1.005453 | 0.09861317 | 7.043807 | 0.01773668 | 0.3197726 |
| TCGA-D8-A141 | 1.715068493 | 0 | 0.602155339 | 0.305995453 | 0.047538579 | 0.08451303 | 0.18328127 | 0.334797148 | 0.085824234 | 0.501505892 | 1.376682842 | 0.117469849 | 3.410056458 | 0 | 0.195343119 |
| TCGA-E2-A15E | 1.726027397 | 0 | 0.468799822 | 0.147817407 | 0.126579058 | 0.061912136 | 0.170427732 | 0.520070233 | 0.513287196 | 0.720724761 | 0.824768625 | 0.12495148 | 6.066090776 | 0.108653891 | 0.268319039 |
| TCGA-A2-A0T7 | 1.728767123 | 0 | 0.8738936 | 0.3080391 | 0.07282446 | 0 | 0.1123076 | 0.09616435 | 0.256375 | 0.2048688 | 0.2530732 | 0.03599047 | 5.90798 | 0.01618321 | 0.1571045 |
| TCGA-BH-A42V | 1.739726027 | 0 | 0.5662653 | 0.4453387 | 0.01490172 | 0.1059678 | 1.407584 | 0.5903295 | 0.7465572 | 0.5240165 | 1.262263 | 0.1841139 | 4.839971 | 0.01655747 | 0.2755502 |
| TCGA-A1-A0SI | 1.739726027 | 0 | 0.829003745 | 0.133402902 | 0.055266916 | 0.098252296 | 0.106538634 | 0.456123106 | 0.430286852 | 0.340104101 | 0.570174522 | 0.119496035 | 2.085960497 | 0 | 0.596137525 |
| TCGA-D8-A13Z | 1.739726027 | 0 | 0.437884031 | 0.163577368 | 0.123154884 | 0.048653781 | 0.316542673 | 0.289111877 | 0.055584715 | 0.192476497 | 1.040222245 | 0.135253755 | 2.385079769 | 0.030408613 | 0.147601359 |
| TCGA-B6-A0WX | 1.750684932 | 1 | 0.7131649 | 0.1967352 | 0.1248039 | 0.06339244 | 1.065451 | 0.4473222 | 0.3138328 | 0 | 1.122993 | 0.3744804 | 4.246945 | 0.138671 | 0.5769424 |
| TCGA-A7-A4SE | 1.764383562 | 0 | 0.637846489 | 0.293262754 | 0.289930222 | 0.721604109 | 0.16767049 | 0.30628117 | 0.706628243 | 0.305860454 | 1.19645413 | 0.322393452 | 2.774529457 | 0.161072346 | 0.223381231 |
| TCGA-AO-A0J3 | 1.783561644 | 0 | 0.2794871 | 0.08761352 | 0.02540792 | 0.02258482 | 0.391833 | 0.2851838 | 0.8600706 | 0.3127129 | 0.3311077 | 0.09417607 | 5.13689 | 0 | 0.254487 |
| TCGA-BH-A18H | 1.78630137 | 0 | 0.303915 | 0.1746638 | 0.07597876 | 0.04502445 | 0.3905735 | 0.4013183 | 0.6515527 | 0.8905935 | 1.210159 | 0.1251645 | 3.795271 | 0.1407014 | 0.07805201 |
| TCGA-AO-A0JA | 1.794520548 | 0 | 0.3791284 | 0.1980817 | 0.05169933 | 0.09190992 | 0.2325432 | 0.3185873 | 0.3266756 | 0.2423998 | 0.8234464 | 0.04258375 | 2.921111 | 0.0191479 | 0.0796651 |
| TCGA-D8-A4Z1 | 1.805479452 | 0 | 1.078976 | 0.6788473 | 0 | 0.1009568 | 0.3284136 | 0.8248739 | 0.6535848 | 0.5990842 | 2.281806 | 0.5963856 | 5.452574 | 0.09464698 | 0.13126 |
| TCGA-A7-A26I | 1.810958904 | 0 | 7.748275 | 2.814756 | 1.434866 | 1.445494 | 4.056809 | 5.431569 | 4.27424 | 5.045682 | 5.505729 | 3.486521 | 1.818595 | 7.121186 | 1.695122 |
| TCGA-BH-A8G0 | 1.81369863 | 0 | 1.276029 | 0.2824755 | 0 | 0.1260275 | 0.5010733 | 0.5928668 | 0.2239699 | 0.3323803 | 1.64235 | 0.3211512 | 4.009223 | 0.05251147 | 0.327712 |
| TCGA-A2-A0YG | 1.824657534 | 0 | 0.3321173 | 0.04580928 | 0.2989055 | 0.1180861 | 0.1280452 | 0.2192796 | 0.4272087 | 0.1751827 | 0.5049385 | 0 | 4.427788 | 0 | 0.1279424 |
| TCGA-E2-A1IN | 1.849315068 | 0 | 0.4438586 | 0.1247112 | 0.0123294 | 0.04383788 | 0.1188376 | 0.1302474 | 0.3338852 | 0.1734246 | 0.6962487 | 0.1370992 | 4.918466 | 0.02739868 | 0.2849814 |
| TCGA-E9-A1N6 | 1.857534247 | 1 | 0.08700431 | 0.04000198 | 0.3915194 | 0 | 0.08385958 | 0.1148888 | 0.08835417 | 0.3824365 | 0.283453 | 0.02687392 | 2.849467 | 0.02416786 | 0.03351691 |
| TCGA-A2-A1FZ | 1.871232877 | 0 | 0.5089922 | 0.1560129 | 0.04241602 | 0.1508125 | 0.1635316 | 0.1960355 | 0.5168891 | 0.7457761 | 1.105503 | 0.1048118 | 3.110367 | 0 | 0.09804023 |
| TCGA-AC-A23E | 1.912328767 | 0 | 0.2399394 | 0.07354465 | 0.02665994 | 0.07109316 | 0.2055706 | 0.1408173 | 0.07219616 | 0.3749969 | 0.4053275 | 0.01646946 | 3.256159 | 0.02962215 | 0.1232434 |
| TCGA-BH-A0WA | 1.920547945 | 0 | 1.933191 | 0.4755984 | 0.03391562 | 0.1205889 | 0 | 0.1343563 | 0.1836897 | 0.1192637 | 0.6261346 | 0.1047587 | 7.444843 | 0.01884201 | 0.3397006 |
| TCGA-E2-A153 | 1.936986301 | 0 | 0.5335584 | 0.2341636 | 0.02425265 | 0 | 0.467521 | 0.09607654 | 0.3612243 | 0.5969884 | 0.8428072 | 0.1498233 | 3.390286 | 0.02694739 | 0.22423 |
| TCGA-E2-A15A | 1.945205479 | 0 | 0.4175705 | 0.200066225 | 0.104580865 | 0.07166274 | 0.2014202 | 0.12705491 | 0.4469519 | 0.2263374 | 0.60959165 | 0.0791751 | 6.411643 | 0.04468464 | 0.2113953 |
| TCGA-EW-A424 | 1.95890411 | 0 | 1.401185 | 0.3294321 | 0.06369021 | 0 | 0.7980459 | 0.3784617 | 0.4958673 | 0.1679742 | 1.383317 | 0.3541077 | 3.958141 | 0.05307517 | 0.3434977 |
| TCGA-A2-A0YT | 1.980821918 | 1 | 0.3635982 | 0.1028748 | 0.15383 | 0.02486142 | 0.4313306 | 0 | 0.3408364 | 0.1475293 | 0.4556052 | 0.1036692 | 11.54846 | 0.01553839 | 0.1077461 |
| TCGA-A7-A26H | 1.983561644 | 0 | 0.2128353 | 0.1359101 | 0.09459346 | 0.04204154 | 0.113968 | 0.1873653 | 0.1440916 | 0.4989545 | 0.796126 | 0.07304514 | 5.583989 | 0.05255192 | 0.1639822 |
| TCGA-E9-A54Y | 1.98630137 | 0 | 1.023751 | 0.03362074 | 0 | 0 | 0.1879521 | 0.3218712 | 0.9901283 | 0 | 0.05294125 | 0.2108111 | 3.391817 | 0.08125012 | 0.07512049 |
| TCGA-E2-A156 | 1.989041096 | 0 | 0.6532823 | 0.1314074 | 0.04083015 | 0 | 0.2361262 | 0.2695801 | 0.4422786 | 0.7178927 | 0.7981278 | 0.1513395 | 3.933392 | 0.04536683 | 0.3145822 |
| TCGA-D8-A1X9 | 1.991780822 | 0 | 0.6473084 | 0.1602532 | 0.08713767 | 0 | 0.09598642 | 0.2958811 | 0.2696826 | 0.3064182 | 0.7840701 | 0.04614019 | 8.096141 | 0.04149413 | 0.2685463 |
| TCGA-BH-A0HF | 1.991780822 | 0 | 0.7110102 | 0.1826801 | 0.08364826 | 0.037177 | 0.2418745 | 0.4142142 | 0.1698921 | 0.07353693 | 0.09083974 | 0.0258373 | 2.542439 | 0 | 0.8378253 |
| TCGA-B6-A0I8 | 2.052054795 | 1 | 0.3608723 | 0.04839284 | 0.03007269 | 0.05346256 | 0 | 0.1786988 | 0.4275497 | 0 | 0.6531625 | 0 | 4.280742 | 0.1503635 | 0 |
| TCGA-OL-A5RY | 2.060273973 | 0 | 0.4072636 | 0.5383369 | 0 | 0.04525151 | 0.2944074 | 0.436954 | 0.379117 | 0.2685254 | 0.7187004 | 0.1886935 | 3.578551 | 0.02828219 | 0.07844563 |
| TCGA-AQ-A04H | 2.065753425 | 0 | 0.4738385 | 0.1021204 | 0.1332671 | 0.3422167 | 0.02854449 | 0.1955317 | 2.145305 | 0.624842 | 1.447244 | 0.07317969 | 5.396034 | 0.04935818 | 0.04563453 |
| TCGA-AC-A3TM | 2.087671233 | 0 | 0.7309693 | 0.2147164 | 0 | 0 | 1.135106 | 0.2949349 | 0.4398875 | 0 | 0.1323021 | 0.1505213 | 2.004722 | 0.09024312 | 0.1564407 |
| TCGA-A7-A13F | 2.095890411 | 0 | 0.265646 | 0.1483081 | 0.1517977 | 0.03373282 | 0.3657776 | 0.05011204 | 0.8992248 | 0.06672427 | 0.0824241 | 0.02344366 | 2.785863 | 0 | 0.05847745 |
| TCGA-OL-A66N | 2.169863014 | 0 | 0.3456155 | 0.3354633 | 0 | 0 | 0.3331233 | 0.1774825 | 1.299917 | 0.06751951 | 0.2502193 | 0.1660615 | 3.269994 | 0 | 0.2366976 |
| TCGA-E9-A5UP | 2.2 | 0 | 0.6342994 | 0.06804745 | 0 | 0.07517623 | 0.3260656 | 0.1675179 | 0.1145139 | 0.07435011 | 0.5510656 | 0.1044921 | 5.999085 | 0.04698514 | 0.06516077 |
| TCGA-BH-A18L | 2.221917808 | 1 | 0.2375283 | 0.07508077 | 0.1929917 | 0.05278406 | 0.4578858 | 0.2156378 | 0.502528 | 0.3654281 | 0.4191675 | 0.09170975 | 14.7379 | 0.03299004 | 0.1372555 |
| TCGA-AQ-A04J | 2.243835616 | 0 | 1.403211 | 0.409942 | 0.1461678 | 0.8575179 | 0.422654 | 0.5597404 | 0.7718651 | 0.5653964 | 1.269874 | 0.2708902 | 4.805806 | 0.1136861 | 0.1126173 |
| TCGA-E2-A2P5 | 2.249315068 | 1 | 0.3172006 | 0.1134306 | 0.03524451 | 0.0626569 | 0.3736767 | 0.2792412 | 0.2386088 | 0.06196837 | 0.8420407 | 0.130636 | 3.617709 | 0 | 0.1629281 |
| TCGA-LQ-A4E4 | 2.326027397 | 0 | 0.562623 | 0.0823064 | 0.2045902 | 0.09092897 | 0.09859768 | 0.4052405 | 0 | 0.1798595 | 2.666153 | 0.3475663 | 3.252197 | 0.0284153 | 0.3546668 |
| TCGA-E9-A1R0 | 2.356164384 | 0 | 0.2583325 | 0.2309486 | 0.02870361 | 0.02551432 | 0.1936629 | 0.4358843 | 0.2914894 | 0.3028073 | 0.4675702 | 0.05319588 | 4.594871 | 0.01594645 | 0.2653817 |
| TCGA-E2-A1IJ | 2.369863014 | 0 | 0.234706175 | 0.175355188 | 0 | 0 | 0.282778524 | 0.271187327 | 0.158898398 | 0 | 1.083259268 | 0.253736405 | 2.629459093 | 0.03259808 | 0.316457764 |
| TCGA-EW-A1J6 | 2.397260274 | 0 | 1.351871 | 0.1832776 | 0.2426436 | 0.06162377 | 0.1002314 | 0.1830912 | 0.1173372 | 0.2437863 | 0.6775826 | 0.2141366 | 4.435804 | 0.134802 | 0.2136554 |
| TCGA-E2-A1LE | 2.408219178 | 1 | 0.4587115 | 0.3251403 | 0.2102428 | 0.1019359 | 0.1105329 | 0.2271474 | 0.0517587 | 0.2688419 | 1.037809 | 0.02361449 | 3.618393 | 0 | 0.1767107 |
| TCGA-A8-A08C | 2.41369863 | 0 | 0.1712667 | 0.06299464 | 0.03425334 | 0 | 0 | 0.09046273 | 0.1159492 | 0.2409026 | 1.004351 | 0.04232073 | 4.279358 | 0.01902963 | 0.2639101 |
| TCGA-WT-AB44 | 2.419178082 | 0 | 1.242957 | 0.1428686 | 0 | 0 | 0.1996718 | 0.9118435 | 0.981743 | 0 | 0.7498985 | 0.5118998 | 1.0701 | 0.03836286 | 0.3192184 |
| TCGA-E9-A2JS | 2.476712329 | 1 | 0.6531757 | 0.0675699 | 0.2775997 | 0 | 0.3777402 | 0.06468865 | 0.7075302 | 0.1722661 | 0.4610652 | 0.06052593 | 7.195042 | 0.01814377 | 0.1761373 |
| TCGA-EW-A1P4 | 2.484931507 | 0 | 0.2430436 | 0.2234883 | 0.1041615 | 0.1234507 | 0.5354488 | 0.1604689 | 0.2350613 | 0 | 0.3393498 | 0.08579586 | 3.286575 | 0.05786752 | 0.1070038 |
| TCGA-EW-A1OY | 2.487671233 | 0 | 1.057956 | 0.06632954 | 0.04808891 | 0 | 0.1390523 | 0.09525177 | 0.7813601 | 0.08455194 | 0.3133395 | 0.2376595 | 6.222989 | 0 | 0.2223051 |
| TCGA-EW-A1P7 | 2.506849315 | 0 | 0.119599677 | 0.797331183 | 0.039866559 | 0.425243298 | 0.384255992 | 0.658044993 | 0.863681733 | 0.070095049 | 0.649410014 | 0.147767941 | 4.302324228 | 0.044296177 | 0.337873888 |
| TCGA-A8-A06X | 2.583561644 | 1 | 0.051053133 | 0.105627173 | 0.2297391 | 0 | 0.049207839 | 0.303369376 | 0.138253902 | 0.179527502 | 0.609865485 | 0 | 2.8873185 | 0 | 0.039334677 |
| TCGA-A7-A13D | 2.643835616 | 0 | 3.474067267 | 1.401839567 | 0.91937416 | 1.3089576 | 1.6212311 | 1.840080367 | 4.1382569 | 4.964386433 | 4.293953233 | 0.787630547 | 6.187716333 | 3.909946433 | 0.801219 |
| TCGA-A2-A0YM | 2.643835616 | 0 | 0.2714592 | 0.3328235 | 0.3770266 | 0.02681078 | 0.2035035 | 0.2987171 | 0.1633605 | 0.3181939 | 0.7533708 | 0.1117978 | 6.840162 | 0.1340539 | 0.4182998 |
| TCGA-A2-A0EV | 2.652054795 | 0 | 0.2747918 | 0.1342374 | 0.1030469 | 0 | 0.1655373 | 0.2494671 | 0.09301839 | 0.1811814 | 0.4849268 | 0.2758528 | 3.177324 | 0.0572483 | 0.0264647 |
| TCGA-A8-A07L | 2.671232877 | 0 | 0.107041716 | 0.172251038 | 0.17840286 | 0.031716064 | 0.206345477 | 0.047116024 | 0.458965892 | 0.125470144 | 0.348733193 | 0 | 13.0081782 | 0.01982254 | 0.247415845 |
| TCGA-E2-A1B5 | 2.695890411 | 0 | 0.3097931 | 0.6854617 | 0 | 0 | 0.1492979 | 0.1534051 | 0.419466 | 0 | 0.3784802 | 0.3349114 | 6.047384 | 0 | 0.02983561 |
| TCGA-BH-A18F | 2.742465753 | 0 | 0.3369335 | 0.3291879 | 0.06317503 | 0.07487411 | 0.0405944 | 0.1112298 | 0.5987817 | 0.1481026 | 0.1372127 | 0.02601803 | 4.42404 | 0.07019448 | 0.1297978 |
| TCGA-A8-A08S | 2.750684932 | 0 | 0.2094571 | 0.03439361 | 0 | 0.1063909 | 0.1442045 | 0.2765871 | 0.2228357 | 0.2630544 | 0.3899395 | 0.07393963 | 6.193035 | 0.1329886 | 0.2305421 |
| TCGA-A2-A0D2 | 2.81369863 | 0 | 0.873555375 | 0.627554149 | 0.127393492 | 0.517662445 | 1.298054172 | 0.216286063 | 0.221777105 | 0.255986923 | 0.790547851 | 0.134912027 | 2.299824603 | 0 | 0.112174045 |
| TCGA-A2-A3XS | 2.82739726 | 1 | 0.548089 | 0.541048 | 0.1773229 | 0.2579243 | 0.2175265 | 0.1915806 | 0.2182716 | 0.3401199 | 1.085383 | 0.1593354 | 5.807481 | 0.03582281 | 0.1987217 |
| TCGA-C8-A3M7 | 2.832876712 | 1 | 0.7167485 | 0.5492325 | 0.01706544 | 0.09101568 | 0.1644862 | 0.270418 | 0.3466041 | 0.2400413 | 1.037826 | 0.1897624 | 5.43913 | 0.0189616 | 0.31556 |
| TCGA-A8-A07C | 2.832876712 | 0 | 0.4496027 | 0.2985867 | 0.4620917 | 0.3108365 | 0.07222534 | 0.1484246 | 0.0169103 | 0.5270069 | 0.5425071 | 0.07715191 | 9.395592 | 0.09713639 | 0.4426265 |
| TCGA-EW-A3E8 | 2.835616438 | 0 | 1.10763 | 0.4129098 | 0.05987191 | 0.07982922 | 0.7213484 | 0.3360074 | 1.763224 | 0.9474237 | 1.853049 | 0.03698651 | 3.995845 | 0.06652435 | 0.09225849 |
| TCGA-E2-A14V | 2.854794521 | 0 | 0.3846249 | 0.07368294 | 0.2884687 | 0.08547221 | 0.09268071 | 0.2327854 | 0.5424893 | 0.1690659 | 1.079038 | 0.03960102 | 5.059204 | 0.1068403 | 0 |
| TCGA-EW-A1PG | 2.879452055 | 0 | 0.906023515 | 0.367555178 | 0 | 0.047373779 | 0.051369157 | 0.140752905 | 0.793794766 | 0 | 0.405142269 | 0.197543169 | 3.635695076 | 0 | 0.451685786 |
| TCGA-E9-A1N3 | 2.901369863 | 0 | 1.27308 | 0.03658276 | 0.159135 | 0.07072666 | 0.07669156 | 0.2451598 | 0.1436479 | 0.6062285 | 0.7776733 | 0.04915367 | 4.758425 | 0.02946944 | 0.183912 |
| TCGA-BH-A18I | 2.994520548 | 0 | 0.2725484 | 0.2819466 | 0.1362742 | 0.04845304 | 0.3152366 | 0.2339342 | 0.2583251 | 0.6229677 | 1.006332 | 0.06734786 | 7.567673 | 0.03028315 | 0.08399564 |
| TCGA-A2-A04Y | 3.010958904 | 0 | 0.491666 | 0.1189754 | 0.1164472 | 0.1380115 | 0.149651 | 0.5979866 | 0.5430918 | 1.137457 | 1.405094 | 0.03197178 | 6.192947 | 0.08625719 | 0.01993746 |
| TCGA-E9-A1N9 | 3.016438356 | 0 | 0.1345848 | 0.09281709 | 0.2018772 | 0.07975395 | 0 | 0.2073383 | 0.3644609 | 0.1577551 | 0.3897478 | 0.1108549 | 9.292151 | 0.02492311 | 0.4147717 |
| TCGA-BH-A28Q | 3.065753425 | 0 | 0.8898458 | 0.1052034 | 0 | 0.09039704 | 0.2450522 | 0.1510762 | 0.3786731 | 0.2235092 | 0.8559086 | 0.09423629 | 3.714492 | 0.08474722 | 0.5680649 |
| TCGA-E9-A1N5 | 3.068493151 | 0 | 0.3039043 | 0.1863015 | 0.1772775 | 0.09004571 | 0.4881996 | 0.3678621 | 1.405932 | 0.6233934 | 1.100106 | 0.06258003 | 4.106678 | 0 | 0.1951231 |
| TCGA-A7-A0D9 | 3.120547945 | 0 | 0.6060787 | 0.2351167 | 0.05681988 | 0.2020262 | 0 | 0.6752734 | 0.5641918 | 0.3996124 | 1.110687 | 0.09360289 | 5.280117 | 0 | 0.1167407 |
| TCGA-AO-A0J6 | 3.123287671 | 0 | 0.2779606 | 0.5867089 | 0.06317286 | 0 | 0.3166254 | 0.1334711 | 0.1368597 | 0.04442927 | 0.2469745 | 0.01561028 | 3.465771 | 0.04211524 | 0.136283 |
| TCGA-A8-A08B | 3.167123288 | 0 | 0.6667248 | 0.1341113 | 0 | 0 | 0 | 1.375636 | 0.05642241 | 0 | 0.5430338 | 0 | 9.879377 | 0 | 0.8347437 |
| TCGA-AR-A2LN | 3.180821918 | 0 | 0.604017879 | 0.353448289 | 0 | 0.195238102 | 0.211703967 | 0.435056019 | 3.754684805 | 0.579277886 | 1.490788678 | 0.101765034 | 4.004671712 | 0.366071442 | 0.380761307 |
| TCGA-GM-A3NY | 3.183561644 | 0 | 0.913410273 | 0.130332025 | 0 | 0.111988999 | 0.182150782 | 0.415915127 | 0.980890951 | 0.110758351 | 0.684095695 | 0.622641539 | 3.436826645 | 0.087491405 | 0.194138233 |
| TCGA-E9-A22B | 3.197260274 | 0 | 0.7101676 | 0.1148845 | 0.01315125 | 0.07014001 | 0.1774627 | 0.06946466 | 0.1602635 | 0.2312308 | 0.3142019 | 0.01624865 | 7.656222 | 0.0146125 | 0.1418562 |
| TCGA-E2-A14R | 3.216438356 | 0 | 0.5293149 | 1.693402 | 0.1543835 | 0 | 0.6802441 | 0.2621092 | 0.05972524 | 0.0775553 | 1.05384 | 0.2179933 | 4.894115 | 0.02450532 | 0.06796981 |
| TCGA-E2-A574 | 3.230136986 | 0 | 0.4782177 | 0.2398584 | 0 | 0 | 0.2095149 | 0.2296306 | 0.2943256 | 0 | 0.1416359 | 0.1879971 | 4.681251 | 0.2173717 | 0.1004864 |
| TCGA-BH-A0B3 | 3.295890411 | 0 | 0.2168915 | 0.2160605 | 0.1626686 | 0.1285283 | 0.104526 | 0.1670691 | 0.02447294 | 0 | 0.314051 | 0.1116558 | 4.35732 | 0.08033017 | 0.05570246 |
| TCGA-E2-A572 | 3.309589041 | 0 | 1.038834 | 0.5472784 | 0 | 0 | 0.7926845 | 0.3143652 | 0.586084 | 0 | 0 | 0.1336981 | 1.791033 | 0.02404708 | 0.06669881 |
| TCGA-EW-A1P1 | 3.315068493 | 0 | 0.2412675 | 0.5657307 | 0.02412675 | 0.21446 | 0.3720752 | 0.1911556 | 1.241388 | 0.254524 | 1.624462 | 0.1788547 | 4.4558 | 0.0268075 | 0.3717766 |
| TCGA-E9-A1RC | 3.353424658 | 0 | 0.675139 | 0.03325808 | 0.02411211 | 0.1071649 | 0.3021276 | 0.1910395 | 0.2775103 | 0.3391593 | 1.099774 | 0.02979102 | 5.552887 | 0.1205605 | 0.3715509 |
| TCGA-E2-A10A | 3.367123288 | 0 | 0.3378414 | 0.13203 | 0.3547335 | 0.09009105 | 0.5861345 | 0.223059 | 0.2973386 | 0.2970035 | 1.027283 | 0.08348205 | 4.417745 | 0.1313828 | 0.2082361 |
| TCGA-E9-A22H | 3.375342466 | 0 | 0.8167137 | 0.1466799 | 0.1020892 | 0.06805948 | 0.1967985 | 0.3201698 | 0.1555097 | 0.2692463 | 0.8314959 | 0.1103667 | 3.838921 | 0.04253717 | 0.1376484 |
| TCGA-AR-A2LQ | 3.378082192 | 0 | 0.2468356 | 0.2269752 | 0 | 0.2194094 | 0.2775661 | 0.2987831 | 0.1114067 | 0.1446655 | 0.7594941 | 0.05082843 | 2.845859 | 0.04571029 | 0.412053 |
| TCGA-E9-A22G | 3.394520548 | 0 | 0.8912176 | 0.145397 | 0.1581192 | 0.05110925 | 0.3602279 | 0.1898143 | 0.1751699 | 0.05054761 | 0.2185441 | 0.2308796 | 6.225735 | 0.1118015 | 0.7752527 |
| TCGA-BH-A0H9 | 3.416438356 | 0 | 0.4417483 | 0.03692775 | 0.0602384 | 0 | 0.07741481 | 0.2121187 | 0.13594 | 0.07060911 | 0.2180576 | 0.02480861 | 2.973726 | 0 | 0.09282322 |
| TCGA-A8-A095 | 3.498630137 | 0 | 0.8073582 | 0.1349815 | 0.2201886 | 0.0978616 | 0.2476016 | 0.290758 | 0.5714345 | 0.2580965 | 1.633979 | 0 | 3.749326 | 0 | 0.08482386 |
| TCGA-A2-A25B | 3.536986301 | 0 | 0.4633115 | 0.1217239 | 0.4964052 | 0.0294166 | 0.5103604 | 0.02185002 | 0.5377139 | 0.2909334 | 0.6109602 | 0.06133192 | 6.410558 | 0.05515613 | 0.2804729 |
| TCGA-E2-A1LL | 3.58630137 | 0 | 0.67499283 | 0.298405318 | 0.051922525 | 0.046153356 | 0.350320653 | 0.205690334 | 0.070304125 | 0.091292352 | 0.338318717 | 0.224529839 | 2.231398395 | 0.028845847 | 0.280031598 |
| TCGA-E2-A14U | 3.610958904 | 0 | 1.088125 | 0.4320662 | 0 | 0.117239 | 0.8263233 | 0.6531198 | 1.250109 | 0.173926 | 1.754607 | 0.2444366 | 3.9904 | 0 | 0.355669 |
| TCGA-A1-A0SE | 3.619178082 | 0 | 0.4271262 | 0.1435084 | 0.03285586 | 0.1168208 | 0.09500491 | 0.04338601 | 0.2446812 | 0.1733057 | 0.4995281 | 0.06089118 | 3.595609 | 0 | 0.3797146 |
| TCGA-E2-A3DX | 3.630136986 | 0 | 0.1872975 | 0.4090405 | 0.06243249 | 0.5272077 | 0.1805277 | 0.4946508 | 0.359273 | 0.5488571 | 1.017 | 0.2892625 | 3.540854 | 0.1040542 | 0.1202552 |
| TCGA-B6-A1KC | 3.632876712 | 0 | 2.64186 | 0.5050381 | 0.2502815 | 0.07415749 | 0.1876274 | 0.4957433 | 0.09413503 | 0.1955802 | 0.5435979 | 0.03435868 | 7.983685 | 0 | 0.2999629 |
| TCGA-GM-A3XG | 3.643835616 | 0 | 0.7597291 | 0.5398284 | 0 | 0.09208838 | 0.7988389 | 0.1824034 | 1.566412 | 0 | 0.7875431 | 0.3413314 | 3.790198 | 0.03837016 | 0.2128527 |
| TCGA-A2-A0SY | 3.690410959 | 0 | 0.606638353 | 0.242214951 | 0.031928334 | 0.056761483 | 0.215420087 | 0.168644847 | 0.302621167 | 0.336826385 | 0.24271313 | 0.098620338 | 3.958284571 | 0 | 0.245996798 |
| TCGA-AO-A03V | 3.701369863 | 0 | 0.3493575 | 0.09815921 | 0.01940875 | 0.03450445 | 0.1122434 | 0.6150999 | 0.2627983 | 0.2047517 | 0.2529285 | 0.1198996 | 4.877478 | 0.02156528 | 0.1794453 |
| TCGA-BH-A0W7 | 3.734246575 | 0 | 0.9948603 | 0.2652961 | 0.01989721 | 0.03537281 | 0.07671212 | 0.3678383 | 0.6196478 | 0.349841 | 0.4753721 | 0.2704176 | 4.539304 | 0.02210801 | 0.2146215 |
| TCGA-A8-A07Z | 3.756164384 | 0 | 0.4931064 | 0.1133578 | 0 | 0 | 0.142585 | 0.2604578 | 0.1669189 | 0.1734 | 0.3748501 | 0.03046217 | 1.611027 | 0.0273948 | 0.07598429 |
| TCGA-BH-A1FM | 3.802739726 | 1 | 0.3100493 | 0.04989298 | 0.1240197 | 0.0826798 | 0.2390741 | 0.02047093 | 0.1049532 | 0.1090283 | 0.8080922 | 0.07661449 | 8.269631 | 0.1722496 | 0.525541 |
| TCGA-E9-A1RH | 3.882191781 | 0 | 0.3681247 | 0.09764581 | 1.019422 | 0 | 0.08188131 | 0.2617499 | 0.2875662 | 0 | 0.09225541 | 0.05247991 | 4.56922 | 0 | 0.130905 |
| TCGA-E2-A109 | 3.882191781 | 0 | 0.9160938 | 0.1307149 | 0.110563 | 0.08423851 | 0.2435812 | 0.3337096 | 0.8982272 | 0.1666256 | 0.6861055 | 0.136603 | 13.43947 | 0.01754969 | 0.17037 |
| TCGA-E9-A1RE | 3.887671233 | 0 | 0.2294181 | 0.1054796 | 0.05735453 | 0.1784363 | 0.1382037 | 0.4165501 | 0.834836 | 0.2521078 | 0.5294264 | 0.1417255 | 13.84755 | 0 | 0.1546639 |
| TCGA-E2-A14N | 3.928767123 | 0 | 1.923784 | 0.6056893 | 0.209107 | 0.07434916 | 0.1209294 | 0.3589622 | 0.113254 | 0.2205964 | 0.9083381 | 0.671726 | 5.131317 | 0 | 0.1933317 |
| TCGA-3C-AALK | 3.967123288 | 0 | 0.5035924 | 0.2315368 | 0.03873788 | 0.1377347 | 0.4853903 | 0.4859553 | 0.3147111 | 0.2724422 | 1.766868 | 0.1914459 | 2.203205 | 0 | 0.298462 |
| TCGA-E9-A1RI | 3.969863014 | 0 | 0.7756051 | 0.149772 | 0.06204841 | 0.02757707 | 0.5681541 | 0.2458039 | 0.4620812 | 0.6545766 | 1.212892 | 0.09582766 | 4.317058 | 0.05170701 | 0.3585462 |
| TCGA-5L-AAT1 | 4.030136986 | 0 | 1.00352961 | 0.3844941 | 0 | 0.22300658 | 0.24181437 | 0.27607417 | 0.22646649 | 0 | 0.36326864 | 0.05166176 | 4.24859268 | 0.0464597 | 0.25772831 |
| TCGA-5L-AAT0 | 4.046575342 | 0 | 0.357899 | 0.3016773 | 0 | 0.1060442 | 0.5174444 | 0.4332203 | 0.1615341 | 0.2097577 | 1.230784 | 0.1473973 | 3.411625 | 0 | 0.4595814 |
| TCGA-AO-A0JI | 4.18630137 | 0 | 1.939851 | 0.1248639 | 0.03879701 | 0 | 0.5609206 | 0.2561563 | 0.4202554 | 0.2728581 | 0.50559 | 0.0719018 | 4.661065 | 0 | 0.05978352 |
| TCGA-A2-A3XZ | 4.197260274 | 0 | 1.729148 | 0.3663284 | 0.2373341 | 0.2109636 | 0.5228702 | 0.850653 | 0.2065854 | 0.6557425 | 0.5891162 | 0.3979567 | 2.827493 | 0.03767207 | 0 |
| TCGA-A2-A0YF | 4.205479452 | 0 | 0.08334769 | 0.2618587 | 0.02778256 | 0.2469561 | 0.1874486 | 0.05503011 | 0.263327 | 0.2930908 | 0.4827378 | 0.2059557 | 3.746138 | 0 | 0.2140551 |
| TCGA-AR-A24W | 4.246575342 | 0 | 1.038299 | 0.3045348 | 0.01790171 | 0.2546021 | 0.1725466 | 0.2600303 | 2.448166 | 0.5036085 | 0.8165124 | 0.2432974 | 5.837226 | 0.03978158 | 0.4413648 |
| TCGA-BH-A18U | 4.282191781 | 1 | 1.039742 | 0.09252431 | 0.1677003 | 0 | 0.06465554 | 0.1328685 | 0.3406043 | 0.1179431 | 0.2913888 | 0.06215919 | 5.773646 | 0.05590011 | 0.5943536 |
| TCGA-AO-A0J4 | 4.347945205 | 0 | 0.9555508 | 0.6644922 | 0.0836107 | 0.06370339 | 0.1611772 | 0.3943126 | 0.2749399 | 0.1680089 | 0.5447937 | 0.04427263 | 7.38107 | 0.17253 | 0.2024602 |
| TCGA-A2-A259 | 4.37260274 | 0 | 0.5547517 | 0.2763131 | 0.06163908 | 0.2739515 | 0.3861726 | 0.2848794 | 0.6468191 | 0.541882 | 0.9706064 | 0.0761564 | 12.25765 | 0.1369757 | 0.1899631 |
| TCGA-EW-A2FS | 4.394520548 | 0 | 1.454706 | 0.311084 | 0.05074557 | 0.1202858 | 0.8151899 | 0.2680371 | 5.153288 | 0.4758559 | 2.130855 | 0.4179815 | 3.437423 | 0.05638397 | 0.1563908 |
| TCGA-D8-A1JG | 4.416438356 | 0 | 0.3315746 | 0.1524481 | 0.3570803 | 0.0680153 | 0.1966708 | 0.0168401 | 0.3453527 | 0.4036073 | 0.5539708 | 0.06302576 | 6.491166 | 0.1558684 | 0.01965129 |
| TCGA-A8-A06T | 4.421917808 | 0 | 0.6382296 | 0.1148239 | 0 | 0.07399763 | 0.2942075 | 0.2748193 | 0.3757285 | 0.1951586 | 0.3917522 | 0.01714231 | 6.873689 | 0.01541617 | 0.2137973 |
| TCGA-A8-A076 | 4.498630137 | 0 | 0.7422975 | 0.07219516 | 0.299774 | 0 | 0.2751798 | 0.3016001 | 0.6378427 | 0.2007905 | 0.7751105 | 0.05291102 | 5.637573 | 0.01586106 | 0.2199672 |
| TCGA-AR-A2LK | 4.517808219 | 1 | 0.1526399 | 0.1122868 | 0.1526399 | 0.05427196 | 0.05884911 | 0.1007801 | 0.2066774 | 0.1073511 | 0.2652204 | 0.09429491 | 8.961783 | 0.01695999 | 0.2352075 |
| TCGA-D8-A1XO | 4.608219178 | 0 | 0.583052574 | 0.191478678 | 0.041646612 | 0.074038422 | 0.080282626 | 0.109988302 | 0.13157745 | 0.146449626 | 0.542725085 | 0.017151758 | 4.719533567 | 0 | 0.213915184 |
| TCGA-OL-A5DA | 4.884931507 | 0 | 0.566337 | 0.1902812 | 0.04356439 | 0.2323434 | 0.6298465 | 0.5752656 | 0.737338 | 0.3063869 | 1.75046 | 0.1076494 | 4.001921 | 0 | 0.2685188 |
| TCGA-AO-A0JJ | 5.169863014 | 0 | 0.6950561 | 0.3279757 | 0.05487285 | 0.03251725 | 0.105779 | 0.04830622 | 0.9906523 | 0 | 0.3575426 | 0.06779657 | 3.521213 | 0 | 0.5355168 |
| TCGA-HN-A2OB | 5.205479452 | 1 | 0.69532349 | 0.48952372 | 0 | 0.15451633 | 0.16754783 | 0.25823568 | 0.32363435 | 0.3056367 | 1.03826585 | 0.29531114 | 2.98194149 | 0.02414318 | 0.10044802 |
| TCGA-BH-A0AU | 5.243835616 | 0 | 0.7528225 | 0.1442189 | 0.0627352 | 0.07435283 | 0.04031178 | 0.3037523 | 0.3680937 | 0.2941431 | 0.6812873 | 0.05167378 | 6.20733 | 0 | 0.09667062 |
| TCGA-A2-A04V | 5.260273973 | 1 | 0.3770999 | 0.1182131 | 0.05142272 | 0.06094544 | 0.1982563 | 0.1131724 | 0.3249277 | 0.3013786 | 0.8562697 | 0.08471181 | 5.36186 | 0.01904545 | 0.3433684 |
| TCGA-BH-A0EI | 5.276712329 | 0 | 1.284581 | 0.7792796 | 0.0535242 | 0.06343609 | 0.06878613 | 0.1649164 | 0.07247283 | 0.250956 | 1.240018 | 0.08817372 | 4.971008 | 0 | 0.4123855 |
| TCGA-PE-A5DD | 5.350684932 | 0 | 1.075535 | 0.2697266 | 0.02444397 | 0.3476476 | 0.2827254 | 1.355682 | 2.283736 | 0.5157409 | 2.17673 | 0.2416084 | 1.792476 | 0 | 0.5273306 |
| TCGA-B6-A0IE | 5.460273973 | 1 | 0.8258786 | 0.2039207 | 0.09176429 | 0.02718942 | 0.08844751 | 0.2221529 | 1.180381 | 0.05378127 | 0.6975747 | 0.07558449 | 5.570913 | 0 | 0.2356707 |
| TCGA-A2-A0D0 | 5.610958904 | 0 | 0.2995331 | 0.1118946 | 0.03744164 | 0.1664073 | 1.623975 | 0.1483245 | 0.07604509 | 0.5266517 | 0.6505697 | 0.1387798 | 2.099573 | 0.04160183 | 0.1442375 |
| TCGA-BH-A0DV | 5.654794521 | 0 | 0.2675437 | 0.4305301 | 0 | 0.04756332 | 0.1031494 | 0.1766451 | 0.4165983 | 0.04704065 | 0.5229813 | 0 | 2.547475 | 0.02972708 | 0.2061332 |
| TCGA-B6-A0RG | 5.704109589 | 0 | 0.4829613 | 0.1332307 | 0.02414806 | 0 | 0.1862019 | 0.2550992 | 0.719333 | 0.1698325 | 0.4195863 | 0.02983544 | 4.012219 | 0 | 0.074421 |
| TCGA-E2-A152 | 5.830136986 | 0 | 0.262034599 | 0.086054056 | 0.018716757 | 0.099822704 | 0.072160991 | 0.123576938 | 0.050685717 | 0.329085839 | 0.406517801 | 0.023124951 | 3.689901668 | 0.041592794 | 0.086523692 |
| TCGA-BH-A0B5 | 5.852054795 | 0 | 0.9863138 | 0.1781519 | 0.1761275 | 0.1252462 | 0.4413797 | 0.3953783 | 1.025464 | 0.7432192 | 1.568411 | 0.1523265 | 3.26403 | 0.03913944 | 0.2985403 |
| TCGA-AO-A12E | 5.868493151 | 0 | 0.6024952 | 0.2203483 | 0.02738615 | 0.09737297 | 0.1319814 | 0.5424491 | 1.130982 | 0.722272 | 0.8327371 | 0.1522627 | 4.075518 | 0.01521453 | 0.1477006 |
| TCGA-GM-A2DF | 5.904109589 | 0 | 0.301146083 | 0.969205784 | 0.086041738 | 0.267685407 | 1.078113343 | 0.454470803 | 0.262129977 | 0.226923265 | 1.448304367 | 0.318919183 | 3.536173674 | 0.310706276 | 0 |
| TCGA-AR-A255 | 5.920547945 | 0 | 0.6253039 | 0.2299968 | 0.09379559 | 0.194539 | 0.1808108 | 0.3922126 | 0.6561722 | 0.2748589 | 0.882782 | 0.09657203 | 4.517922 | 0.1042173 | 0.1445325 |
| TCGA-GM-A4E0 | 6.002739726 | 0 | 0.593453778 | 1.030775399 | 0 | 0.586127188 | 0.635559601 | 0.740116586 | 1.741020955 | 0 | 2.577898762 | 0.366612372 | 2.391328696 | 0 | 0 |
| TCGA-BH-A18M | 6.046575342 | 1 | 0.6921414 | 0.1674873 | 0.05464274 | 0.06476177 | 0.1404472 | 0.3126738 | 0.4192617 | 0.5124008 | 0.6329657 | 0.06751227 | 3.285294 | 0.04047611 | 0.1122676 |
| TCGA-B6-A1KI | 6.126027397 | 0 | 0.3523557 | 0.2492348 | 0 | 0.0722781 | 0.1044985 | 0.2147465 | 0.4403969 | 0.1906236 | 0.8830356 | 0.1339517 | 2.437897 | 0.09034763 | 0.1252975 |
| TCGA-A2-A04T | 6.153424658 | 0 | 0.4755572 | 0.6122116 | 0.01585191 | 0.3099929 | 0.4583684 | 0.230256 | 0.4292759 | 0.2787149 | 0.3442948 | 0.09792684 | 4.338183 | 0.1232926 | 0.3419737 |
| TCGA-BH-A0BQ | 6.178082192 | 0 | 0.359881418 | 0.206828401 | 0 | 0 | 0.086718414 | 0.11880542 | 0.213187864 | 0.158189635 | 0.537379494 | 0.027790071 | 2.641166676 | 0 | 0.173297633 |
| TCGA-B6-A402 | 6.249315068 | 0 | 2.696961 | 0.1256738 | 0.2368952 | 0.1619796 | 0.7025622 | 0.5293859 | 0.09869562 | 0.3203993 | 3.087141 | 0.3152036 | 13.27246 | 0.222722 | 0.3931191 |
| TCGA-GM-A2DD | 6.252054795 | 0 | 0.5193154 | 0.2571323 | 0.719052 | 0.1775437 | 0.1540138 | 0.0791254 | 0.6761185 | 0.1404741 | 0.5639624 | 0.222101 | 2.498553 | 0.1331578 | 0.09233413 |
| TCGA-BH-A0BR | 6.383561644 | 0 | 0.1581827 | 0.0727277 | 0.07909137 | 0.1054552 | 0.2286979 | 0.3133193 | 0.1606369 | 0 | 0.8589108 | 0.04885953 | 1.686861 | 0.04393965 | 0.1828115 |
| TCGA-BH-A0DL | 6.523287671 | 0 | 1.379348072 | 0.184970048 | 0.071841045 | 0 | 0.249279772 | 0.341516717 | 0.233458066 | 0.353678993 | 1.185864859 | 0.213026729 | 8.002009287 | 0.143682091 | 0.132842511 |
| TCGA-AR-A1AJ | 6.528767123 | 0 | 1.32638489 | 0.45275416 | 0.160773926 | 0.285820313 | 0.15496282 | 0.371527092 | 0.054422768 | 0.848038291 | 1.222172832 | 0.0993198 | 3.21387319 | 0.11164856 | 0.216773833 |
| TCGA-A2-A0EO | 6.690410959 | 0 | 0.4511856 | 0.2148503 | 0.03222754 | 0.0286467 | 0.2485015 | 0.1276689 | 0.7636428 | 0.2266552 | 0.1399929 | 0.03981781 | 4.525693 | 0 | 0.1489813 |
| TCGA-BH-A0B0 | 6.78630137 | 0 | 0.22102481 | 0.20324121 | 0.01841873 | 0 | 0.17752997 | 0.51075885 | 0.67336191 | 0.12953835 | 1.44016168 | 0.13654043 | 1.97153104 | 0 | 0.28381999 |
| TCGA-AO-A12D | 6.890410959 | 0 | 0.6708488 | 0.2024113 | 0 | 0.07453875 | 0.3637132 | 0.1384145 | 0.1135428 | 0.1474393 | 0.7285236 | 0.07770449 | 5.559705 | 0.06988008 | 0.1615206 |
| TCGA-BH-A1ET | 6.904109589 | 1 | 0.394951 | 0.1765426 | 0.02194172 | 0.1365263 | 0.2114865 | 0.1014088 | 0.7873024 | 0.3857885 | 0.9054683 | 0.06777366 | 4.576322 | 0.01218985 | 0.4226335 |
| TCGA-BH-A204 | 6.942465753 | 1 | 1.538913 | 0.1424919 | 0.08549516 | 0 | 0.1442087 | 0.04233598 | 0.5932809 | 0.263062 | 0.394593 | 0.02640777 | 11.69017 | 0.01187433 | 0.1646777 |
| TCGA-GM-A2DC | 6.945205479 | 0 | 1.67326 | 0.09926645 | 0.02698807 | 0.2878727 | 0.05202519 | 0.3207385 | 0.5481356 | 0.1898062 | 0.6447828 | 0.1667217 | 9.675283 | 0.02998674 | 0.2495205 |
| TCGA-AR-A0U2 | 6.989041096 | 1 | 1.13178 | 0.1836561 | 0.4327396 | 0.1183561 | 0.1604225 | 0.4615401 | 1.487381 | 0.5267498 | 0.6506909 | 0.08225522 | 8.115342 | 0.1479452 | 0.102588 |
| TCGA-GM-A2DO | 7.112328767 | 0 | 1.183274 | 0.7933832 | 0.0986062 | 0.08764995 | 1.900842 | 1.692717 | 0.2002721 | 0 | 1.927506 | 0.7918953 | 4.505461 | 0.05478122 | 0.1519453 |
| TCGA-AR-A1AO | 7.17260274 | 0 | 0.6615985 | 0.3110965 | 0.01503633 | 0.106925 | 0.4347854 | 0.397108 | 0.1017974 | 0.4758751 | 0.5225295 | 0.2229325 | 3.16371 | 0.1336563 | 0.09267979 |
| TCGA-PE-A5DE | 7.246575342 | 0 | 0.563344304 | 0.431681459 | 0.023472679 | 0.083458415 | 0.633479538 | 0.21696892 | 0.381389656 | 0.41270645 | 0.917664929 | 0.029000994 | 2.437502396 | 0.05216151 | 0.108509336 |
| TCGA-GM-A2DK | 7.246575342 | 0 | 0.7352406 | 0.3098715 | 0.0408467 | 0 | 0.1968516 | 0.1887825 | 0.6913405 | 0 | 0.4879425 | 0.1514009 | 3.468401 | 0.02269261 | 0.2202968 |
| TCGA-A2-A0CQ | 7.383561644 | 0 | 0.9269677 | 0.07348139 | 0.0319644 | 0.142064 | 0.3080906 | 0.5698194 | 0.6275663 | 0.6182127 | 1.562061 | 0.09873175 | 2.172877 | 0.124306 | 0.2955296 |
| TCGA-B6-A0RT | 7.454794521 | 0 | 1.022317953 | 0.853890091 | 0.272618121 | 0.151454512 | 0.788293362 | 0.854978426 | 0.13842387 | 0.299580353 | 1.776335267 | 0.231567516 | 5.80079097 | 0.18931814 | 0.183787497 |
| TCGA-AC-A2FF | 7.55890411 | 0 | 0.5817351 | 0.4152737 | 0.1224705 | 0.02721568 | 0.3836427 | 0.2425826 | 0.1036422 | 0.7536649 | 0.8644979 | 0.2080581 | 4.837678 | 0.0170098 | 0.3066678 |
| TCGA-AO-A1KP | 8.090410959 | 0 | 1.93312 | 0.1311331 | 0.06338098 | 0.02816933 | 0.7330812 | 0.3347771 | 0.7509171 | 0.3343173 | 1.376601 | 0.2153485 | 8.999277 | 0 | 0.4150793 |
| TCGA-BH-A1F6 | 8.123287671 | 1 | 0.33786977 | 0.898766875 | 0.09653422 | 0.128712293 | 0.139567547 | 0.063736486 | 0.09803193 | 0.254595745 | 0.943501878 | 0.02981752 | 4.298977743 | 0 | 0.111564428 |
| TCGA-AR-A0TQ | 8.194520548 | 0 | 0.2906977 | 0.1883308 | 0.1057083 | 0.1409443 | 0.1528312 | 0.2966229 | 0.6977639 | 0.557582 | 0.8322732 | 0.163256 | 4.485872 | 0.05872681 | 0.04072228 |
| TCGA-AR-A24Z | 8.221917808 | 0 | 0.7365229 | 0.05820224 | 0.2992124 | 0.02045897 | 0.06655327 | 0.2127508 | 0.1869875 | 0.283278 | 0.7498536 | 0.1421859 | 8.449973 | 0.01278686 | 0.5319989 |
| TCGA-AR-A1AQ | 8.276712329 | 0 | 0.669753584 | 0.769831705 | 0.025759753 | 0.13738535 | 0.893832401 | 0.06803126 | 0.139516858 | 0.181167495 | 0.447590282 | 0.159133611 | 4.498118465 | 0.085865844 | 0.277858012 |
| TCGA-B6-A1KF | 8.460273973 | 0 | 0.4395092 | 0.1111403 | 0.06592638 | 0 | 0.4659856 | 0 | 0.3868177 | 0.231829 | 1.336426 | 0.02715115 | 11.92621 | 0.0976687 | 0 |
| TCGA-GM-A2DN | 8.468493151 | 0 | 0.861380396 | 0.387036124 | 0.058730482 | 0.174016242 | 0.18869231 | 0.12925553 | 0.132537053 | 0.34420795 | 1.148034752 | 0.193500686 | 3.265215389 | 0.065256091 | 0.271498855 |
| TCGA-A2-A0EM | 8.476712329 | 0 | 0.3491504 | 0.08026446 | 0.0872876 | 0.06207119 | 0.03365305 | 0.09221029 | 0.1181891 | 0.6138908 | 0.4929182 | 0.08627655 | 3.890683 | 0.01939725 | 0.1883058 |
| TCGA-AR-A1AK | 8.654794521 | 0 | 0.8346714 | 0.3933509 | 0.08346714 | 0.296772 | 0.2815759 | 0.08266346 | 1.045399 | 0.3668885 | 0.8157874 | 0.2578135 | 5.757274 | 0 | 0.353697 |
| TCGA-A2-A25E | 8.778082192 | 0 | 1.098843 | 0.08915562 | 0.1292756 | 0.1723675 | 0.1869046 | 0.1493694 | 0.98461 | 0.738718 | 1.193314 | 0.2395842 | 7.907565 | 0 | 0.09960243 |
| TCGA-GM-A2DM | 8.838356164 | 0 | 0.6301568 | 0.0852139 | 0.01853402 | 0 | 0.2858259 | 0.1957927 | 0.175668 | 0.2606983 | 0.4428038 | 0.1831934 | 4.832355 | 0.04118672 | 0.2570368 |
| TCGA-Z7-A8R6 | 8.920547945 | 0 | 0.591959643 | 0.102062007 | 0.055496217 | 0.098659941 | 0.67754417 | 0.170992469 | 0.801525046 | 0.13010102 | 0.76338687 | 0.251411431 | 3.508018454 | 0.041108309 | 0.171031679 |
| TCGA-AR-A0TZ | 8.936986301 | 1 | 0.3496611 | 0.06889875 | 0.1998064 | 0.2442078 | 0.09629222 | 0.1978825 | 1.318891 | 0.6147888 | 0.6509529 | 0.04628719 | 5.011619 | 0.01387544 | 0.01924299 |
| TCGA-Z7-A8R5 | 9.005479452 | 0 | 0.7778049 | 0.8046257 | 0 | 0.04938444 | 1.552932 | 0.5502255 | 1.090776 | 0 | 0 | 0.2402486 | 3.452719 | 0 | 0.342441 |
| TCGA-AR-A1AI | 9.030136986 | 0 | 0.915916849 | 0.36442315 | 0.123296499 | 0.187880379 | 0.645131423 | 0.4884373 | 0.190795308 | 0.37163152 | 1.759784548 | 0.10881103 | 2.276513633 | 0.019570873 | 0.162849767 |
| TCGA-AO-A126 | 9.060273973 | 0 | 0.2758248 | 0.214002 | 0.05171714 | 0.0306472 | 0.3987828 | 0.2276408 | 0.4668402 | 0.06062083 | 1.085826 | 0.1916929 | 2.277654 | 0 | 0.1062567 |
| TCGA-BH-A42U | 9.216438356 | 0 | 0.9599578 | 0.6620399 | 0 | 0.02752567 | 1.223732 | 0.7564827 | 1.551376 | 0 | 0.571687 | 0.1339087 | 3.255258 | 0.03440709 | 0.4771705 |
| TCGA-AO-A125 | 9.468493151 | 0 | 1.23835 | 0.09489274 | 0.01876288 | 0.03335624 | 0.9765742 | 0.3468682 | 1.168644 | 1.517526 | 3.260157 | 0.1854555 | 1.744583 | 0 | 0.2602108 |
| TCGA-BH-A1ES | 9.484931507 | 1 | 0.42356585 | 0.10171601 | 0.008301765 | 0 | 0.84818 | 0.263330815 | 0.21644405 | 0.08757905 | 0.44195005 | 0.180806905 | 5.8986045 | 0.066924185 | 0.21924005 |
| TCGA-BH-A1FC | 9.512328767 | 1 | 0.1577158 | 0.2392929 | 0.03154315 | 0.05607671 | 0.3040304 | 0.1666103 | 0.2776153 | 0 | 0.5138251 | 0.03897223 | 8.274604 | 0.03504794 | 0.1944233 |
| TCGA-AO-A124 | 9.605479452 | 0 | 0.6365447 | 0.1399699 | 0.08302757 | 0.2214069 | 0.08002658 | 0.1644563 | 0.05621049 | 0.0973217 | 0.4508285 | 0.1538735 | 2.743544 | 0.09225286 | 0.2132329 |
| TCGA-GM-A2DL | 9.64109589 | 0 | 0.430313287 | 0.128599373 | 0.064546993 | 0.03825007 | 0.248855877 | 0.284113312 | 0.174795806 | 0.151318958 | 0.747693674 | 0 | 2.151781267 | 0.071718881 | 0.364695691 |
| TCGA-AR-A24O | 9.882191781 | 0 | 0.7539972 | 0.4521722 | 0.01639124 | 0.2913999 | 0.1579879 | 0.3246684 | 1.287256 | 0.3458372 | 1.352834 | 0.1215104 | 3.993473 | 0.05463748 | 0.1515466 |
| TCGA-AR-A24M | 10.02739726 | 0 | 0.4733489 | 0.1660873 | 0.01245655 | 0.06643493 | 0.192101 | 0.1644881 | 0.03373283 | 0.1314097 | 0.4599341 | 0.07695166 | 3.635845 | 0.01384061 | 0.6334245 |
| TCGA-B6-A0IM | 10.6109589 | 1 | 0.4335446 | 0.2462323 | 0.1020105 | 0.045338 | 0.3932935 | 0.1515424 | 0.08632765 | 0.1793591 | 0.6646838 | 0.04726355 | 6.34981 | 0.01416813 | 0.1571911 |
| TCGA-B6-A0X0 | 10.80821918 | 1 | 0.3128027 | 0.0431452 | 0 | 0 | 0.1205986 | 0.04130545 | 0.08470822 | 0 | 0.2717562 | 0.03864744 | 1.97895 | 0 | 0.2410037 |
| TCGA-BH-A209 | 10.84657534 | 1 | 0.4621795 | 0.2257773 | 0.1444311 | 0 | 0.2227371 | 0.4768014 | 0.09778128 | 0.1015779 | 1.568483 | 0.3568954 | 7.187664 | 0.0160479 | 0.2448142 |
| TCGA-3C-AALI | 10.97260274 | 0 | 2.455906 | 0.2509227 | 0.08186353 | 0.09702345 | 1.315077 | 0.4324016 | 0.4803276 | 0.4797863 | 3.437528 | 0.4045765 | 2.916516 | 0.06063965 | 0.1681947 |
| TCGA-A2-A0EN | 11.2 | 0 | 0.440956596 | 0.337897775 | 0.014698553 | 0.365830658 | 0.283345604 | 0.582281062 | 0.437846888 | 0.516872201 | 1.213129459 | 0.054481124 | 2.611249712 | 0.130653806 | 0.135896736 |
| TCGA-AR-A0TP | 11.71232877 | 0 | 1.327222 | 0.6254726 | 0.2488542 | 0.4129136 | 0.1918876 | 0.06572215 | 0.3144899 | 0.05833944 | 0.7927301 | 0.1024882 | 6.371727 | 0 | 0.1789513 |
| TCGA-B6-A0RO | 13.50410959 | 0 | 0 | 0.125356 | 0 | 0 | 0.3795921 | 0.2600232 | 0.1640767 | 0.05326482 | 0 | 0.03742933 | 2.240845 | 0 | 0.2100669 |
| TCGA-B6-A0IO | 13.81369863 | 0 | 2.007357 | 0.1118694 | 0.1064507 | 0.05407021 | 0.1172607 | 0.3614597 | 0.8648183 | 0.6951884 | 0.4624104 | 0.05636663 | 3.097632 | 0.05069082 | 0.7732995 |
| TCGA-B6-A0RE | 21.30684932 | 0 | 0.37947647 | 0.113406761 | 0.037947647 | 0.168656209 | 0.036576045 | 0.325712954 | 0.128454658 | 0 | 0.206050572 | 0.117212809 | 4.889430222 | 0.063246078 | 0.087712057 |
| TCGA-B6-A0RU | 23.57534247 | 0 | 0.7988003 | 0.5091622 | 0.4175547 | 0.09682428 | 0.1749836 | 0.191784 | 0.07374487 | 0 | 0.07886141 | 0.06729101 | 4.280958 | 0.2824042 | 0.3636739 |
